# PFKFB3 controls acinar IP3R-mediated Ca^2+^ overload to regulate acute pancreatitis severity

**DOI:** 10.1172/jci.insight.169481

**Published:** 2024-05-23

**Authors:** Tan Zhang, Shengchuan Chen, Liang Li, Yuepeng Jin, Siying Liu, Zhu Liu, Fengyu Shi, Lifen Xie, Panpan Guo, Andrew C. Cannon, Akmal Ergashev, Haiping Yao, Chaohao Huang, Baofu Zhang, Lijun Wu, Hongwei Sun, Siming Chen, Yunfeng Shan, Zhengping Yu, Ezequiel J. Tolosa, Jianghuai Liu, Martin E. Fernandez-Zapico, Feng Ma, Gang Chen

**Affiliations:** 1 Zhejiang Key Laboratory of intelligent Cancer Biomarker Discovery & Translation, Department of Hepatopancreatobiliary Surgery, The First Affiliated Hospital of Wenzhou Medical University, Wenzhou, China.; 2 National Key Laboratory of Immunity and Inflammation, and CAMS Key Laboratory of Synthetic Biology Regulatory Elements, Suzhou Institute of Systems Medicine (ISM), Chinese Academy of Medical Sciences & Peking Union Medical College, Suzhou, China.; 3 State Key Laboratory of Pharmaceutical Biotechnology and MOE key laboratory of Model Animal for Disease Study, Model Animal Research Center of Nanjing University, Nanjing, China.; 4 Department of Laboratory Medicine and Pathology, Mayo Clinic, Rochester, Minnesota, USA.; 5 State Key Laboratory of Cellular Stress Biology and Fujian Provincial Key Laboratory of Innovative Drug Target Research, School of Pharmaceutical Sciences, Xiamen University, Xiamen, Fujian, China.; 6 Schulze Center for Novel Therapeutics, Division of Oncology Research, Department of Oncology, Mayo Clinic, Rochester, Minnesota, USA.

**Keywords:** Inflammation, Metabolism, Calcium signaling

## Abstract

Acute pancreatitis (AP) is among the most common hospital gastrointestinal diagnoses; understanding the mechanisms underlying the severity of AP is critical for development of new treatment options for this disease. Here, we evaluate the biological function of phosphofructo-2-kinase/fructose-2,6-biphosphatase 3 (PFKFB3) in AP pathogenesis in 2 independent genetically engineered mouse models of AP. PFKFB3 was elevated in AP and severe AP (SAP), and KO of *Pfkfb3* abrogated the severity of alcoholic SAP (FAEE-SAP). Using a combination of genetic, pharmacological, and molecular studies, we defined the interaction of PFKFB3 with inositol 1,4,5-trisphosphate receptor (IP3R) as a key event mediating this phenomenon. Further analysis demonstrated that the interaction between PFKFB3 and IP3R promotes FAEE-SAP severity by altering intracellular calcium homeostasis in acinar cells. Together, our results support a PFKFB3-driven mechanism controlling AP pathobiology and define this enzyme as a therapeutic target to ameliorate the severity of this condition.

## Abstract 2

Interaction of phosphofructo-2-kinase/fructose-2,6-biphosphatase 3 with the inositol 1,4,5-trisphosphate receptor promotes acute pancreatitis by altering intracellular calcium homeostasis in acinar cells.

## Graphical abstract

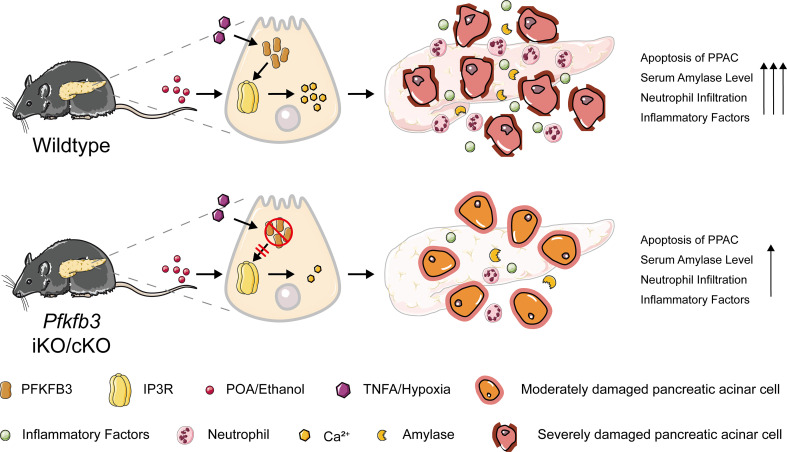

## Introduction

Acute pancreatitis (AP) represents the most common gastrointestinal inflammatory process requiring hospitalization and costs the US health care system nearly $3 billion annually (1). Globally, the incidence of AP is about 34 per 100,000 a year, affecting females and males in equal proportion (2). Clinically, AP presents as severe abdominal pain and elevated serum amylase and/or lipase levels (3). In general, patients with mild presentations of AP recover fully in days to weeks; however, approximately 20%–30% of patients develop severe or moderately severe AP, often resulting in multiple, persistent organ failure and local complications (4, 5). Thus, understanding the mechanisms contributing to AP severity could facilitate development of new treatment approaches to improve the outcomes of patients with this disease.

Alcohol-related AP is a common type of AP arising as a result of heavy alcohol consumption. In alcohol-related AP, fatty acid ethyl esters (FAEE) from nonoxidative ethanol metabolism cause pancreatic damage and inflammation (6–10). In this study, we provide evidence for the role of phosphofructo-2-kinase/fructose-2,6-biphosphatase 3 (PFKFB3) as a regulator of severity in alcohol-related AP. PFKFB3, encoded by the *PFKFB3* gene, plays a key role in maintaining glycolytic rates (11) by controlling the production of fructose-2,6-bisphosphate (F-2,6-BP), an allosteric activator of phosphofructokinase-1 (PFK-1), which is the rate-limiting enzyme of glycolysis (12, 13). Here, we show that PFKFB3 expression is increased in AP and SAP. In mice, KO of *Pfkfb3* reduced serum amylase and histologic indicators of pancreatic injury and inflammation in a FAEE-SAP model induced by injection of palmitoleic acid (POA) and ethanol. Interestingly, this effect of PFKFB3 loss was not mediated by dysregulation of glycolysis but through inositol 1,4,5-trisphosphate receptor–mediated (IP3R-mediated) Ca^2+^ overload, which has been shown to be a central event in AP pathogenesis (14). Further analysis demonstrates that the interaction between PFKFB3 and IP3R in pancreatic acinar cells promotes pathologic intracellular calcium signaling. In conclusion, our data indicate that PFKFB3 controls the severity of the AP model by affecting calcium levels in an IP3R-dependent manner and define PFKFB3 as a promising therapeutic target to improve outcomes in patients with alcohol-related AP.

## Results

### Elevated PFKFB3 expression during AP and SAP progression.

**Figure 1:**
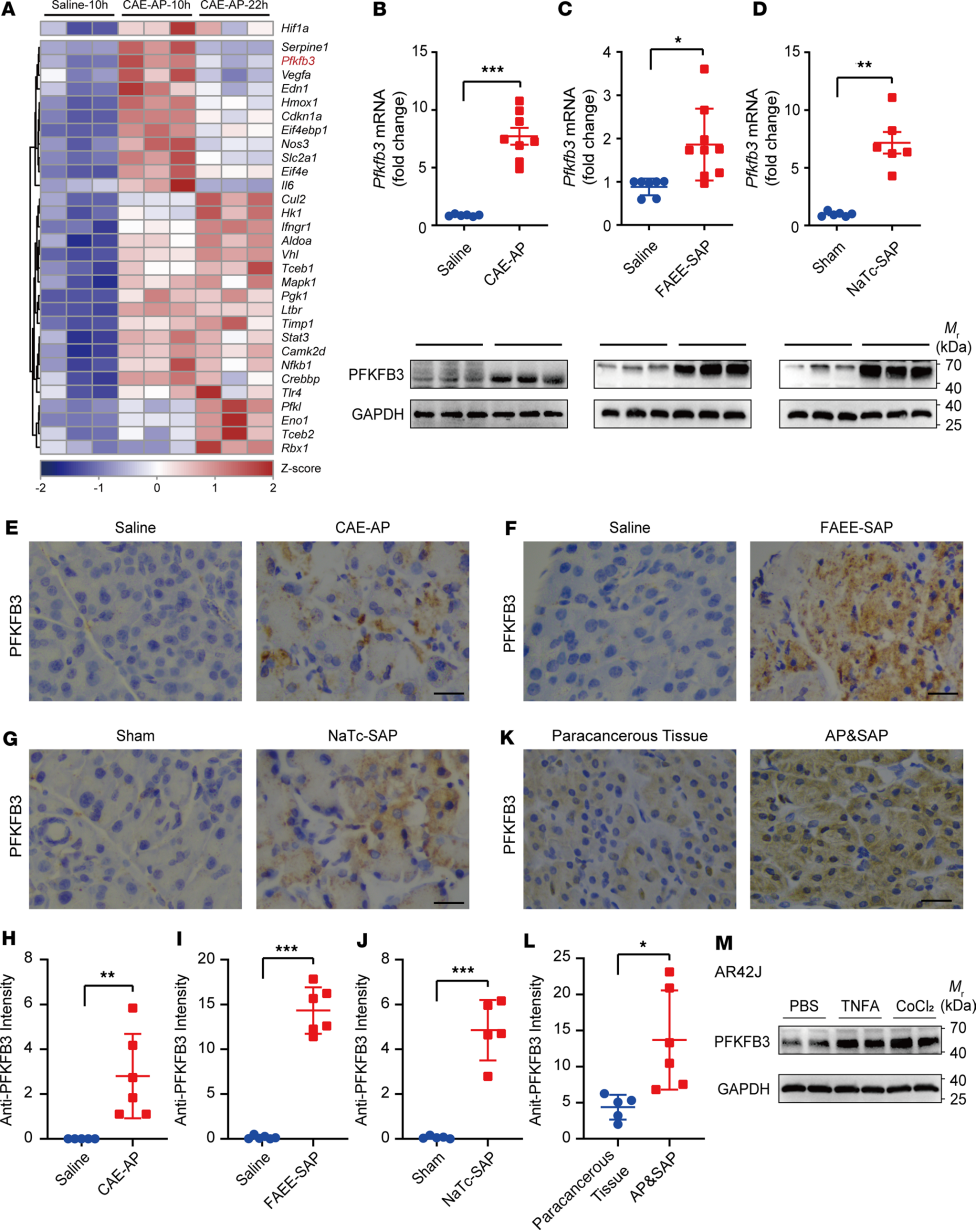
PFKFB3 is upregulated in AP and SAP. (**A**) Pancreatic tissues were collected 10 or 22 hours after injection of saline or caerulein (CAE), followed RNA-Seq (the National Genomics Data Center Accession no. CRA002365). Heatmap shows differentially expressed genes (DEGs) of HIF1 signaling pathway in CAE-AP model. (**B**–**D**) Western blotting and qPCR detected the PFKFB3 level in CAE-AP (**B**), FAEE-SAP (**C**) and NaTc-SAP (**D**) models. (**E**–**J**) IHC analysis of PFKFB3 in pancreas from control group (*n* = 5) and CAE-AP (**E**), FAEE-SAP (**F**), and NaTc-SAP (**G**) models (*n* = 5). Quantification of PFKFB3 intensity in 3 AP models is shown in **H**–**J**, respectively. Scale bar: 200 μm. (**K** and **L**) IHC analysis of PFKFB3 in pancreas from patients with AP or SAP (*n* = 5). Scale bar: 80 μm. (**M**) PFKFB3 levels were detected using Western blotting in AR42J cells treated with TNFA (80 ng/mL; 6 hours) and CoCl_2_ (200 μmol/L; 6 hours). *P* values calculated using unpaired Student’s *t* test. *P < 0.05, **P < 0.01, ***P < 0.001. Error bars show mean ± SD. Each experiment was performed at least in triplicate.

To define the genes involved in the regulation of AP progression, we analyzed the transcriptome of pancreata from vehicle control and caerulein-induced AP (CAE-AP) mice using bulk RNA-Seq (Supplemental Figure 1A; supplemental material available online with this article; https://doi.org/10.1172/jci.insight.169481DS1). *Pfkfb3*, a known regulator of glycolysis, was among the most upregulated genes in AP mice (Figure 1A and Supplemental Figure 1B). Because of the central role of glycolysis as one of the main sources of ATP in acinar cells under physiological and disease conditions (15–17), we focused our studies on PFKFB3. Induction of PFKFB3 transcripts and protein levels were confirmed by quantitative PCR (qPCR) and Western blotting (WB), respectively (Figure 1B and Supplemental Figure 1C). To confirm the biological meaning of these findings, we examined the expression of PFKFB3 in an in vitro system and in 2 additional AP models, a FAEE-SAP (Supplemental Figure 1D) and sodium taurocholate biliary pancreatitis (NaTc-SAP) (Supplemental Figure 1E). A substantial induction of *Pfkfb3* mRNA and protein was observed during FAEE-SAP (Figure 1C) and NaTc-SAP (Figure 1D). IHC and immunofluorescence (IF) demonstrated induction of PFKFB3 in the acinar compartment of all 3 models (Figure 1, E–J; Supplemental Figure 1, F and G; and Supplemental Figure 2, A–C) as well as in patients with AP (Figure 1, K and L, and Supplemental Figure 2D). These assays also show that PFKFB3 can localize to both the cytoplasm and nucleus of exocrine cells during AP development. In vitro, TNFA — a proinflammatory factor implicated in AP (18) — and CoCl_2_ — an inducer of hypoxia, a known component of AP pathogenesis (19, 20) — induced PFKFB3 in acinar cell lines (Figure 1M and Supplemental Figure 2E). In summary, our finding identify and confirm *Pfkfb3* as one of the most elevated genes in AP models and suggest a role for this molecule in AP pathogenesis.

### Loss PFKFB3 alleviates FAEE-SAP severity.

**Figure 2:**
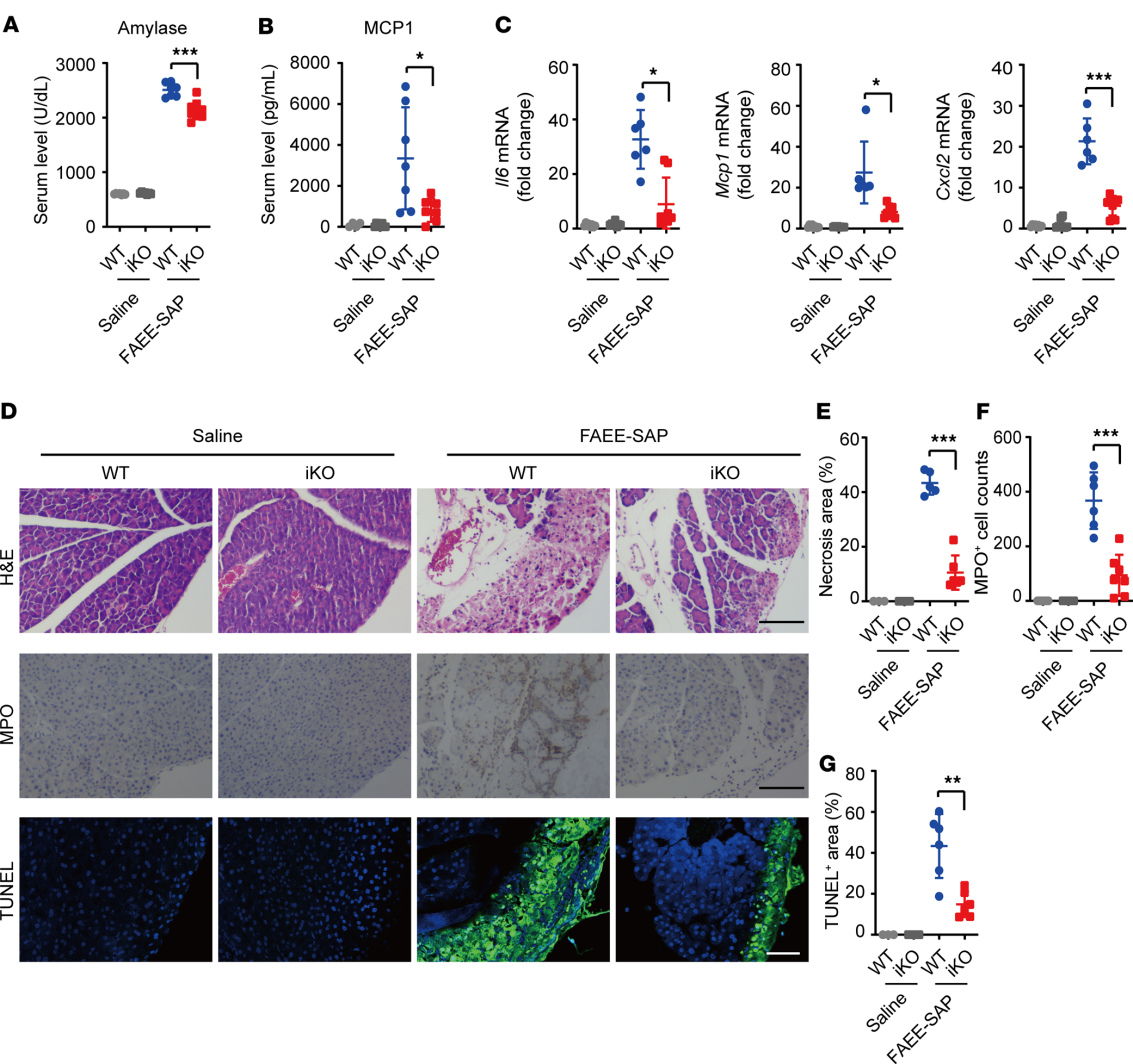
Loss of *Pfkfb3* lowers the severity of FAEE-SAP. (**A** and **B**) Serum amylase (**A**) and MCP1 (**B**) were detected using iodine-starch colorimetry and ELISA, respectively, in FAEE-SAP and control mice (*n* = 4–8). (**C**) Relative transcription level of key inflammatory genes, *Il6*, *Mcp1*, and *Cxcl2*, were measured using qPCR in pancreata from FAEE-SAP and control mice (*n* = 6–8). (**D**) H&E, MPO, and TUNEL staining in AP and normal pancreas. Scale bar: 100 μm. (**E**–**G**) Quantification of necrotic area (**E**)**,** MPO^+^ cells (**F**), and apoptotic cell area (**G**) (*n* = 3–8). *P* values calculated with unpaired Student’s *t* test. *P < 0.05, **P < 0.01, ***P < 0.001. Data are shown as mean ± SD. Each experiment was performed at least in triplicate.

To interrogate the role of *Pfkfb3* in AP pathobiology, we used Cre-estrogen receptor, tamoxifen–driven (CAAG-Cre-ER^TM^–driven) *Pfkfb3* inducible KO (iKO) mice in the FAEE-SAP model. No differences in pancreatic/body weight ratio or sex were found in healthy iKO and WT animals (Supplemental Figure 3, A–G). However, after SAP induction, serum amylase and MCP1 levels were lower in the iKO group compared with WT mice (Figure 2, A and B). qPCR showed decreased expression of *Il6*, *Mcp1*, and *Cxcl2* in the iKO relative to WT mice (Figure 2C). iKO pancreata had decreased proportional pancreatic necrosis in comparison with WT counterparts in the FAEE-SAP model. Furthermore, iKO showed reduced pancreatic neutrophil infiltration and apoptosis as determined by myeloperoxidase (MPO) and TUNEL staining, respectively (Figure 2, D–G, and Supplemental Figure 4, A–D). Next, we evaluate the effect of *Pfkfb3* KO in a necrotizing pancreatitis model using retrograde injection of NaTc. There were no marked differences in the serum amylase and MCP1 levels or expression of inflammatory cytokines in SAP progression between WT and iKO mice (Supplemental Figure 5, A–C). H&E and MPO stains showed the same degree of injury and acute inflammation among iKO and WT mice (Supplemental Figure 5, D–F), and survival rates were not different between the 2 groups (Supplemental Figure 5G).

**Figure 3:**
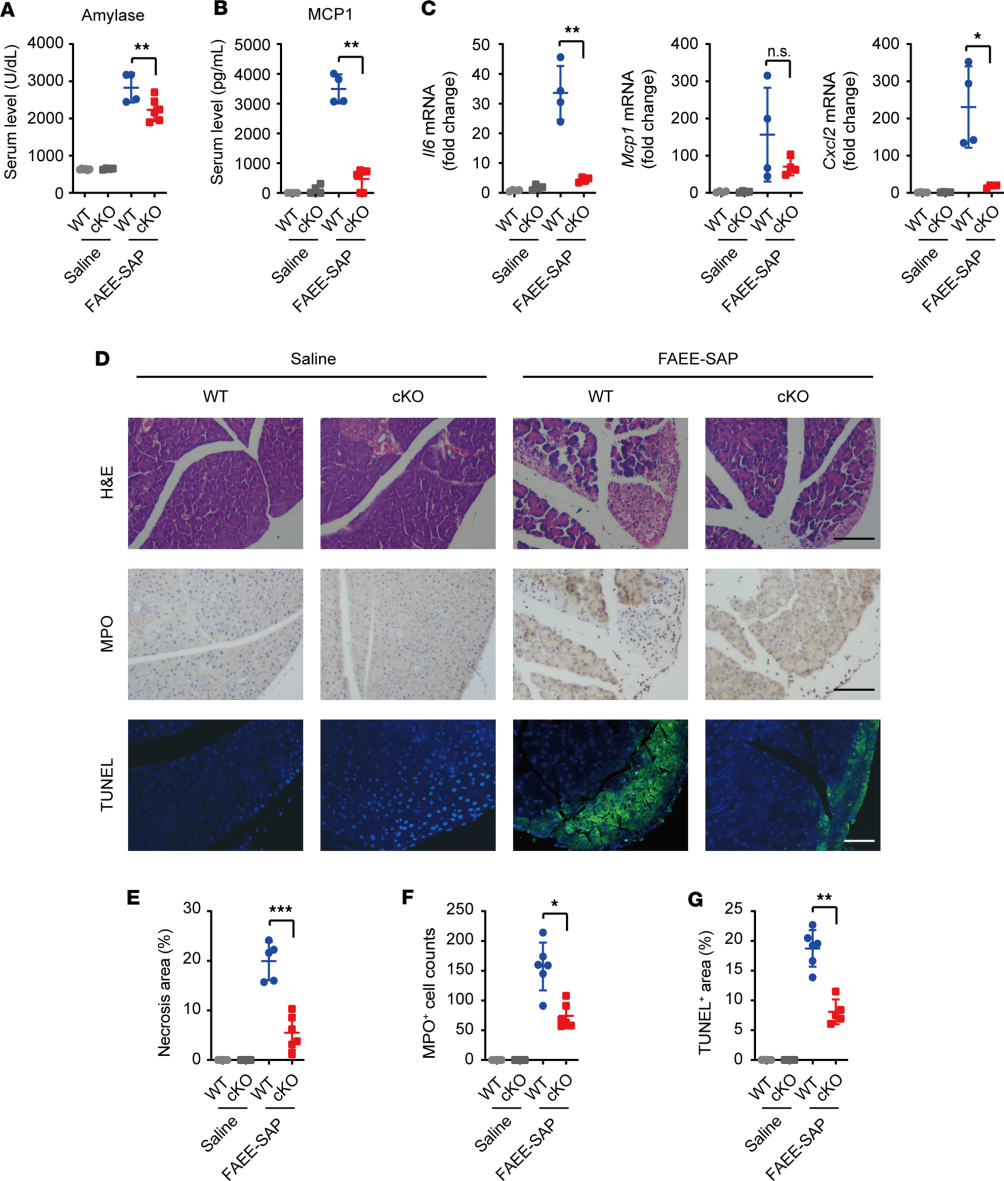
Pancreas-specific cKO of *Pfkfb3* protects mice from the FAEE-induced damage. (**A** and **B**) Serum amylase (**A**) and MCP1 (**B**) were detected using iodine-starch colorimetry and ELISA, respectively, in FAEE-SAP and control mice (*n* = 4–6). (**C**) Relative transcription level of inflammatory genes *Il6*, *Mcp1*, and *Cxcl2* measured using qPCR in pancreata from FAEE-SAP and control mice (*n* = 4). (**D**) H&E, MPO, and TUNEL staining in AP and normal pancreas. Scale bar: 100 μm. (**E**–**G**) Quantification of necrotic area (**E**), MPO^+^ cells (**F**), and apoptotic cell area (**G**) (*n* = 4–6). *P* values calculated using unpaired Student’s *t* test. *P < 0.05, **P < 0.01, ***P < 0.001, n.s., not significant. Data are shown as mean ± SD. Each experiment was performed at least in triplicate.

To confirm the results of *Pfkfb3* inactivation from the iKO mice, we developed a pancreatic-specific KO of *Pfkfb3* driven by Cre expressed under the *pdx1* promoter (cKO). In the absence of AP, cKO mice have no macroscopic or microscopic differences from the WT animals (Supplemental Figure 3, H–K). Similar to the iKO animals, cKO mice had lower levels of serum amylase and MCP1 in the FAEE-SAP model of AP (Figure 3, A and B). *Il6* and *Cxcl2* expression were also lower in the pancreata of the cKO group (Figure 3C). The percentage of necrotic area, MPO^+^ cells and TUNEL^+^ area were reduced in cKO mice compared with WT mice (Figure 3, D–G, and Supplemental Figure 6, A–D). These findings support a protective role for *Pfkfb3* KO in the regulation of the pathogenesis of pancreatitis. Interestingly, this effect was observed in the FAEE-SAP model, thus defining a potentially new etiology-specific pathway driving AP.

### PFKFB3 modulates SAP progression independently of glycolysis.

**Figure 4:**
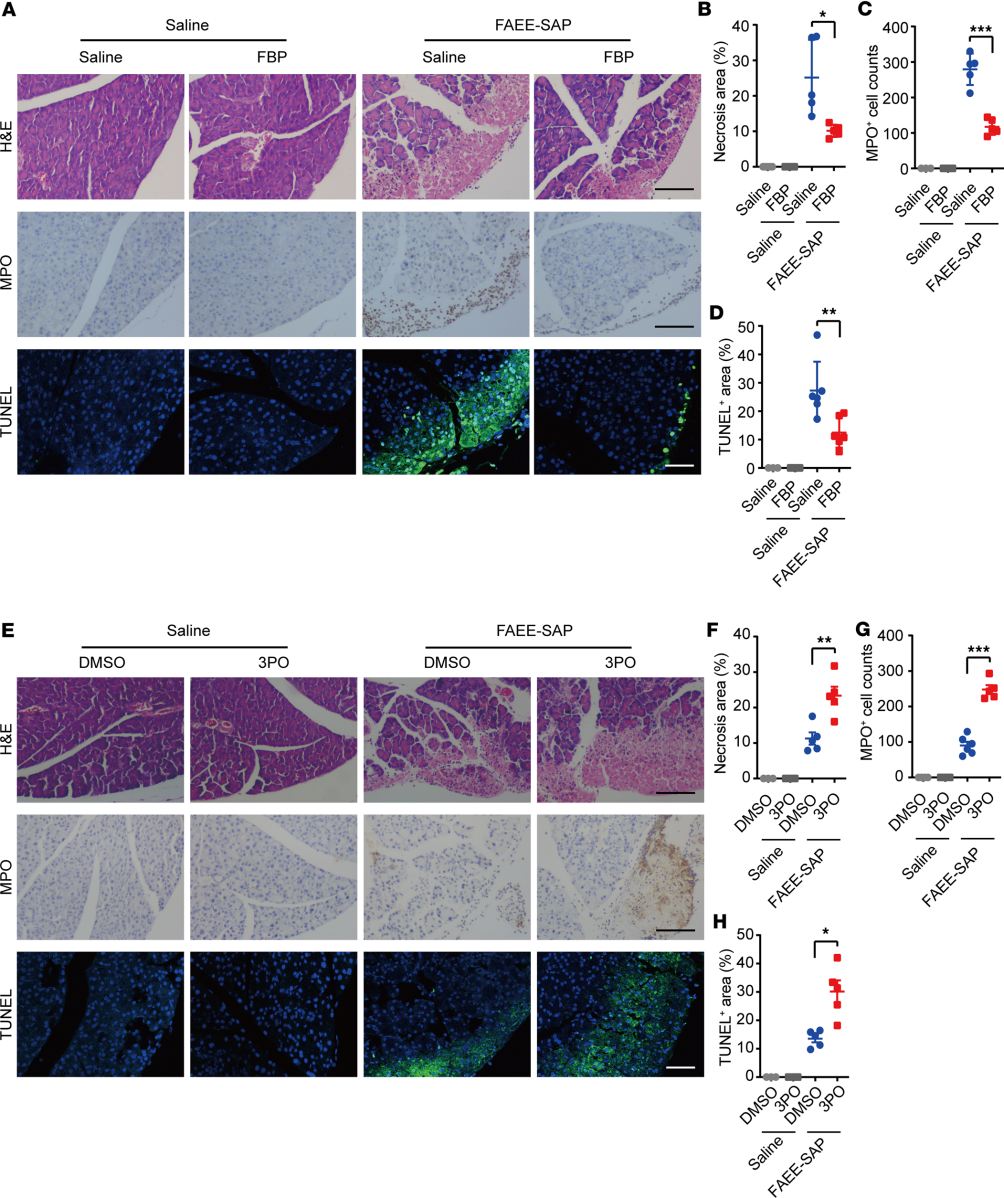
Exogenous supplement of fructose-1,6-bisphosphate (FBP) protects WT mice from FAEE-SAP, while inhibiting the glycolysis by 3PO aggravates the severity of FAEE-SAP. FBP was administered by i.p. injection at the dose of 500 mg/kg at 1 and 12 hours before AP induction. Mice were i.p. injected with 3PO (50 mg/kg) 1 hour before and 12 hours after AP induction. (**A**) H&E, MPO, and TUNEL staining in AP and normal pancreas. Scale bar: 100 μm. (**B**–**D**) Quantification of necrotic area (**B**), MPO^+^ cells (**C**), and apoptotic cell area (**D**) (*n* = 3–6). (**E**) H&E, MPO, and TUNEL staining in AP and control pancreas. Scale bar: 100 μm. (**F**–**H**) Quantification of necrotic area (**F**), MPO-positive cells (**G**), and apoptotic cell area (**H**) (*n* = 3–6). *P* values calculated using unpaired Student’s *t* test. *P < 0.05, **P < 0.01, ***P < 0.001. Data are shown as mean ± SD. Each experiment was performed at least in triplicate.

Next, we sought to determine if the effect of PFKFB3 in FAEE-SAP severity is mediated by dysregulation of the glycolytic pathway. To this end, we administered fructose-1,6-bisphosphate (FBP), the glycolytic intermediate immediately downstream of PFKFB3-mediated activation of PFK-1, to WT mice with FAEE-SAP to determine if enhanced glycolytic flux affects AP severity (Supplemental Figure 7A). Supplementation of FBP protected acinar cells from necrosis and reduced the infiltration of neutrophils and the number of TUNEL^+^ cells (Figure 4, A–D, Supplemental Figure 7, B–D, and Supplemental Figure 8A). Interestingly, FBP supplement also decreased the serum amylase and MCP1 in serum (Supplemental Figure 8, B and C), and pancreatic *Il6* expression (Supplemental Figure 8D).

To further test if the effects of *Pfkfb3* KO on AP severity in the FAEE-SAP model are mediated by alteration of glycolysis, mice were treated with i.p. injection of 3PO (21). 3PO inhibits glycolysis by competing with F-6-P instead of binding with PFKFB3 (22–24) (Supplemental Figure 7A). Serum amylase was not different between experimental groups; however, the group treated with 3PO had higher serum lipase compared with DMSO-treated mice (Supplemental Figure 9, A and B). In mice pretreated with 3PO followed by POA and ethanol, MCP1 serum in protein and pancreatic tissue in mRNA were elevated compared with the DMSO pretreatment group (Supplemental Figure 9, C and D). Also, 3PO-pretreated animals had increased acinar cell necrosis, neutrophil infiltration, and TUNEL^+^ cells (Figure 4, E–H; Supplemental Figure 9, E–G; and Supplemental Figure 10A).

Overall, exogenous supplement of FBP abrogated rather than aggravated the severity of AP, and glycolysis inhibition by 3PO did not phenocopy *Pfkfb3* genetic inactivation. Thus, allosteric modulation of glycolysis by PFKFB3 does not account for the reduced severity of AP observed in *Pfkfb3*-KO mice.

### PFKFB3 facilitates Ca^2+^ signaling activation in FAEE-SAP.

**Figure 5:**
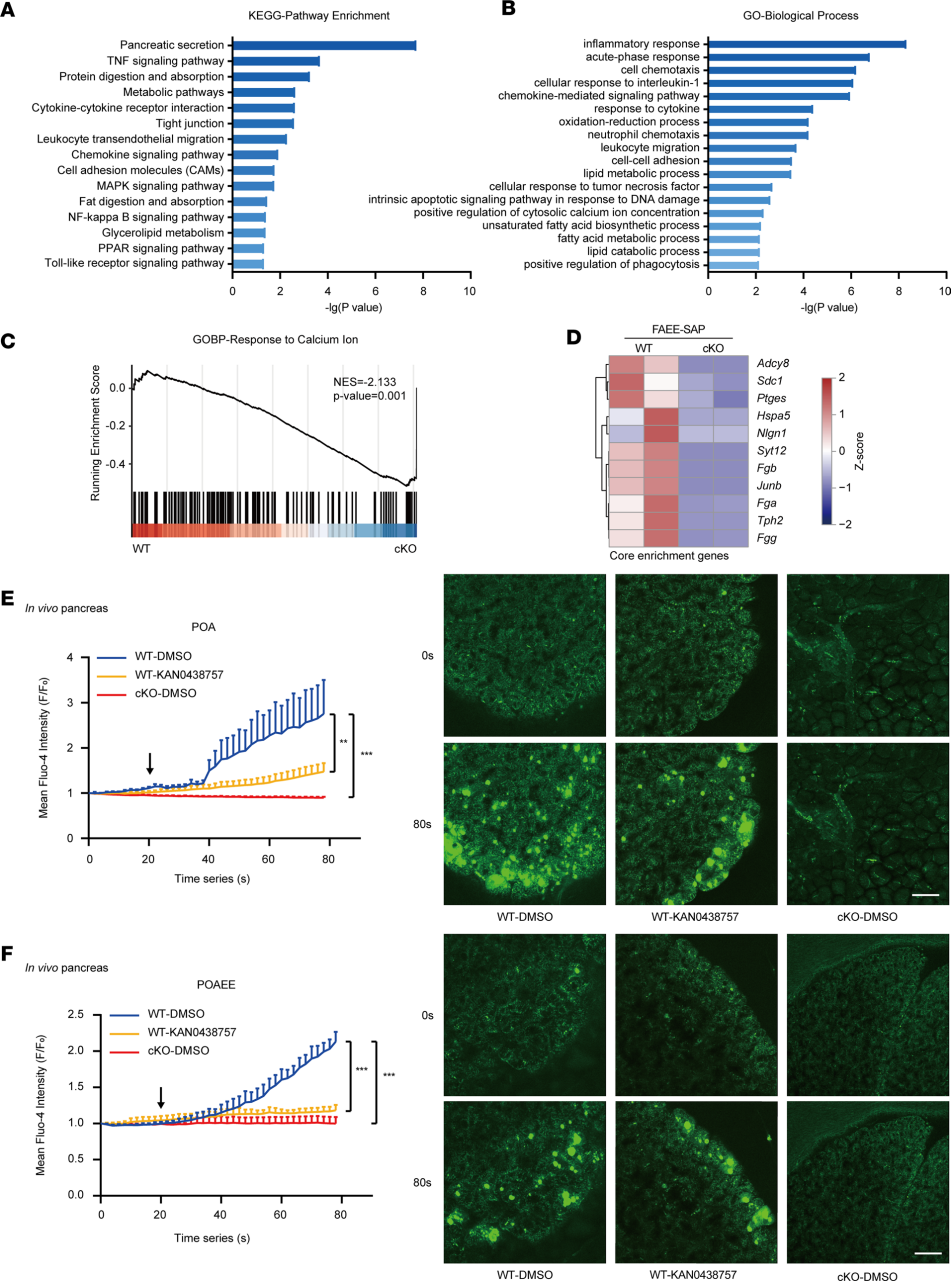
PFKFB3 is involved in the regulation of pathological calcium overload in acinar cells. (**A** and **B**) KEGG pathway enrichment (**A**) and GO analysis (**B**) in pancreas from cKO and WT mice from the FAEE-SAP model. (**C**) The enrichment plot comparing the transcriptome of cKO mice and WT mice in FAEE-SAP from GSEA analysis. Normalized Enrichment Score (NES), –2.133; normal *P* value, 0.001. (**D**) Heatmap shows the core enrichment genes in the pathway named response to calcium ion. (**E** and **F**) Representative traces of POA (**E**) or POAEE (**F**) induced Ca^2+^ elevations. The representative images are the first and last frames of pancreas prestained with Fluo-4. Scale bar: 40 μm. KAN0438757 (25 mg/kg) was administered by i.p. injection 1 hour prior to detection; DMSO in saline solution was used as a control. *P* values calculated using unpaired Student’s *t* test. *P < 0.05, **P < 0.01, ***P < 0.001. Data are shown as mean ± SD. Each experiment was performed at least in triplicate.

To define the mechanisms underlying the role *Pfkfb3* in AP pathogenesis, we performed RNA-Seq in WT and cKO mice treated with POA and ethanol. Supplemental Table 1 shows the differentially expressed genes (DEGs) in cKO mice. KEGG analysis revealed the presence of several pathways related with pancreatic function and inflammatory response, such as TNF signaling pathway and cytokine–cytokine receptor interaction (Figure 5A). Similarly, Gene Ontology (GO) analysis identified a number of biological processes including inflammatory response, acute-phase response, and cell chemotaxis (Figure 5B). Heatmaps also show genes participating in the pancreatic secretion, protein digestion and absorption, and inflammatory pathways (Supplemental Figure 11A). A subset of the DEGs were validated by qPCR (Supplemental Figure 11B).

All the aforementioned pathways are associated with calcium signaling, and this is particularly important as the dysregulation of intracellular calcium homeostasis is an early intraacinar event in AP (10, 25, 26). Further in silico analysis revealed that cKO mice have a transcriptional signature suggestive of reduced calcium signaling activity compared with WT mice (Figure 5, C and D). Furthermore, ultrafast 2-photon microscopy demonstrated that mice pretreated with KAN0438757, a PFKFB3 kinase inhibitor (24) (Supplemental Figure 11, C and D), had reduced intracellular free calcium following POA or palmitoleic acid ethyl ester (POAEE) treatment; cKO mice had an even larger decrease in calcium content (Figure 5, E and F, and Supplemental Videos 1–6). Together, these results support the dysregulation of calcium homeostasis as a central event mediating the effect of PFKFB3 in alcohol-related AP (Supplemental Figure 11E).

### PFKFB3 elevates intracellular Ca^2+^ level by interacting and activating IP3R.

**Figure 6:**
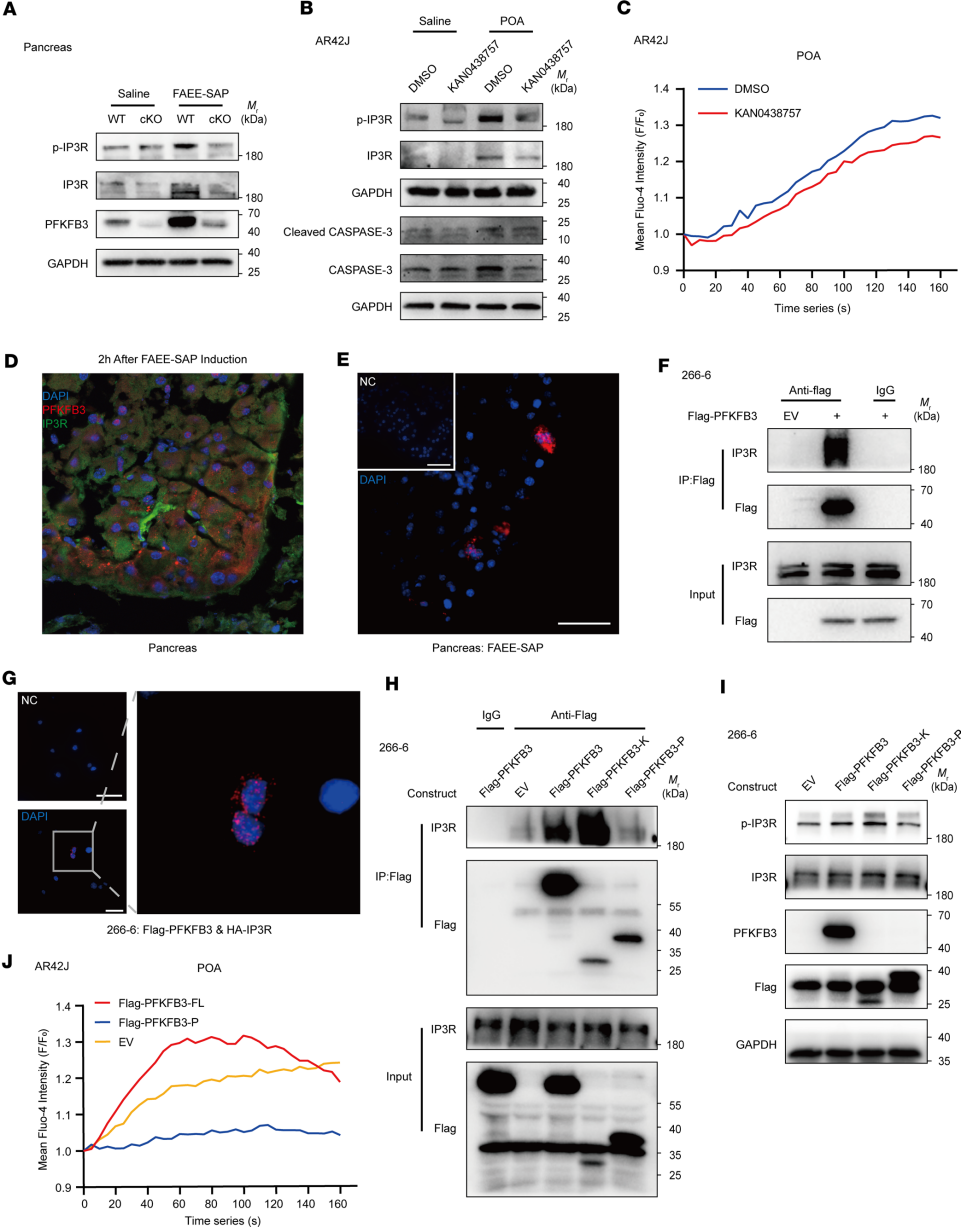
PFKFB3 cooperates with IP3R to regulate calcium homeostasis in pancreatic acinar cells. (**A**) Expression of IP3R, p-IP3R, PFKFB3, and GAPDH by Western blotting in mice pancreata from the FAEE-SAP model. (**B**) AR42J cells were pretreated with KAN0438757 (4 μM) or DMSO for 12 hours, and 0.5 or 6 hours after POA (300 μM) stimulation cells were harvested for Western blotting analysis. (**C**) Representative traces of POA induced Ca^2+^ elevations in AR42J cells were recorded by High Content Analysis Imaging System. KAN0438757 (4 μM) was administered 12 hours prior to detection; DMSO was used as a control. (**D**) Frozen section of pancreas damaged by FAEE (collected 2 hours after induction) evaluated by confocal microscopy. Scale bar: 40 μm. (**E**) PLA analyzed in frozen section of pancreas damaged by FAEE. Scale bar: 42 μm. (**F**) Whole-cell lysates of 266-6 cells transfected with Flag-PFKFB3 construct subjected to IP analysis and analyzed by Western blotting. (**G**) PLA was detected in 266-6 cells cotransfected with Flag-PFKFB3 construct and HA-IP3R construct. Scale bar: 50 μm. (**H**) 266-6 cells were transfected with full-length Flag-PFKFB3, Flag-PFKFB3-K (kinase domain only), and Flag-PFKFB3-P (phosphatase domain only) constructs for co-IP analysis. (**I**) 266-6 cells transfected with Flag-PFKFB3, Flag-PFKFB3-K, and Flag-PFKFB3-P constructs were analyzed by Western blotting analysis. (**J**) AR42J cells transfected with Flag-PFKFB3-K construct or Flag-PFKFB3-P construct; representative traces of POA induced Ca^2+^ elevations were recorded by High Content Analysis Imaging System. Each experiment was performed at least in triplicate.

Next, we sought to determine the mechanism by which PFKFB3 is involved in regulating cytosolic calcium flux. Gene set enrichment analysis (GSEA) of the RNA-Seq data derived from cKO and WT animals did not demonstrate enrichment of genes involved in calcium signaling pathways including mitochondrial calcium ion homeostasis, calcium ion export, voltage gated calcium channel, and ryanodine sensitive calcium release channel (Supplemental Figure 12, A–E). Moreover, POA treatment did not augment the physical interaction of PFKFB3 with RYR (Supplemental Figure 12, F and G). In contrast to RYR, expression of IP3R and p-IP3R was decreased in cKO mice with FAEE-SAP compared with WT (Figure 6A and Supplemental Figure 13, A–E), while there was no difference in transcription levels (Supplemental Figure 14A). In vitro, in POA-stimulated AR42J cells, inhibition of PFKFB3 reduced apoptosis, the expression and phosphorylation of IP3R (Figure 6B), and the global free calcium level (Figure 6C). Conversely, PFKFB3 overexpression in 266-6 cells, even at low doses, increased endogenous and exogenous expression of IP3R and its phosphorylation without altering IP3R transcription (Supplemental Figure 14, B–D). Next, we evaluated the interaction between PFKFB3 and IP3R by co-IP, proximity ligation assay (PLA), and IF assay. PFKFB3 and IP3R colocalized in acinar cells when FAEE-SAP was induced (Figure 6D). Interaction of PFKFB3 and IP3R was also observed in frozen sections of FAEE-damaged pancreas by PLA (Figure 6E and Supplemental Figure 14E). Furthermore, we demonstrated that IP3R coimmunoprecipitated with PFKFB3 in 266-6 cells and AR42J cells (Figure 6F and Supplemental Figure 14F). Moreover, the interaction of PFKFB3 and IP3R has also been confirmed by PLA in acinar cells overexpressing Flag-PFKFB3 and HA-IP3R (Figure 6G and Supplemental Figure 14G).

PFKFB3 has 2 functional domains, an N-terminal kinase domain and a C-terminal phosphatase domain (27). The N-terminal domain catalyzes the synthesis of F-2,6-BP using F-6-P and ATP as substrates, and the C-terminal domain is mainly responsible for dephosphorylation of F-2,6-BP (28) (Supplemental Figure 14H). Further study of the interaction of PFKFB3 with IP3R indicated that the kinase domain but not phosphatase domain binds to IP3R (Figure 6H). Functionally, overexpression of PFKFB3 kinase domain, even at low doses, was sufficient to increase the level of IP3R and p-IP3R in 266-6 cells and to elevate the global calcium level in stimulated AR42J cells, while the C-terminal domain did not affect IP3R expression, phosphorylation, or global calcium level (Figure 6, I and J, and Supplemental Figure 14I).

**Figure 7:**
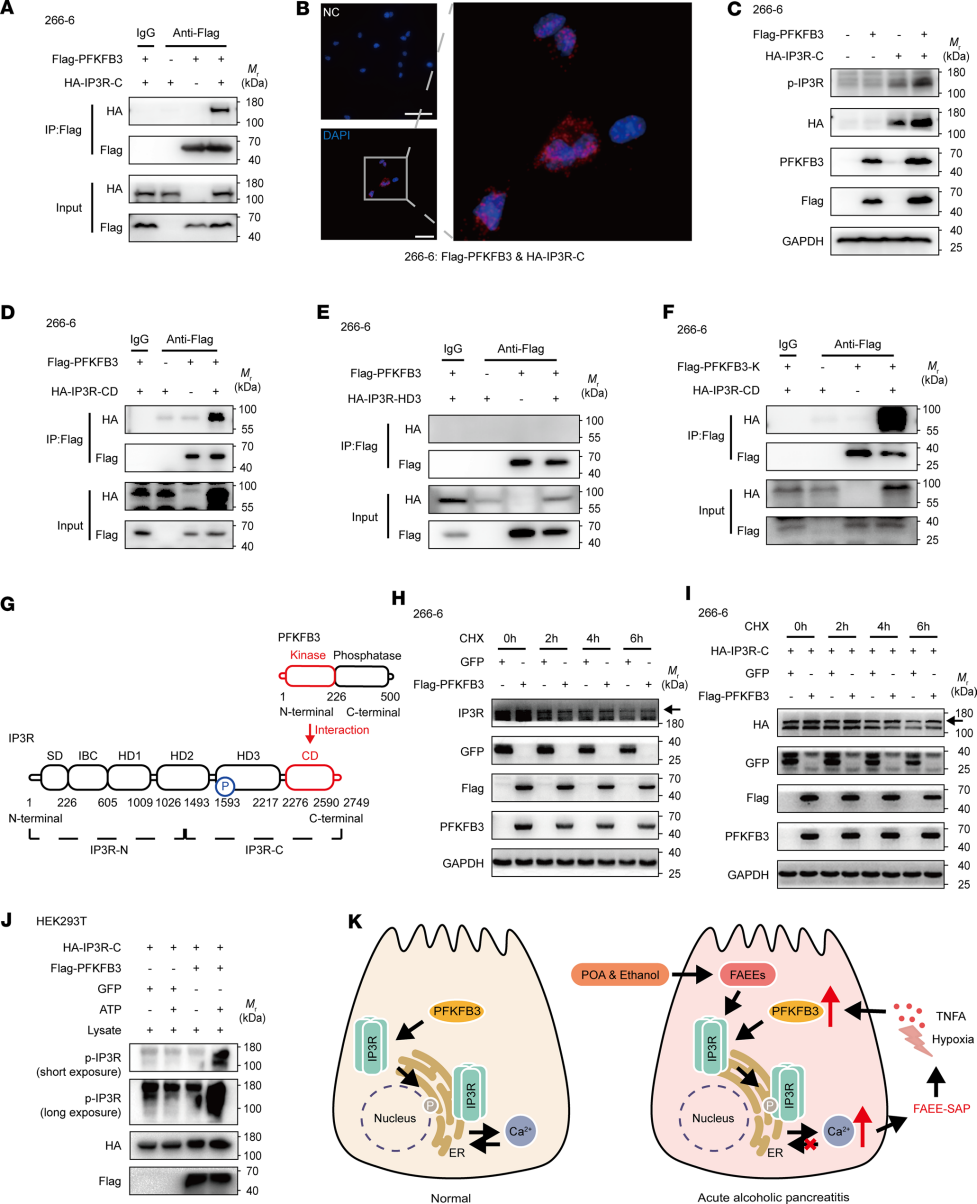
The kinase domain of PFKFB3 binds with the CD domain of IP3R and stabilizes IP3R expression. (**A**) Whole cell lysate of 266-6 cells transfected with Flag-PFKFB3 and HA-IP3R-C immunoprecipitated and analyzed by Western blotting. (**B**) PLA signal detected in 266-6 cells cotransfected with Flag-PFKFB3 construct and HA-IP3R-C construct. Scale bar: 50 μm. (**C**) Western blot in 266-6 cells were cotransfected with Flag-PFKFB3 and HA-IP3R-C constructs. (**D**) Whole cell lysates from 266-6 cells transfected with Flag-PFKFB3 and HA-IP3R-CD were used for co-IP. (**E**) Whole cell lysates were prepared from 266-6 cells were cotransfected with Flag-PFKFB3 and HA-IP3R-HD3 constructs were used for co-IP. (**F**) The Flag-PFKFB3-K and HA-IP3R-CD constructs were transfected in 266-6 cells for the co-IP analysis. (**G**) The schematic containing structural information of IP3R and binding sites of PFKFB3 and IP3R interaction. (**H** and **I**) Western blot of IP3R (**G**) and p-IP3R (**H**) in 266-6 cells cotransfected with Flag-PFKFB3 construct or GFP construct in the presence of cycloheximide (CHX, 100 μg/mL; 0, 2, 4, 6 hours). (**J**) Purified Flag-PFKFB3 protein, GFP protein, HA-IP3R-C, ATP, and 1× kinase reaction buffer were used for the in vitro kinase assay. (**K**) The working model of PFKFB3 in FAEE-SAP progression. Each experiment was performed at least in triplicate.

Similarly, IP3R contains 6 domains including suppressor domain (SD), IP3-binding core (IBC), α-helical domain 1 (HD1), α-helical domain 2 (HD2), α-helical domain 3 (HD3), and channel domain (CD) in sequence (29). Because the phosphorylation site of IP3R is in the c-terminus, we generated a tagged C-terminal construct of IP3R, designated IP3R-C, to study the domains of IP3R required for interaction with PFKFB3 (30). Co-IP assay and PLA confirmed the interaction between PFKFB3 and IP3R-C (Figure 7, A and B, and Supplemental Figure 14J), and PFKFB3 had the same functional effect on IP3R-C as on full-length IP3R (Figure 7C). Additional mapping results showed that IP3R CD and PFKFB3 kinase domain were sufficient to mediate the interaction of these molecules (Figure 7, D–G). In 266-6 cells cotransfected with plasmid Flag-PFKFB3 and control vector (GFP), a cycloheximide (CHX) pulse experiment demonstrated the stabilizing effect of PFKFB3 on IP3R and IP3R-C protein expression (Figure 7, H and I). Finally, in vitro kinase assays using purified PFKFB3 and IP3R-C showed that the purified kinase domain of PFKFB3 was sufficient for IP3R-C phosphorylation (Figure 7J and Supplemental Figure 14J).

In summary, our data show that KO of *Pfkfb3* abrogates tissue injury in alcohol-related AP by attenuating Ca^2+^ overload in pancreatic acinar cells. Mechanistically, PFKFB3 upregulates intracellular Ca^2+^ level by interacting with IP3R and promoting its phosphorylation and protein stability (Figure 7K).

## Discussion

AP is an inflammatory disease of the gastrointestinal tract with high morbidity and mortality. Due to the complexity of pathogenesis, the identification of therapeutic agents for AP has been challenging. Our data uncover a potentially new, glycolysis-independent role of PFKFB3 in controlling tissue injury in alcohol-related SAP. Furthermore, we defined an interaction between PFKFB3 and IP3R, a key early regulator of AP development and progression, and proposed that PFKFB3 promotes of severity of alcohol-related AP by upregulating intracellular calcium levels in an IP3R-dependent manner.

Under physiologic conditions, cytosolic Ca^2+^ concentration ([Ca^2+^]c), is maintained at low levels due to plasma membrane Ca^2+^ transport ATPase (PMCA), sarco/endoplasmic reticulum (SR/ER) Ca^2+^-ATPase (SERCA), and Na^+^/Ca^2+^ exchanger. In pancreatic acini, transient spikes in cytosolic calcium initiate zymogen exocytosis and maintain mitochondrial function (31). Sustained elevations of calcium under pathological stimuli, such as cholecystokinin hyperstimulation (32), oxidative (33) and non-oxidative (8) metabolites of ethanol, and bile acids (26), can trigger AP. This pathological elevation of intracellular calcium in pancreatic acinar cells leads to opening of mitochondrial permeability transition pore (PTP) and subsequent loss of the mitochondrial membrane potential, resulting in ATP depletion (34). Mitigation of AP progression requires ATP-driven apoptosis to eliminate damaged cells (35). In the setting of AP-induced mitochondrial dysfunction, glycolysis is a protective factor in the progression of AP (15–17). In our study, the protective effect of FBP and the deleterious effect of 3PO reconfirmed the protective role of glycolysis in AP progression.

In alcohol-related AP, FAEE leads to the superabundant release of calcium from the ER lumen through IP3R channels. Subsequently, loss of ER calcium concentration stimulates an influx of extracellular calcium via calcium release–activated calcium channel protein 1 (ORAI1) (9, 10, 36, 37). Here we demonstrate that the calcium source in FAEE-SAP is mediated by PFKFB3 in an IP3R-dependent manner. In gallstone pancreatitis, or in the NaTc-SAP model, calcium overload is the combined result of extracellular calcium entry and efflux from ER (38–40), caused by activation of piezo type mechanosensitive ion channel component 1 (PIEZO1) mediated by transient elevations of pancreatic duct pressures (38, 41) and bile acid (42) concurrently. The relatively complex sources of calcium in NaTc-SAP model may dilute the role of PFKFB3 in regulation of cytoplasmic calcium such that loss of PFKFB3-regulated calcium is insufficient to abrogate tissue injury in iKO mice.

Our data show that the kinase domain of PFKFB3 binds to the CD domain of IP3R. Overexpression of PFKFB3 in acinar cells augments IP3R protein expression without influencing its transcription and promotes the phosphorylation of IP3R. Consistently, in cKO mice, KO of PFKFB3 diminished IP3R expression and phosphorylation. Moreover, the PFKFB3 and IP3R interaction may explain why homozygous KO of *Pfkfb3* is embryonic lethal. KO of *Pfkfb3* and its interaction with IP3R has the potential to disrupt the spatiotemporal Ca^2+^ signals responsible for multitude processes including embryonic pattern formation, cell differentiation and proliferation, and transcription factor activation (43). This is a supplement to the previous theory, but it needs to be further tested.

Technically, we used ultrafast 2-photon in vivo imaging to perform ultrafast deep synchronous fluorescence imaging to achieve in-situ dynamic calcium measurements in acinar cells. In situ monitoring avoids the overactivation of primary acinar cells during the separation process and the unstable signal acquisition resultant of unstable adhesions of isolated acinar cells. Critically, intraductal injection of Fluo-4 for calcium ion staining did not activate acinar cells or downstream calcium ion signaling pathways, such as PIEZO1 and Trypsinogen channel (Supplemental Figure 15, A and B), proving the feasibility and value of this method.

In summary, we demonstrate that the upregulation of PFKFB3 in AP progression and that KO of *Pfkfb3* reduced the severity of FAEE-SAP. Dysregulated calcium signaling is the nexus event for AP progression, and disruption of the PFKFB3-IP3R interaction resulted in a reduction in calcium signaling, which explains the protective effect of *Pfkfb3* KO in alcohol-related AP. Finally, our study highlights PFKFB3 as a promising therapeutic target in alcohol-related AP.

## Methods

### Sex as a biological variable.

In our disease models, sex is not considered a biological variable.

### Mice.

*Pfkfb3^fl/fl^* mice were generated at the Model Animal Research Center of Nanjing University. Briefly, 2 LoxP sequences were inserted into the introns between exon1 and exon4 of *Pfkfb3* using CRISPR/Cas9 technology. Cas9 mRNA and sgRNAs were transcribed in vitro and then microinjected into fertilized eggs of C57BL/6J mice with the donor vector. Fertilized eggs were transplanted to obtain positive F0 mice, which were confirmed by PCR and sequencing. Stable F1 generation mice were obtained by mating the positive F0 generation mice with C57BL/6J mice. Mating with Cre mice deletes the floxed region containing exon 2 and exon 3 of *Pfkfb3* and disrupts PFKFB3 function. CAAG-Cre-ER^TM^ and *Pdx1*-Cre mice were purchased from Cyagen Bioscience to generate iKO (*Pfkfb3^fl/fl^*; CAAG-Cre-ER^TM^) and cKO (*Pfkfb3^fl/fl^*; *Pdx1*-Cre) mice. CAAG-Cre-ER^TM^ recombinase was induced by daily i.p. injection of tamoxifen (50 mg/kg) dissolved in corn oil from day 28 to 37. The genotyping primers for KO mice are 5′-CAGTTCCTCCCTGCCTGATT-3′ (LoxP forward [LoxP-F]), 5′-TTGGGCTACGCATTTAGTTC-3′ (LoxP reverse [LoxP-R]), 5′-GTGCCTGGCTAGAGATCCT G-3′ (ER^TM^-Cre-F), 5′-AGAGACTTCAGGGTGCTGGA-3′ (ER^TM^-Cre-R), 5′-GCGGTCTGGCAGTAAAAACTATC-3′ (*Pdx1*-Cre-F), and 5′-GTGAAACAGCATTGC TGTCACTT-3′ (*Pdx1*-Cre-R). The sizes of the PCR products are 360 bp (LoxP), 271bp (WT), 265 bp (ER^TM^-Cre), and 110 bp (*Pdx1*-Cre). WT mice used in this study were purchased from Vital River Laboratory Animal Technology. All the mice were maintained in a specific pathogen–free environment at Suzhou ISM under a controlled temperature (25°C) and a 12-hour light/dark cycle.

### Cell lines and reagents.

266-6 and HEK293T cell lines were purchased from American Type Culture Collection and cultured in DMEM supplemented with 10% FBS and 1% penicillin/streptomycin, at 37°C and 5% CO_2_. The AR42J cell line is a gift from Zhang Jinsan’s research team (Wenzhou Medical University) and cultured in DMEM/F12 basic medium supplemented with 20% FBS and 1% penicillin/streptomycin, at 37°C and 5% CO_2_. TNFA, 3PO, and KAN0438757 were purchased from MedChemExpress. CoCl_2_ was from Sigma-Aldrich. FBP was from Biovision, and CHX was from Maokang. Primary antibodies anti-PFKFB3 antibody (catalog ab181861), anti-Trypsinogen (catalog ab166898), and Anti-Ryanodine Receptor (catalog ab2868) were from Abcam. Primary antibodies anti-HA-Tag (catalog 3724S), anti-Caspase3 (catalog 14220S), anti–Cleaved Caspase3 (catalog 9661S), anti–p-IP3R (catalog 8548S), and anti-Α-tubulin (catalog 3873S) were from Cell Signaling Technology (CST). Anti-IP3R antibody (catalog sc-271197) was from Santa Cruz Biotechnology. Anti-PIEZO1 antibody (catalog A4340) was from Abclonal. Anti-GAPDH (catalog G9545) and HRP-conjugated anti-Flag (catalog A8592) antibodies were from Sigma-Aldrich. Secondary fluorescent antibodies anti–mouse IRDye800CW (catalog 926-32210), anti–rabbit IRDye800CW (catalog 926-32211), anti–mouse IRDye680RD (catalog 926-68070), and anti–rabbit IRDye680RD (catalog 926-68071) were purchased from LI-COR Biosciences.

### Plasmids and transfection.

Full-length and truncated *Pfkfb3* (gene ID, 170768) and *Ip3r* (gene ID, 16438) were amplified by PCR and cloned into pcDNA3.1 vector. Flag or HA tag was added into the N-terminal of the target genes. The indicated plasmids were transiently transfected into 266-6, HEK293T, or AR42J cells at 60%–70% confluence using Lipofectamine 3000 Transfection Reagent (Thermo Fisher Scientific). Master mix of Lipofectamine 3000 reagent and plasmids were prepared in Opti-MEM. Mix was incubated for 15 minutes at room temperature, before adding to cell cultures.

### AP and SAP mouse models.

Before induction of experimental AP or SAP, mice were fasted for 12 hours. Experimental CAE-AP mice were administered caerulein (MedChemExpress, 100 mg/kg) i.p. hourly for 10 hours as described previously (44, 45). Mice injected with saline were used as control groups. In experimental FAEE-SAP mice, the mixture of 150 mg/kg POA (MilliporeSigma) and 1.35 g/kg ethanol was injected i.p. hourly for 2 hours as described previously (8). Saline (200 μL) was injected i.p. immediately prior to POA and ethanol injections to avoid potential local damage by ethanol to peritoneal organs at the injection site, and 0.1 mg/kg buprenorphine hydrochloride was simultaneously given with the first injection of POA and ethanol for animal care. Experimental NaTc-SAP mice were induced by pancreatic ductal retrograde infusion of NaTc as previously described (40). Briefly, mice were anesthetized with pentobarbital, laparotomy was performed, and the duodenum was isolated and exposed such that the biliopancreatic duct could be clearly observed. A needle was passed through the duodenal wall directly opposite the papilla and placed within the duct. Once in position, the needle was fixed in place with a tied ligature. Retrograde infusion of 3% NaTc (2 μL/g, Solarbio) was performed at a flow rate of 10–12 μL/min. After infusion, the laparotomy was closed in 2 layers, and the mice were returned to the cage. Sham-operated animals underwent laparotomy and duodeno-pancreatic manipulation without infusion. All experimental mice were sacrificed 24 hours after AP induction, and the pancreatic tissue and blood specimen were collected. The pancreas was divided into 2 parts: one was preserved in 4% paraformaldehyde for pathological sections, and the other was snap-frozen in liquid nitrogen for RNA and protein extraction. Blood was kept at room temperature for 2 hours before being centrifuged (5,500*g*) for 15 minutes at 4°C to separate the serum. Serum was aliquoted to avoid repeated freeze-thaw cycles and stored in –20°C.

### Measurement of amylase, lipase, and MCP1 in serum.

All serum samples were properly diluted to ensure that the test values were within the detection range of the kit. Serum amylase was detected with α-Amylase Assay Kit (C016-1) purchased from Nanjing Jiancheng Bioengineering Institute. Diluted mouse serum samples were mixed with preheated substrate buffer, then reacted at 37°C for 7.5 minutes, and absorbance was measured at 660 nm after mixing with iodine solution. Serum lipase was detected with Lipase Activity Kit (A054-2) from Nanjing Jiancheng Bioengineering Institute. Diluted mouse serum samples were mixed with reagent 1, reacted at 37°C for 4 minutes, and reagent 2 was added and reacted for another 2 minutes. The absorbance was continuously measured for 2 minutes at 580 nm. The serum MCP1 was measured with a commercial ELISA kit from BioLegend. The capture antibody-coated 96-well plates were blocked with assay diluent, and the diluted serum samples were added and incubated at room temperature for 2 hours. Detection antibody was added to the capture antigen and then to the avidin-HRP solution; TMB substrate solution and stop solution were added successively for chromogenic reaction, measured absorbance at 450 nm. Absorbance values are converted according to standard curves or manufacturer’s recommendations.

### RNA extraction and qPCR.

For RNA extraction, snap-frozen pancreatic tissues were ground to a powder and were then transferred to tubes preloaded with TRIzol (Thermo Fisher Scientific). The RNA of cell lines was extracted using the Total RNA Rapid Extraction Kit (Fastagen). RNA was reverse-transcribed to cDNA using PrimeScript RT Reagent Kit (TaKaRa). qPCR was performed using TB Green Premix (TaKaRa). The expression levels of genes were normalized using the 2^–ΔΔCt^ method (46), using gene *Rpl32* or *B-Actin* as the internal control. All primers used in this study were designed by the PrimerBank (47), and all the primer sequences are available upon request.

### Protein extraction, IP, and immunoblot.

Snap-frozen pancreas tissues were ground to a powder and lysed with RIPA lysis buffer (Beyotime) containing complete protease inhibitor (Roche), PhosSTOP (Roche), and PMSF (Beyotime). Protein concentrations were measured by BCA assay (Beyotime). The co-IP lysis buffer (50 mM Tris-HCl, pH 7.5, 150 mM NaCl, 1 mM EDTA, 1% [v/v] Triton X-100) supplemented with complete protease inhibitor was prepared for cell lysis. Lysates of transfected 266-6 cell or AR42J cell were subjected to IP with Anti-Flag Sepharose Beads (MilliporeSigma), Anti-HA Sepharose Beads (MilliporeSigma), or Anti-IgG Sepharose Bead (CST) for 12 hours; lysates of cells transfected with empty vector were prepared as controls. For immunoblot, proteins were separated by 10% SDS-PAGE and transferred to PVDF membranes (MilliporeSigma). After incubating with the indicated primary and secondary antibodies, antigen-antibody complexes were visualized by chemiluminescence (ECL, MilliporeSigma) on Bio-Rad ChemiDoc XRS+ system or directly scanned by Odyssey CLx Imaging System (LI-COR). It should be noted that phosphorylated protein antibody was incubated on the same membrane after elution of total protein antibody. In addition, samples in Figure 6B were run at different times, and Figure 7C was set up in parallel and run contemporaneously.

### Histological examination, IHC, and IF.

Pancreatic tissues fixed in 4% paraformaldehyde for ≥ 36 hours were dehydrated and embedded in paraffin. Histologic sections were cut at 5 μm thickness. For H&E staining, after dewaxing and hydration, the histologic sections were stained with H&E and were finally dehydrated and sealed for observation. More than 5 fields views of each H&E-stained section were microscopically evaluated to quantify the degree of necrosis expressed as the percentage of analyzed pancreatic parenchymal area composed of necrosis. For IHC staining, after dewaxing and hydration, the histologic sections were treated with 0.01M sodium citrate buffer for antigen repair. After blocking, MPO antibody (ZSGB-BIO, PV-9001) or PFKFB3 antibody (Abcam, ab181861) and specific secondary antibody were incubated successively, followed by DAB staining and nuclear staining. Finally, the histologic sections were dehydrated and sealed for observation. The whit-point correction was done according to the blank background. To assess the degree of neutrophil infiltration, the number of MPO^+^ cells was counted using ImageJ (NIH) in several field views of MPO-stained sections. For TUNEL staining, after being dewaxed and hydrated, the proteins in histologic sections were dissolved in protease K solution, and the TUNEL test solution (Beyotime) was incubated for 1 hour under dark conditions. Finally we mounted coverslips using ProLong Diamond Antifade Mountant with DAPI (Invitrogen) and waited for observation. In the sections stained with TUNEL, the percentage of positive area relative to total field view area was calculated. All necrotic areas, MPO^+^ cells, and TUNEL^+^ areas were quantified by a reviewer masked to the treatment group.

For IF staining, pancreata were embedded in OCT compound (SAKURA); sections were cut at 8 μm thickness by cryotome. Frozen sections were blocked with 5% BSA and incubated overnight with the indicated antibodies. Stained sections were mounted with ProLong Diamond Antifade Mountant with DAPI (Invitrogen). PFKFB3 and IP3R signals were observed by SP8 LIGHTNING confocal microscope (Leica).

### PLA.

For PLA, frozen tissue sections or 266-6 cells in confocal dishes were fixed with 4% paraformaldehyde for 1 hour, and 0.2% Triton X-100 was used to permeabilize the cell membrane. Cells and tissues were incubated with blocking buffer (MilliporeSigma) to prevent nonspecific antibody binding. The primary antibody was added and incubated at 4°C overnight, and then the secondary antibody was incubated. After that, the ligation reaction and PCR were carried out, and finally we mounted coverslips using ProLong Diamond Antifade Mountant with DAPI (Invitrogen). Confocal microscopy was used to detect PLA signal.

### Ultrafast 2-photon in vivo imaging.

After WT mice were fasted for 12 hours, calcium ion probe Fluo-4 (30 μM, 2 μL/g, Thermo Fisher Scientific) was retrogradely infused into the pancreatic main duct 20 minutes before AP model induction. POA (10 μL, 300 mM) or POAEE (10 μL, 50 mM) (Cayman) was locally injected for 20 seconds for stimulation. Dynamic detection of calcium ions in pancreatic tissue was performed by Two-Photon Fluorescence Microscopy (OLYMPUS, FVMPE-RS) with a water dipping lens (OLYMPUS, N.A. 1.05, W.D. 2 mm). Femtosecond laser (SpectraPhysics) and barrier filter (BA495, 540 nm) were used for excitation and emission filtering, and the green fluorescence was collected using NDD detector. Images (512 × 512 pixels) were acquired at 2 frames/s using MicroManager (HIH). The mean fluorescence intensity (F) of 7 regions of interest (ROIs) at each time point was calculated by ImageJ software, and all fluorescence measurements were expressed as changes from basal fluorescence (F/F_0_ ratio), where F_0_ represents initial fluorescence at the beginning of each experiment.

### High-content analysis imaging system.

AR42J cells were pretreated with DMSO or KAN0438757 (4 μM) for 12 hours and were then incubated with calcium ion probe Fluo-4 (4 μM, Thermo Fisher Scientific) for 45 minutes. POA (300 mM) was used as a stimulant. Dynamic detection of calcium ions in AR42J cell line was performed by ImageXpress Micro Confocal (Molecular Devices). Images were acquired at 5 frames/s using a high-sensitivity high-resolution sCMOS camera (ANDOR, Zyla 4.2). The F of cells (*n* = 40–50) was calculated by ImageJ software, and all fluorescence measurements were expressed as changes from basal fluorescence (F/F_0_ ratio).

### RNA-Seq and analysis.

Total pancreatic RNA from experimental mice was extracted and reverse-transcribed to cDNA libraries for sequencing with NEBNext Ultra II Directional RNA Library Prep Kit (NEB). Libraries were sequenced on NovaSeq 6000 (Illumina). The pair-end sequencing data were treated by fastp software to filter low-quality reads, then mapped to Ensembl GRCm38.p6 reference genome by CLC genomics workbench 12.0 (Qiagen). DEGs were defined as genes for which fold change ≥ 2 or ≤ –2, with a *P* < 0.05 between the 2 groups. R package clusterProfiler and org.Mm.eg.db were the tools for GO and KEGG pathway enrichment. GSEA software (48, 49) was used to measure the enrichment score of all genes of whole transcriptomes of samples in gene sets.

### In vitro kinase assay.

PFKFB3 and IP3R-C were immunoprecipitated from the HEK293T cells transfected with Flag-PFKFB3 (10 μg) or HA-IP3R-C (10 μg) expression plasmids. After washing with PBS 3 times, cells were lysed, and PFKFB3 protein was eluted with 100 mg/mL Flag peptide (ChinaPeptides, MDYKDHDGDYKDHDIDYKDDDDK) and divided into 2 aliquots. IP3R-C protein immobilized on the agarose beads (MilliporeSigma) was divided into 4 aliquots. Then, the kinase assay was performed. According to manufacturer instructions, the mixture was incubated for 30 minutes at 27°C in 2.5× kinase reaction buffer — HEPES (62.5 mM), β-glycerol phosphate disodium salt pentahydrate (22 mM), MgCl_2_·6H_2_O (50 mM), MnCl_2_·4H_2_O (31.2 mM), EGTA (12.5 mM), EDTA (5 mM). Reactions were tested by WB. DNA sequences encoding GFP were cloned into a custom pET-based expression vector containing an N-terminal 6× His tag, and the protein was purified from *E*. *coli* BL21(DE3).

### Statistics.

Experiments were performed in at least triplicates. Data are shown as mean ± SD. All statistical analyses were performed with unpaired 2-tailed Student’s *t* test, log-rank (Mantel-Cox) test, or 1-way ANOVA (GraphPad Prism 8 software). *P* < 0.05 was considered as a statistically significant difference.

### Study approval.

Clinical specimens’ acquisition was approved by Medical Ethics Committee of The First Affiliated Hospital of Wenzhou Medical University (KY2022-R188). All animal experiments were conducted according to the *Guide for the Care and Use of Laboratory Animals* (National Academies Press, 2011) approved by the Animal Service Center of ISM (ISMIACUC-0009-R).

### Data availability.

Values for all data points for each graph are included in the Supporting Data Values file. The RNA-Seq data were deposited in GEO under the accession no. GSE203573. All data, reagents, and models are available upon request made to the corresponding author.

## Author contributions

FM and GC conceived the idea and designed the experiments. TZ, Shengchuan Chen, LL, SL, ZL, and FS performed all the experiments. YJ, LX, PG, AE, HY, BZ, and LW provided administrative, technical, or logistic support. CH, Siming Chen, YS, ZY, EJT, and MEFZ provided suggestions. JL designed and provided all the cKO mice. HS provided clinical specimens. TZ, FM, ACC, EJT, MEFZ, and GC analyzed the data and wrote the manuscript. The co–first authorship was based on the following contributions: TZ and Shengchuan Chen completed most of the experiments, and LL provided essential technical support and performed molecular biology experiments.

## Supplementary Material

Supplemental data

Unedited blot and gel images

Supplemental table 1

Supplemental video 1

Supplemental video 2

Supplemental video 3

Supplemental video 4

Supplemental video 5

Supplemental video 6

Supporting data values

## Version 1 05/23/2024

In-Press Preview

## Version 2 07/08/2024

Electronic publication
