# Supplementary material for: PFKFB3 controls acinar IP3R-mediated Ca2+ overload to regulate acute pancreatitis severity
Source: JCI Insight. 2024 May 23;9(13):e169481. doi: 10.1172/jci.insight.169481 (PMC11383365; doi:10.1172/jci.insight.169481)
Supplement: Supplemental data [file jciinsight-9-169481-s111.pdf]

## Supplementary materials

Tan Z. et al.Fig.S1

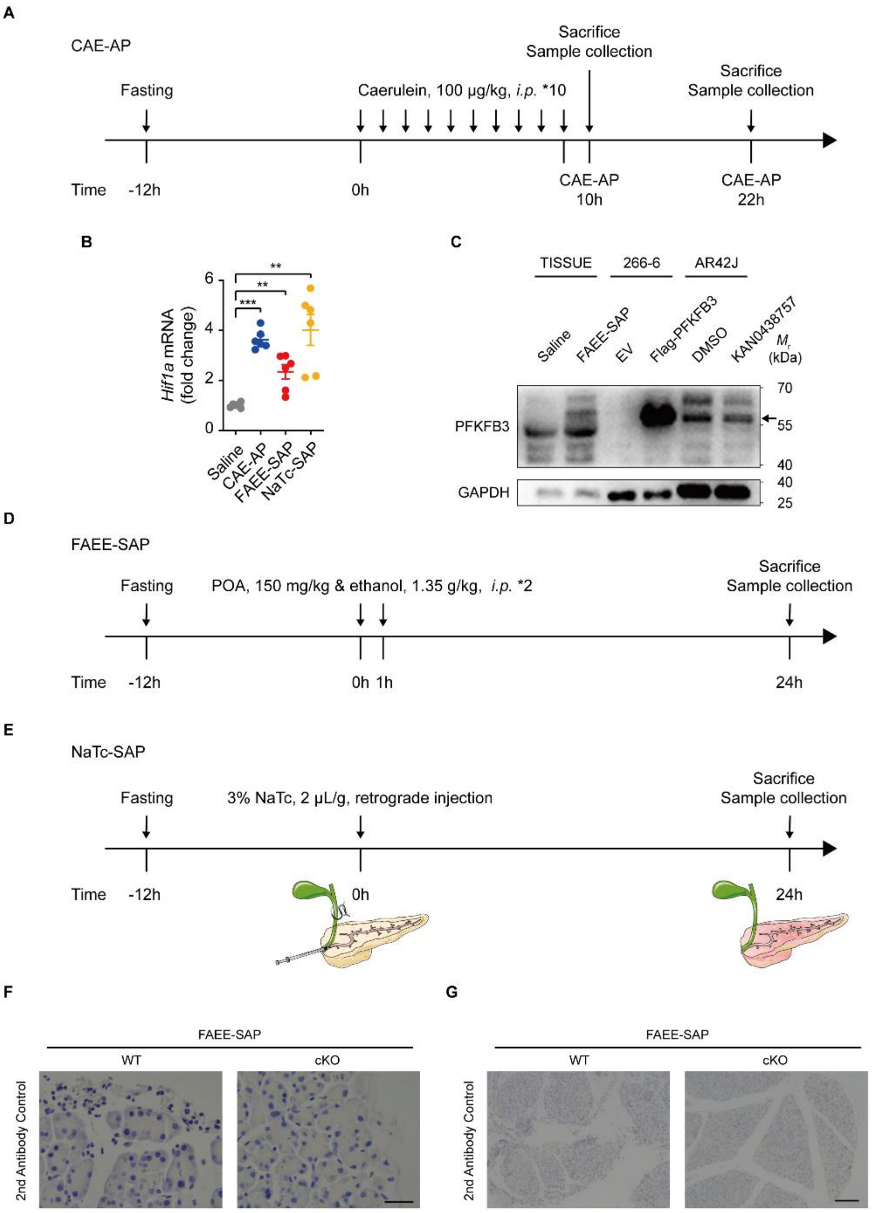


**Figure S1. Schematic diagram of the CAE-AP, FAEE-SAP and NaTc-SAP mouse model.** (**A**) Schematic diagram of the CAE-AP mouse. (**B**) The relative transcription level of *Hif1a* was measured using RT-qPCR in pancreas collected from CAE-AP, FAEE-SAP, NaTc-SAP and control mice (n=6). (**C**) The protein from pancreatic tissue, 266-6 cells and AR42J cells were collected for Western blotting analysis. (**D**) Schematic diagram of the FAEE-SAP mouse model. (**E**) Schematic diagram of the NaTc-SAP mouse model. (**F-G**) The secondary antibody control of PFKFB3 IHC analysis, scale bar, 30μm (**F**) and 200μm (**G**). Statistical analyses were done using one-way ANOVA test and shown as mean ± SD, $^{**}p<0.01$, $^{***}p<0.001$. Each experiment was done at least in triplicate.

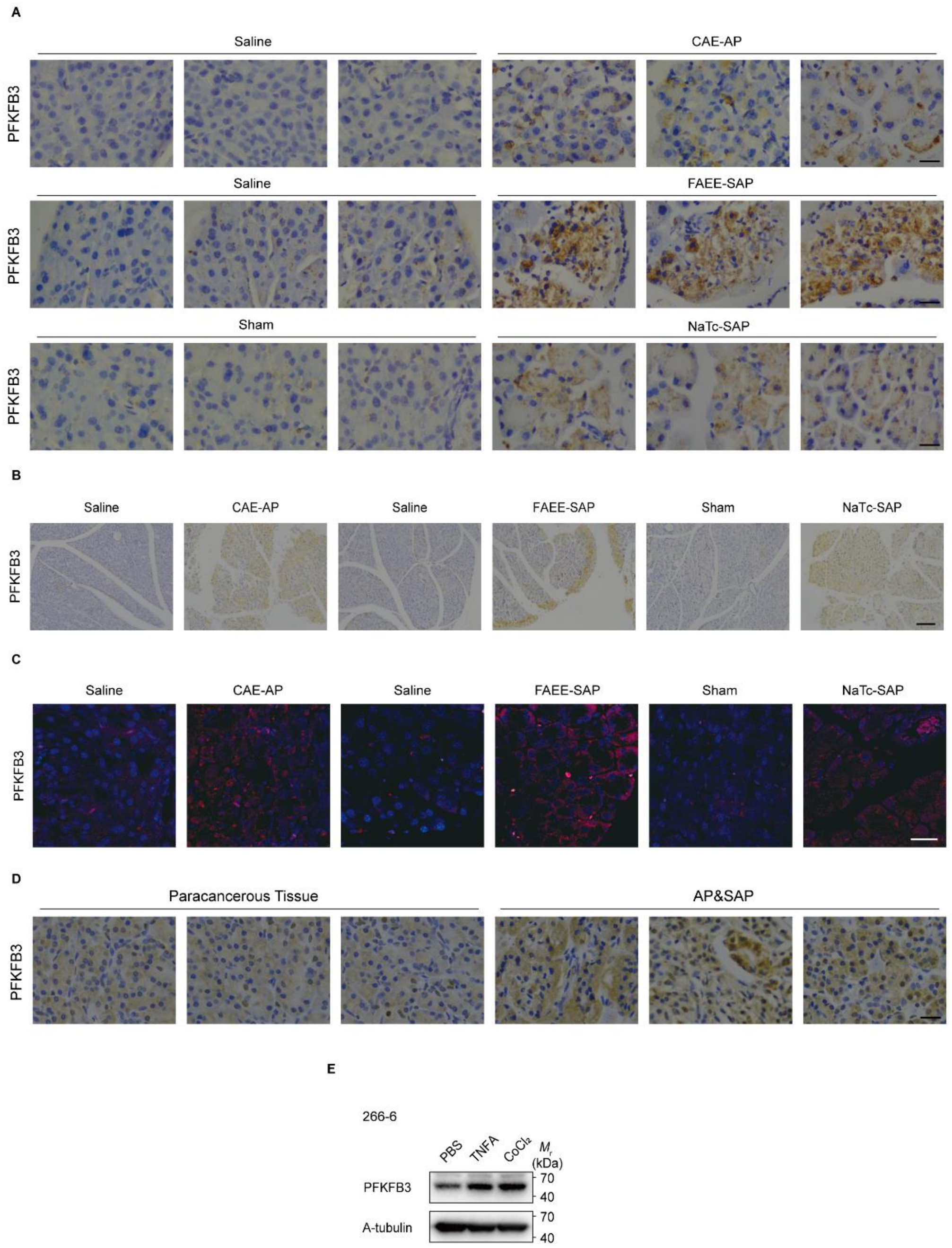


**Figure S2. PFKFB3 is upregulated of AP and SAP.** (**A**) Immunohistochemical analysis of PFKFB3 in pancreas from control group (n=5) and CAE-AP, FAEE-SAP and NaTc-SAP models (n=5), scale bar, 100μm. (**B**) Low magnification images of IHC stain in 3 AP models, scale bar, 200μm. (**C**) Immunofluorescence analysis of PFKFB3 in pancreas from control group and CAE-AP, FAEE-SAP and NaTc-SAP models, scale bar, 50μm. (**D**) Immunohistochemical analysis of PFKFB3 in pancreas from AP & SAP patient (n=5), scale bar, 40μm. (**E**) PFKFB3 levels were detected using Western blotting in 266-6 cells treated with TNFA (40ng/ml; 24h) and $CoCl_2$ (200μmol/l;24h). Each experiment was done at least in triplicate.

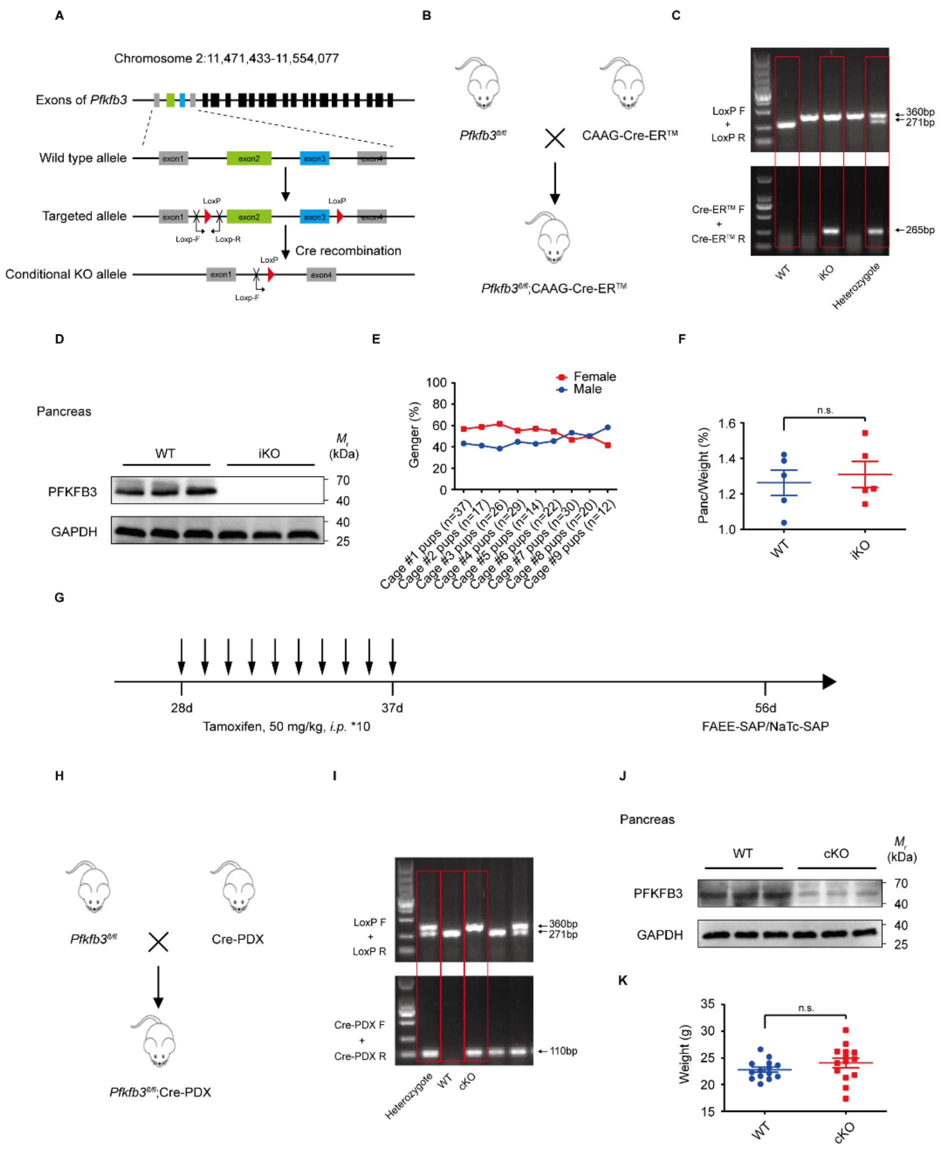


**Figure S3. Development of *Pfkfb3* inducible knockout (iKO) and conditional knockout (cKO) mice models.** (**A**) Strategy of Cre-LoxP recombination for the *Pfkfb3* knockout in C57BL/6cnc mice. (**B**) Breeding scheme of iKO mice. (**C**) Representative genotyping example of iKO mice. (**D**) The western blotting assay was used to verified the *Pfkfb3* knockout state in iKO mice in physiological status. (**E**) Gender distribution of iKO mice in multiple generations (n=9). (**F**) The ratio of pancreatic weight to body weight of iKO mice at 8 weeks compared to the WT counterpart (n=5). (**G**) Induction scheme for inducible *Pfkfb3* knockout. (**H**) Breeding scheme of cKO mice. (**I**) Representative genotyping example of cKO mice. (**J**) The western blotting assay was used to verified the *Pfkfb3* knockout state in cKO mice in physiological status. (**K**) Body weight in grams (g) of cKO mice at 8 weeks compared to the WT counterparts (n=14). Statistical analyses were done using unpaired student t-test and shown as mean ± SD, n.s., not significant.

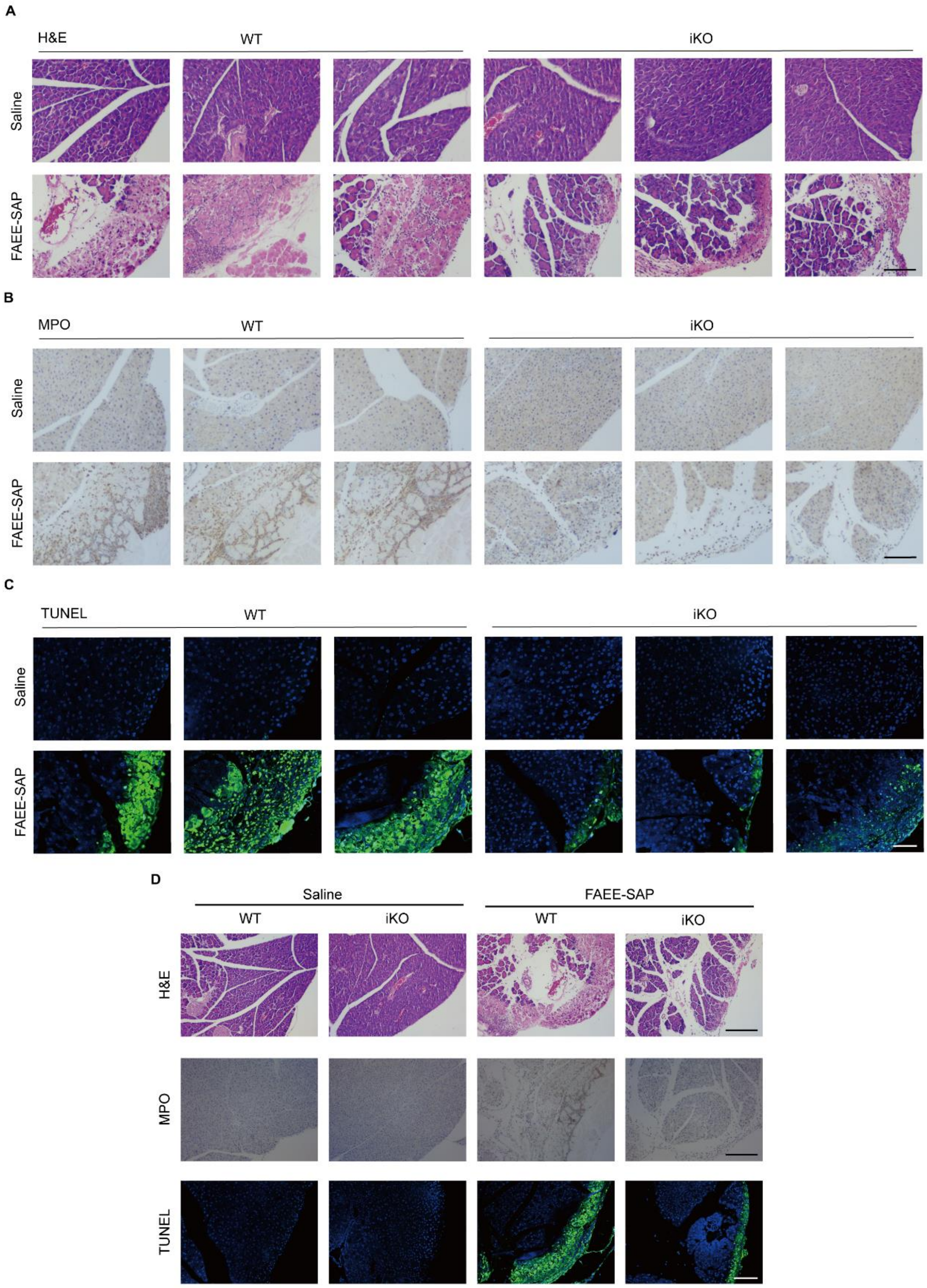


**Figure S4. H&E (A), MPO (B), and TUNEL (C) staining of injured and normal pancreas in WT mice and iKO mice.** Scale bar, 100μm. **(D) Low magnification images of H&E, MPO and TUNEL stains of injured and normal pancreas in WT mice and iKO mice.** Scale bar, 200μm.

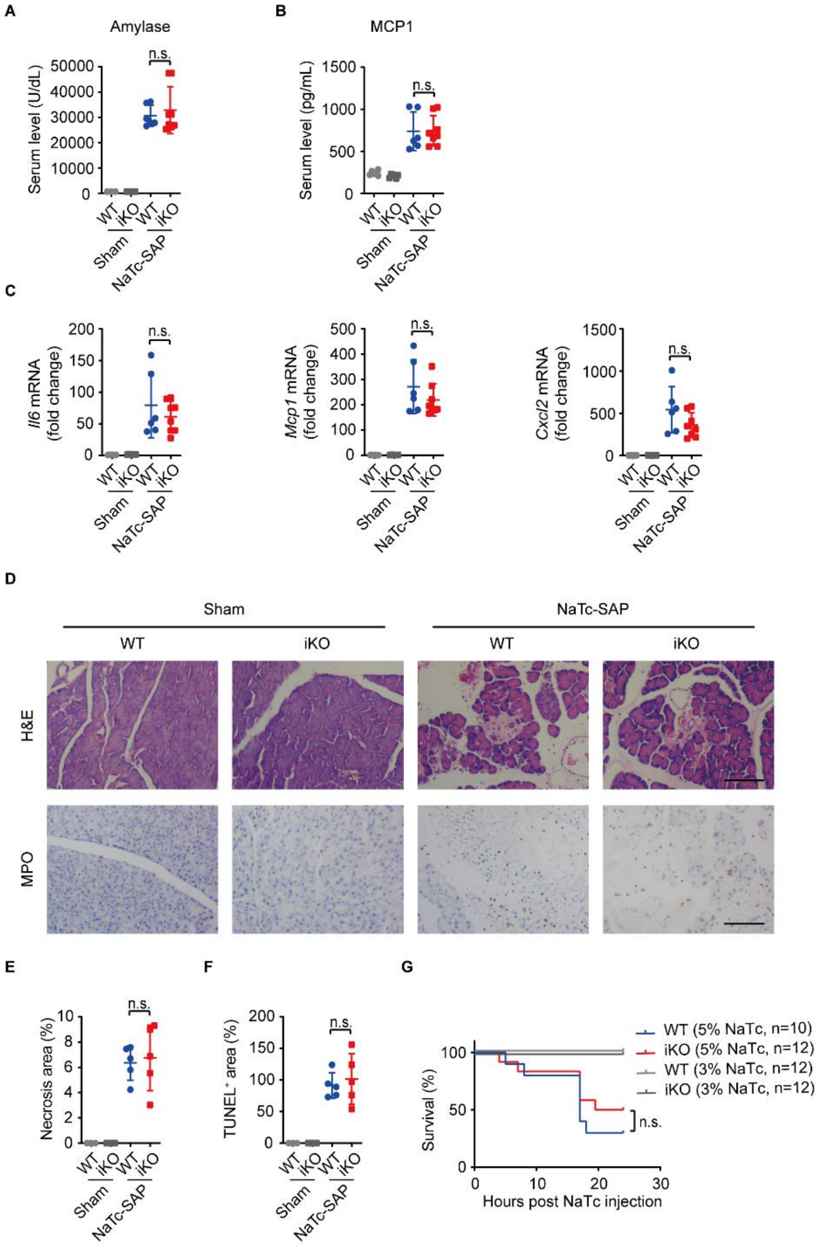


**Figure S5. The inducible knockout (iKO) mice do not affect pancreatitis severity in the NaTc-SAP model.** (**A-B**) Serum amylase (**A**) and MCP1 (**B**) levels were evaluated using Iodine-starch colorimetry and ELISA in iKO and WT control mice from the NaTc-SAP (n=3-8). (**C**) The relative mRNA expression of inflammatory genes, *Il6*, *Mcp1* and *Cxcl2* were measured using RT-qPCR in pancreas from iKO and WT control mice in the NaTc-SAP model (n=6-8). (**D**) H&E and MPO staining in injured and control pancreas; scale bar, 200μm. (**E-F**) Quantification of levels necrosis (**E**) and MPO (**F**) (n=3-5). (**G**) Twenty-four hours survival of mice from the NaTc-SAP model (n=10-12). The statistical analysis of the survival was done using log-rank (Mantel-Cox) test, and all other statistical analyses were unpaired student t-test and shown as mean ± SD, n.s., not significant. Each experiment was done at least in triplicate.

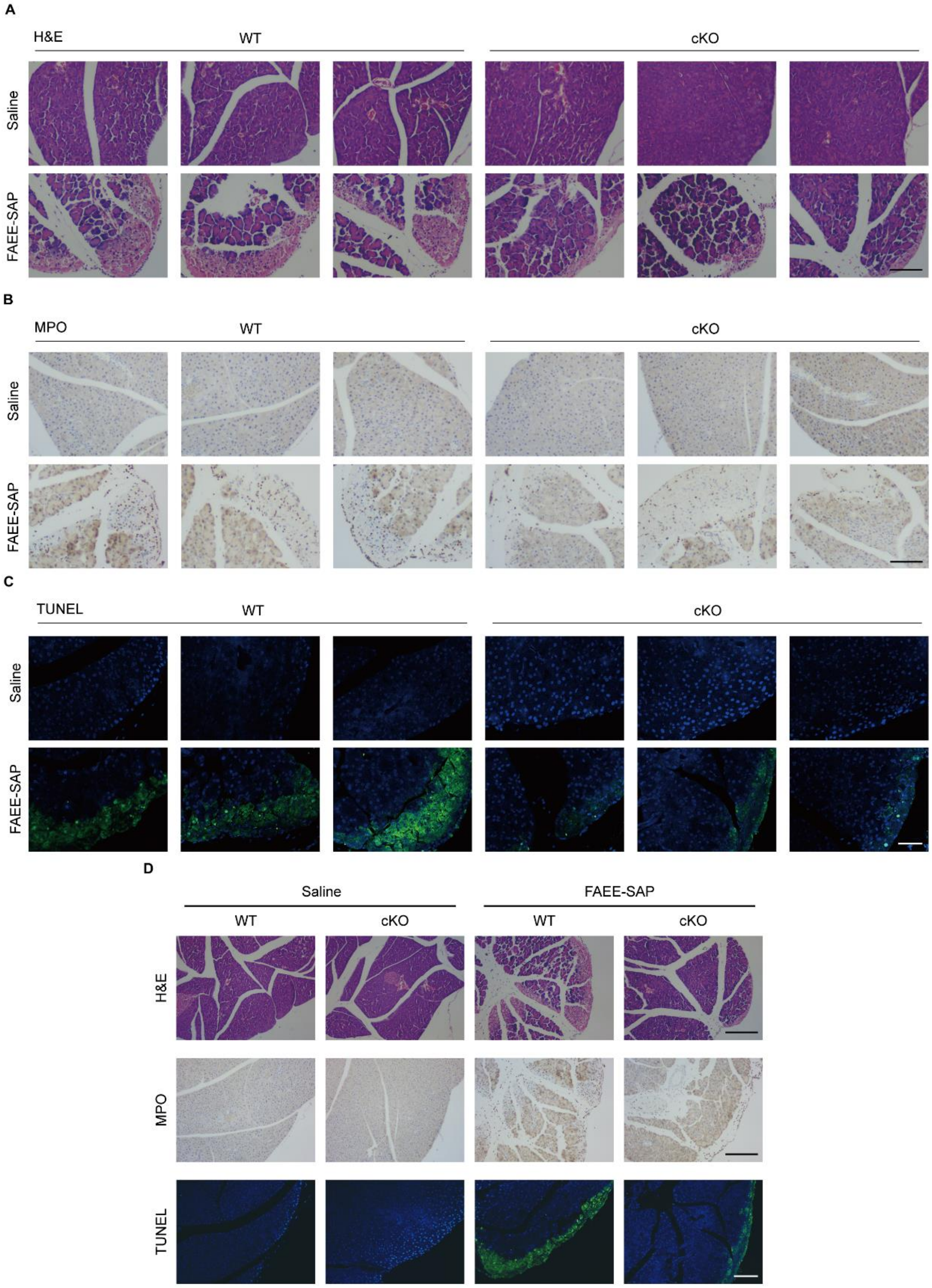


**Figure S6. H&E (A), MPO (B), and TUNEL (C) staining of injured and normal pancreas in WT mice and cKO mice.** Scale bar, 100μm. **(D) Low magnification images of H&E, MPO and TUNEL stains of injured and normal pancreas in WT mice and cKO mice.** Scale bar, 200μm.

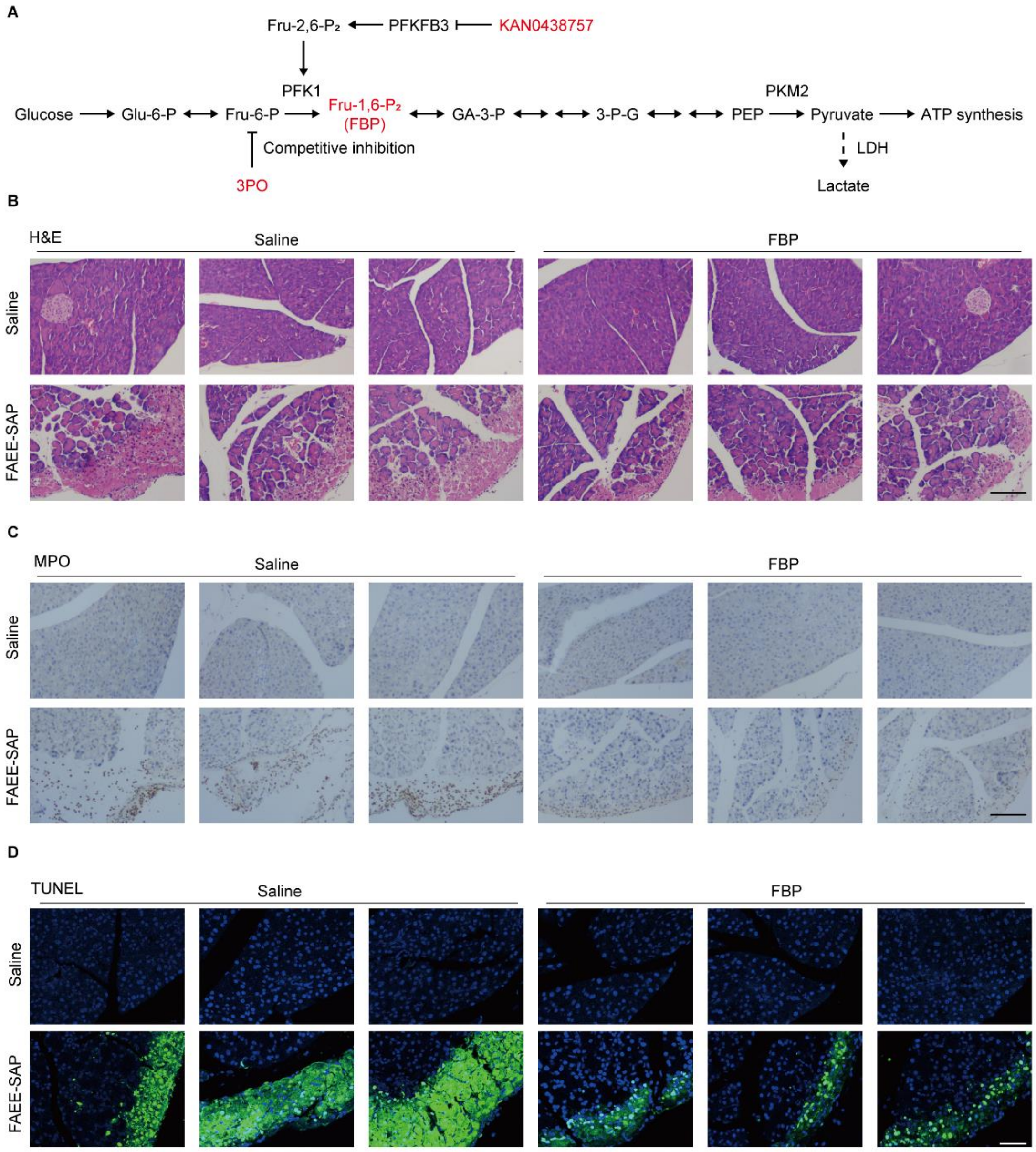


**Figure S7. FBP protect WT mice from FAEE-SAP.** (**A**) Working schematic of 3PO, FBP and KAN0438757 in glycolysis pathway. (**B-D**) H&E (**B**), MPO (**C**), and TUNEL (**D**) staining of injured and normal pancreas. Scale bar, 100μm. Each experiment was done at least in triplicate.

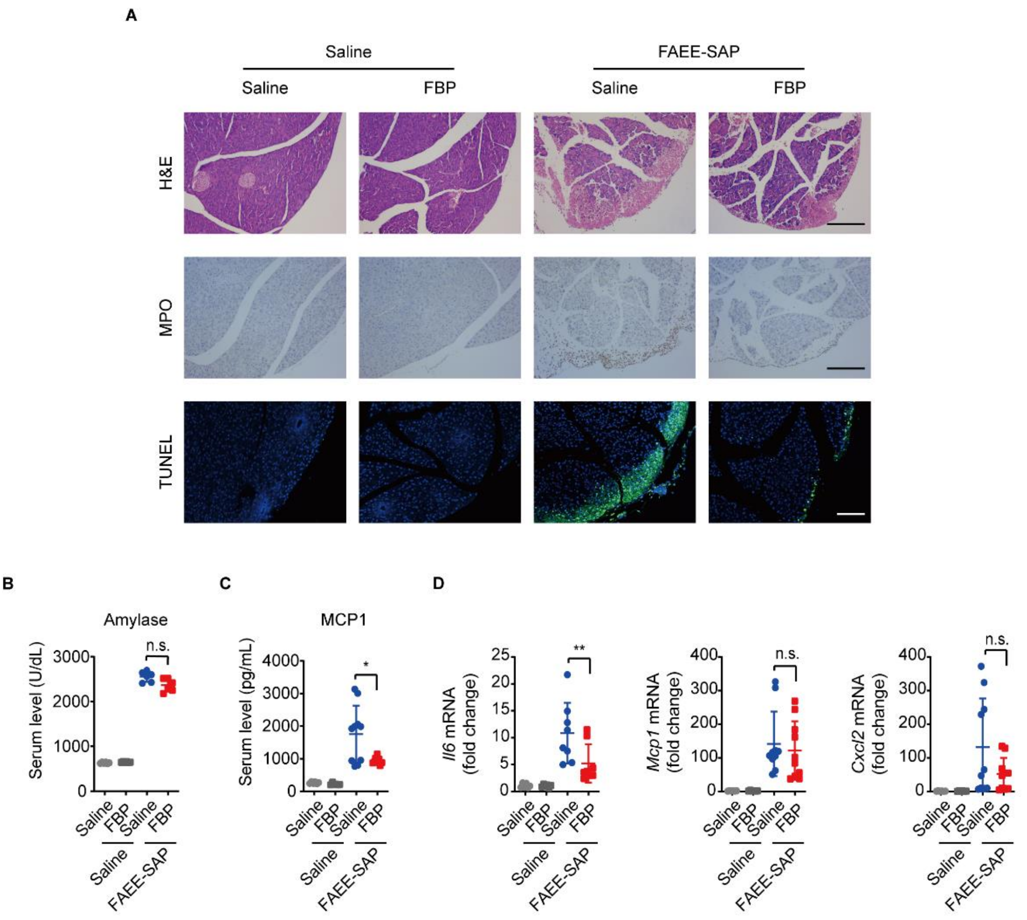


**Figure S8. FBP protect WT mice from FAEE-SAP.** (**A**) Low magnification images of H&E, MPO and TUNEL stains of injured and normal pancreas. Scale bar, 200μm. (**B-C**) Serum amylase (**B**) and MCP1 (**C**) levels were detected using Iodine-starch colorimetry and ELISA in FAEE-SAP and control mice (n=6-10). (**D**) *Il6*, *Mcp1* and *Cxcl2* mRNA expression were measured using RT-qPCR in pancreas collected from FAEE-SAP and control mice (n=6-10). Statistical analyses were done using unpaired student t-test and shown as mean ± SD, *p<0.05, **p<0.01, ***p<0.001. Each experiment was done at least in triplicate.

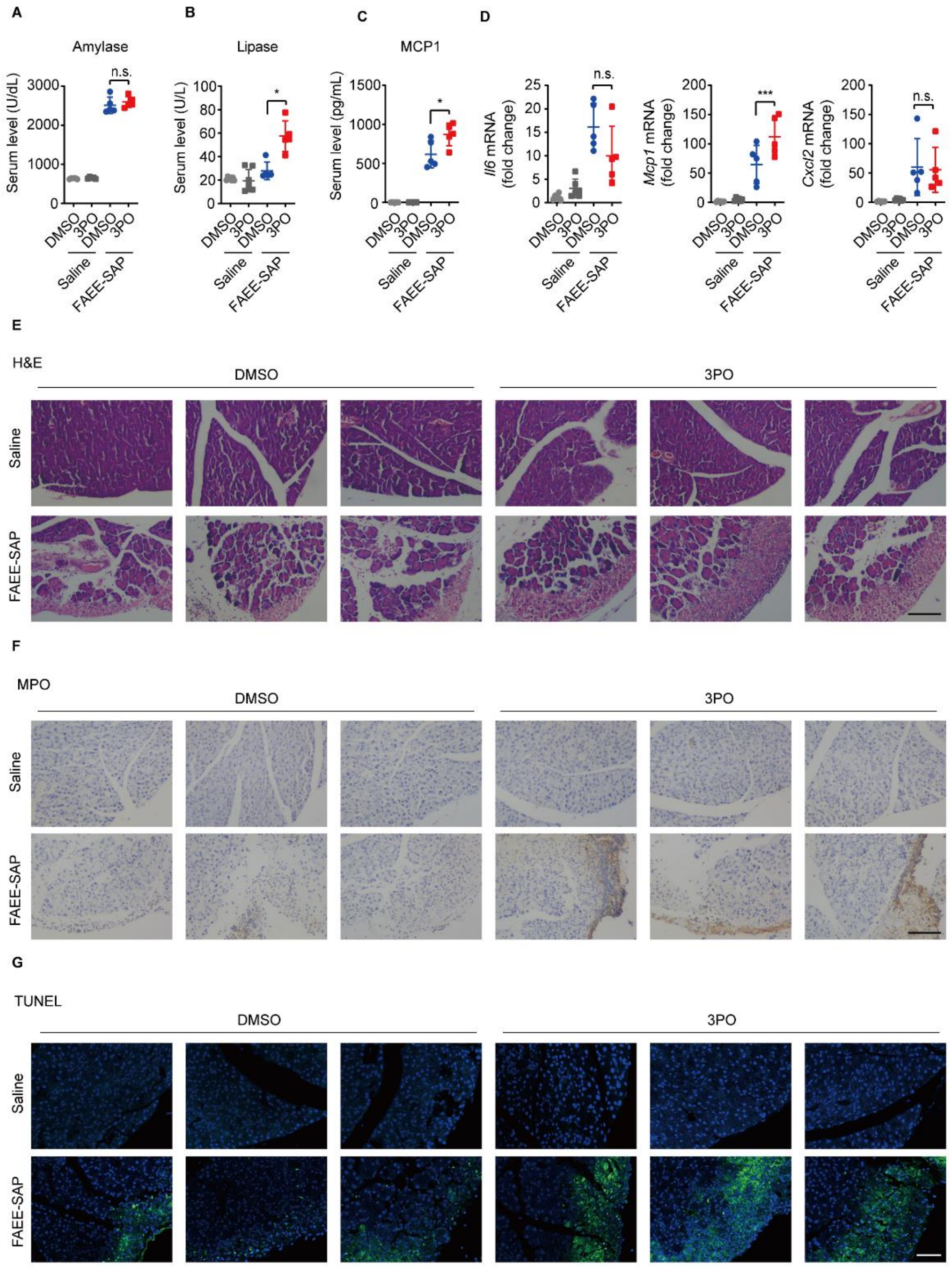


**Figure S9. 3PO aggravates the severity of FAEE-SAP.** (**A-C**) Serum amylase (**A**), lipase (**B**) and MCP1 (**C**) levels were detected using colorimetry and ELISA, respectively in FAEE-SAP and control mice (n=5-7). (**D**) Relative expression of inflammatory genes, *Il6*, *Mcp1* and *Cxcl2*, were measured using RT-qPCR in pancreas collected from FAEE-SAP and control mice (n=5-6). (**E-G**) H&E (**E**), MPO (**F**), and TUNEL (**G**) staining of injured and normal pancreas. Scale bar, 100μm. Statistical analyses were done using unpaired student t-test and shown as mean ± SD, *p<0.05, **p<0.01, ***p<0.001. Each experiment was done at least in triplicate.

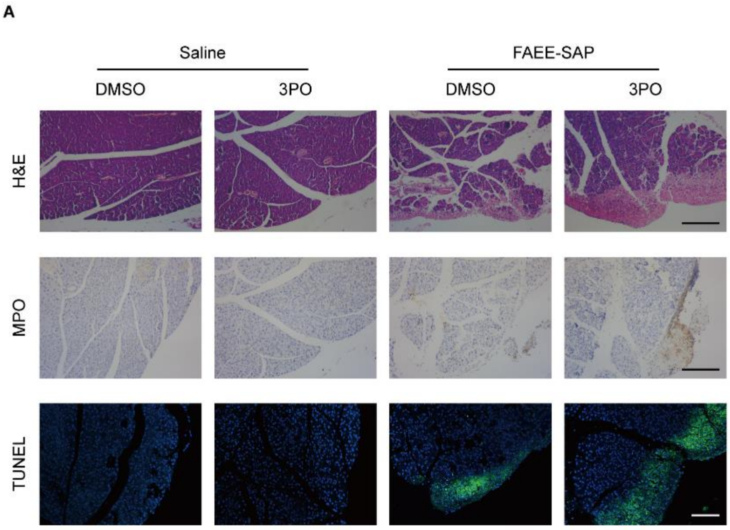


**Figure S10. 3PO aggravates the severity of FAEE-SAP.** (**A**) Low magnification images of H&E, MPO and TUNEL stains of injured and normal pancreas. Scale bar, 200μm.

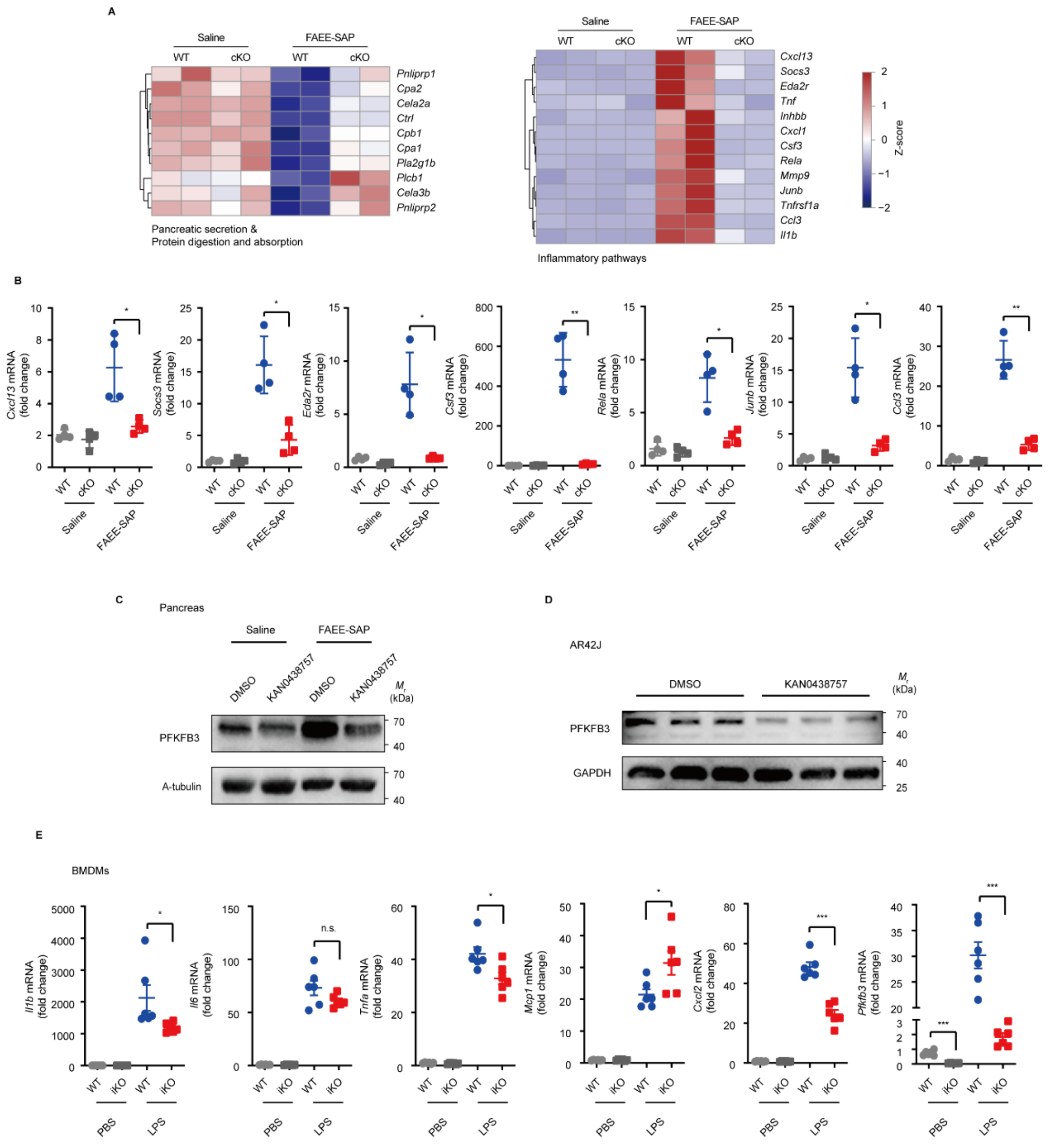


**Figure S11. Validation of sequencing data and protein level of pancreatic tissue. (A)** The heatmap shows the differentially expressed genes (DEGs) from **Fig. 5A** and **Fig. 5B**. FDR p<0.05 and fold change >2 or <-2. (**B**) The verification of representative differential expressed genes (DEGs) shown in **Fig.S11A** by RT-qPCR (n=4). (**C-D**) The western blotting assay was used to verified the Inhibitory effect of KAN0438757 on PFKFB3. (**E**) Expression level of inflammatory markers when stimulated with LPS (100ng/ml,6h) in BMDMs derived from iKO and WT mice were detected using RT-qPCR (n=6). All statistical analyses were done by unpaired student t-test and shown as mean ± SD, *p<0.05, **p<0.01, ***p<0.001, n.s., not significant. Each experiment was done at least in triplicate.

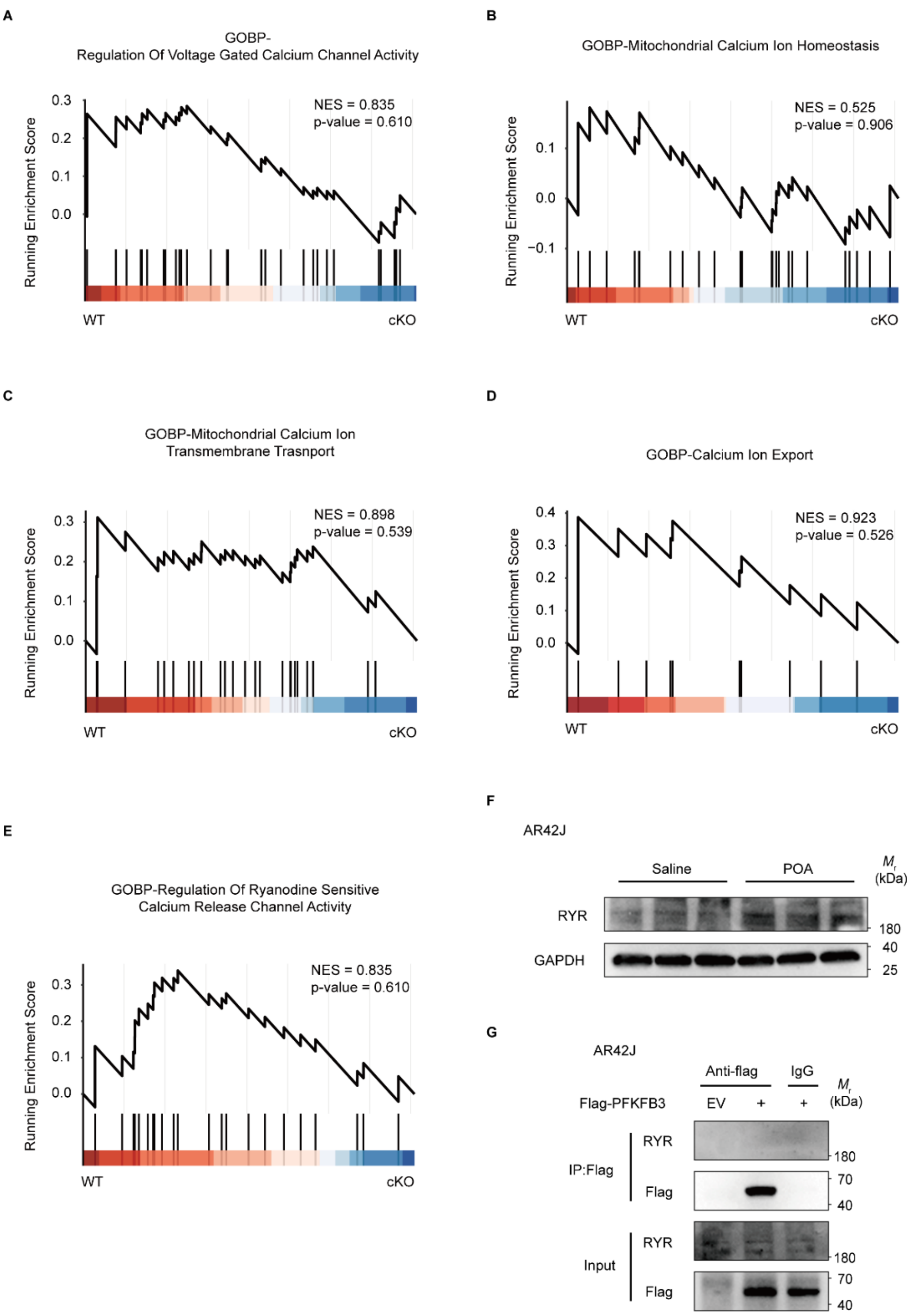


**Figure S12. Elimination of downstream targets of PFKFB3.** (**A-E**) The enrichment plot compared the transcriptome of cKO mice and WT mice in FAEE-SAP derived from the GSEA analysis. (**F**) RYR expression in AR42J cells stimulated with POA (300 μM) was determined by Western blotting. (**G**) AR42J cells were transfected with full length Flag-PFKFB3 construct for co-IP analysis.

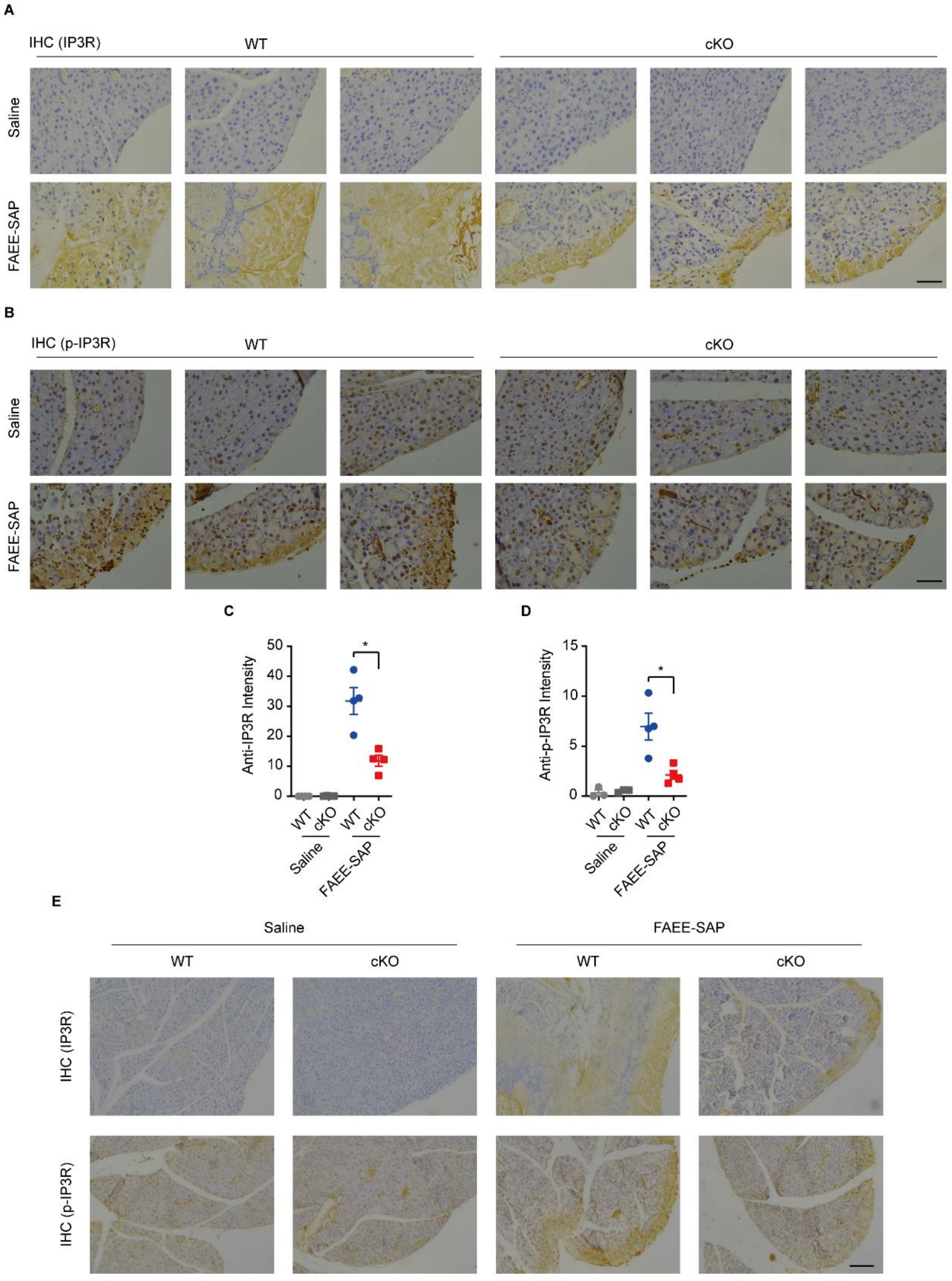


**Figure S13. Immunohistochemical analysis and quantification of IP3R (A, C) and p-IP3R (B, D) of injured and normal pancreas in WT mice and cKO mice.** Scale bar, 50μm. Low magnification images (**E**) of IHC stain of injured and normal pancreas in WT mice and cKO mice. Scale bar, 100μm.

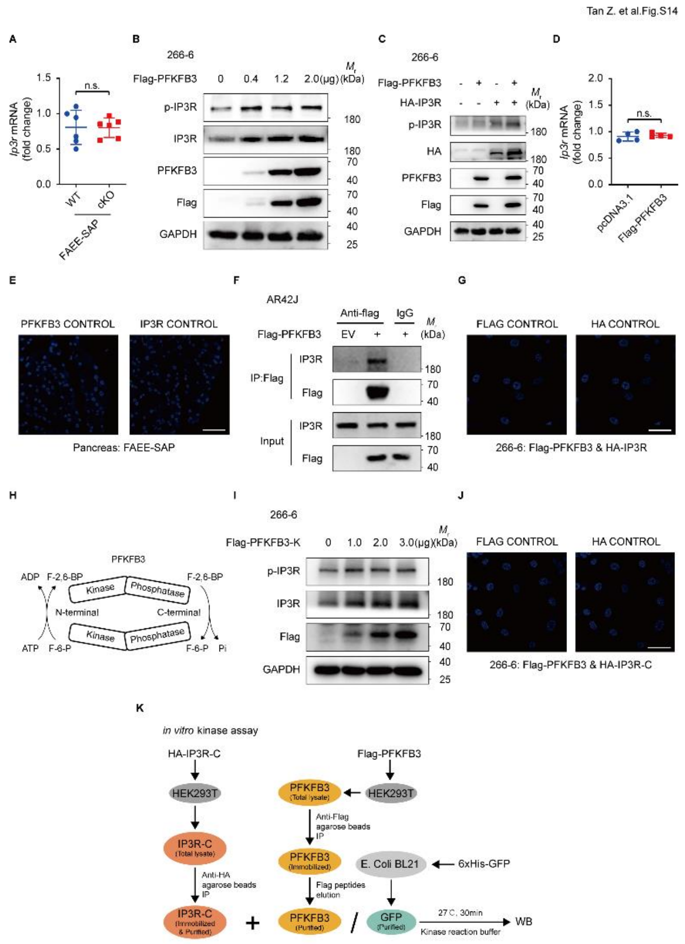


**Figure S14 Interaction between PFKFB3 and IP3R and its non-specific binding site.** (**A**) *Ip3r* mRNA expression of cKO mice and WT mice in the FAEE-SAP model (n=6). (**B**) 266-6 cells were transfected with increasing concentrations Flag-PFKFB3 construct, 24h post-transfection cells were harvested for Western blotting analysis. (**C**) Empty vector (pcDNA3.1), Flag-PFKFB3 and HA-IP3R constructs were co-transfected into 266-6 cells. After 24h, whole cell lysates were collected for Western blotting analysis. (**D**) mRNA expression of *Ip3r* in 266-6 cells transfected with empty vector (pcDNA3.1) and Flag-PFKFB3 constructs (n=4). (**E**) Antibody control of PLA assay in FAEE-SAP, scale bar, 40μm. (**F**) AR42J cells were transfected with full length Flag-PFKFB3 construct for co-IP analysis. (**G**) Antibody control of PLA assay in 266-6 cells co-transfected with Flag-PFKFB3 construct and HA-IP3R construct, scale bar, 40μm. (**H**) Schematic of the structural and functional information of PFKFB3. (**I**) Increasing concentrations Flag-PFKFB3-K were transfected into 266-6 cells for Western blotting analysis. (**J**) Antibody control of PLA assay in 266-6 cells co-transfected with Flag-PFKFB3 construct and HA-IP3R-C construct, scale bar, 40μm. (**K**) Schematic diagram indicates the principle of a cell-free system for *in vitro* kinase assay. All statistical analyses were done by unpaired student t-test and shown as mean ± SD, n.s., not significant. Each experiment was done at least in triplicate.

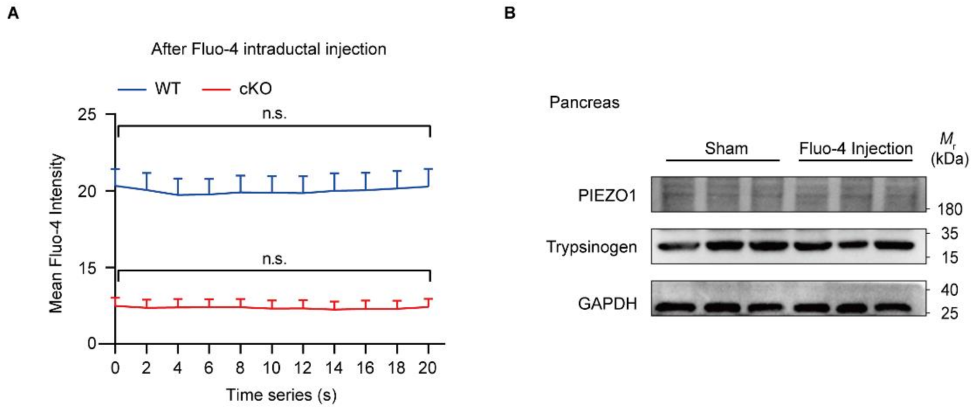


**Figure S15. (A) Representative traces of $Ca^{2+}$ in acini after Fluo-4 intraductal injection. (B) The Western blotting analysis for protein collected from pancreas which intraductal injected with Fluo-4 for 20 mins.** All statistical analyses were done by unpaired student t-test and shown as mean ± SD, n.s., not significant. Each experiment was done at least in triplicate.
