# Supplementary material for: PFKFB3 controls acinar IP3R-mediated Ca2+ overload to regulate acute pancreatitis severity
Source: JCI Insight. 2024 May 23;9(13):e169481. doi: 10.1172/jci.insight.169481 (PMC11383365; doi:10.1172/jci.insight.169481)

Full unedited gel for Figure.1B

Tan Z. et al.Fig.1

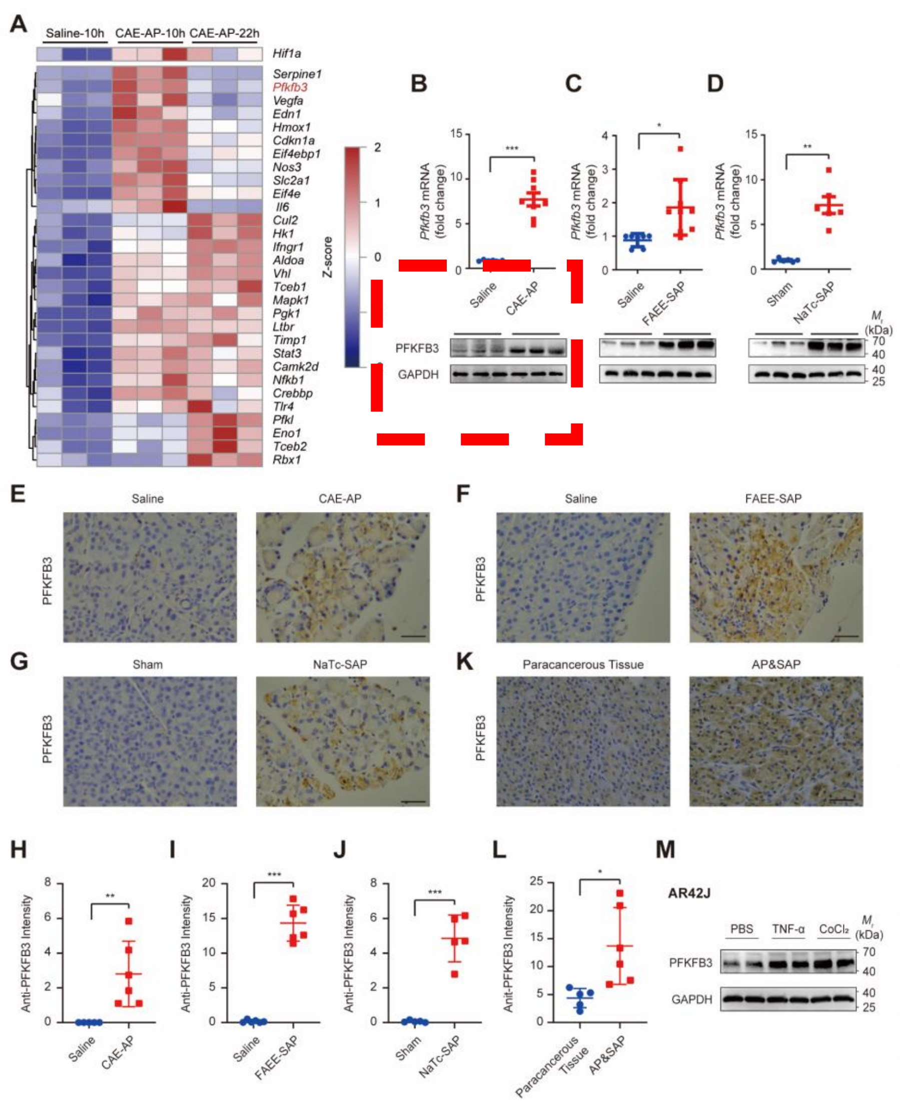

PFKFB3

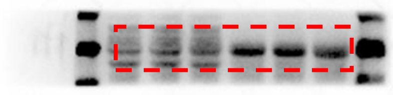

GAPDH

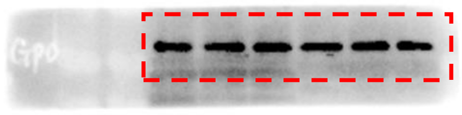

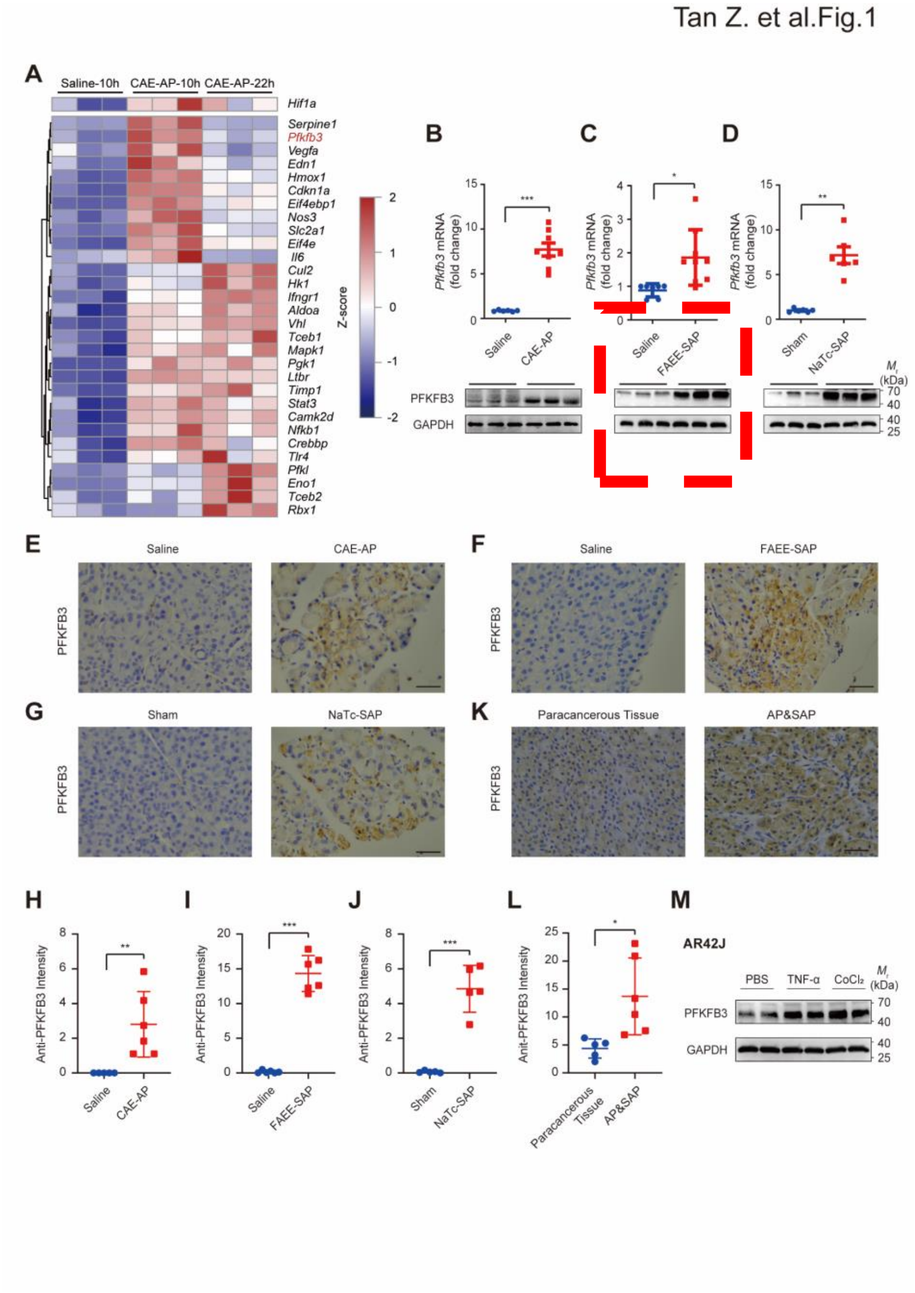

Full unedited gel for Figure.1C

PFKFB3

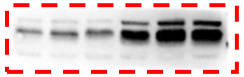

GAPDH

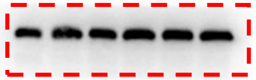

Full unedited gel for Figure.1D

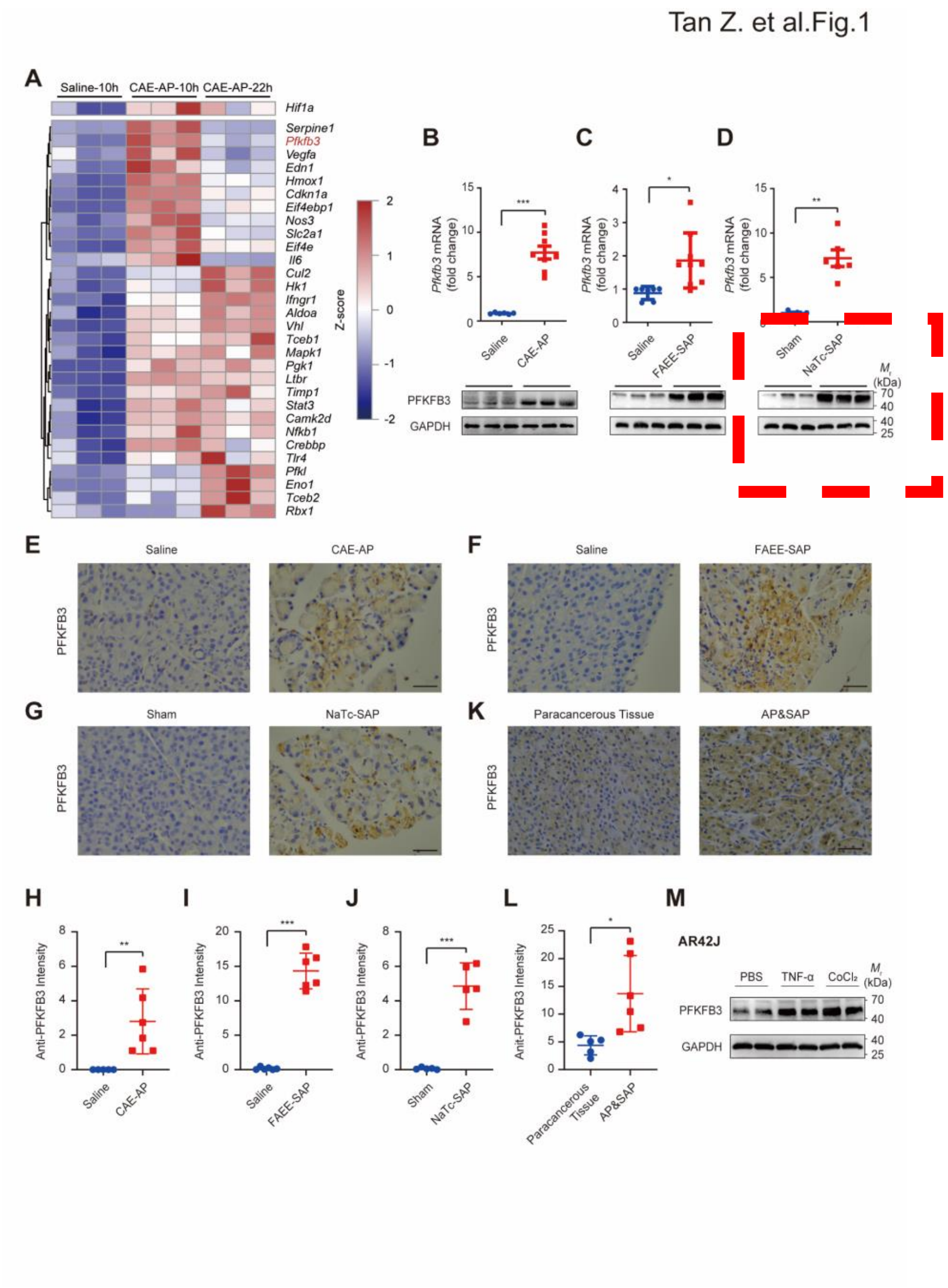

PFKFB3

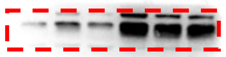

GAPDH

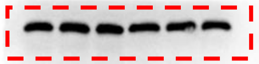

Full unedited gel for Figure.1M

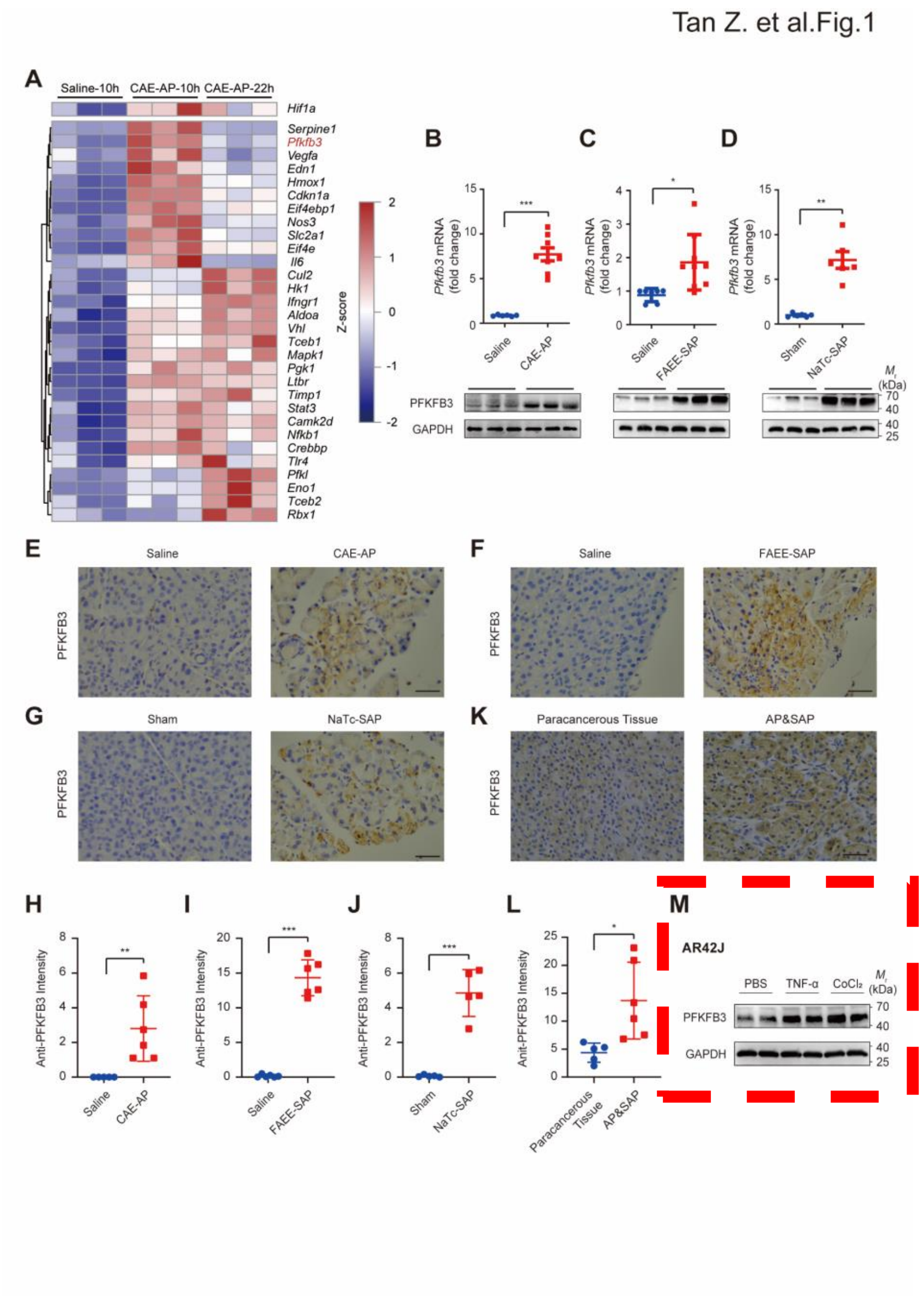

PFKFB3

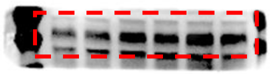

GAPDH

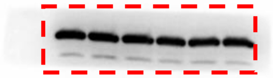

Full unedited gel for Figure.6A

Phosphorylated protein antibody was incubated on the same membrane after elution of total protein antibody.

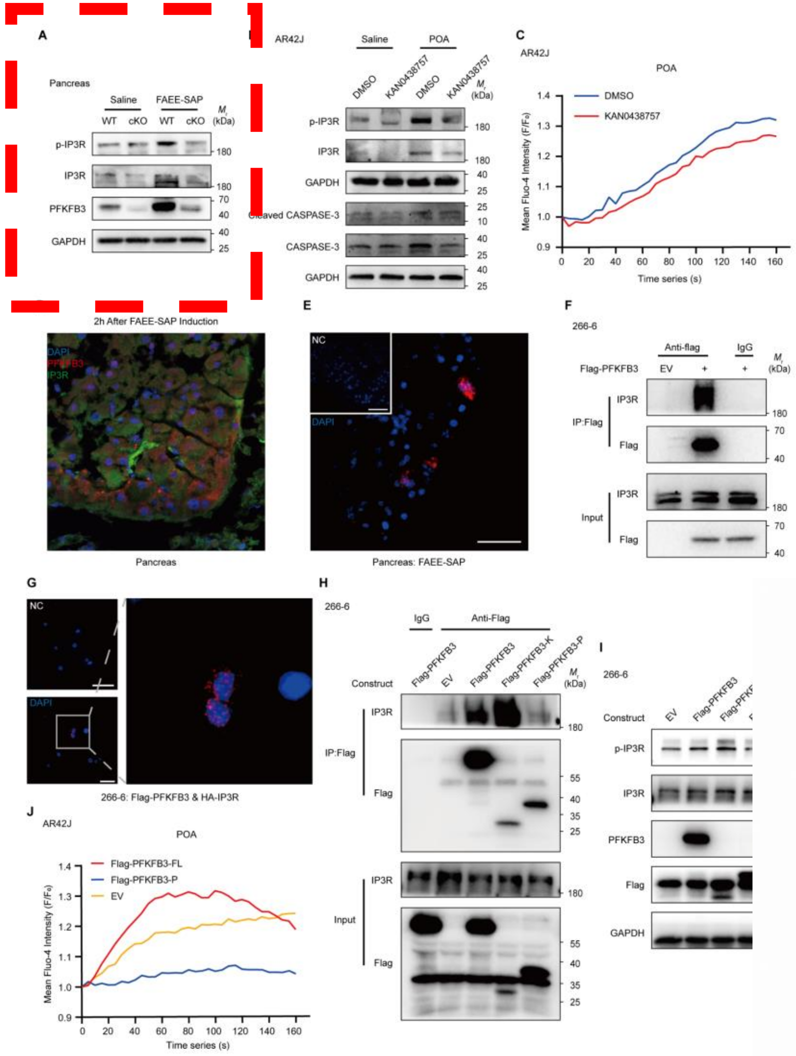

p-IP3R

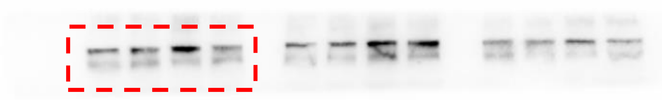

IP3R

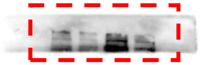

PFKFB3

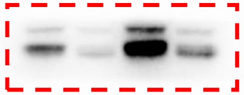

GAPDH

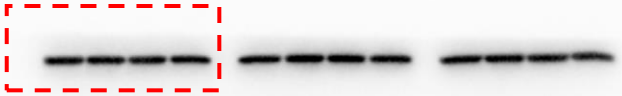

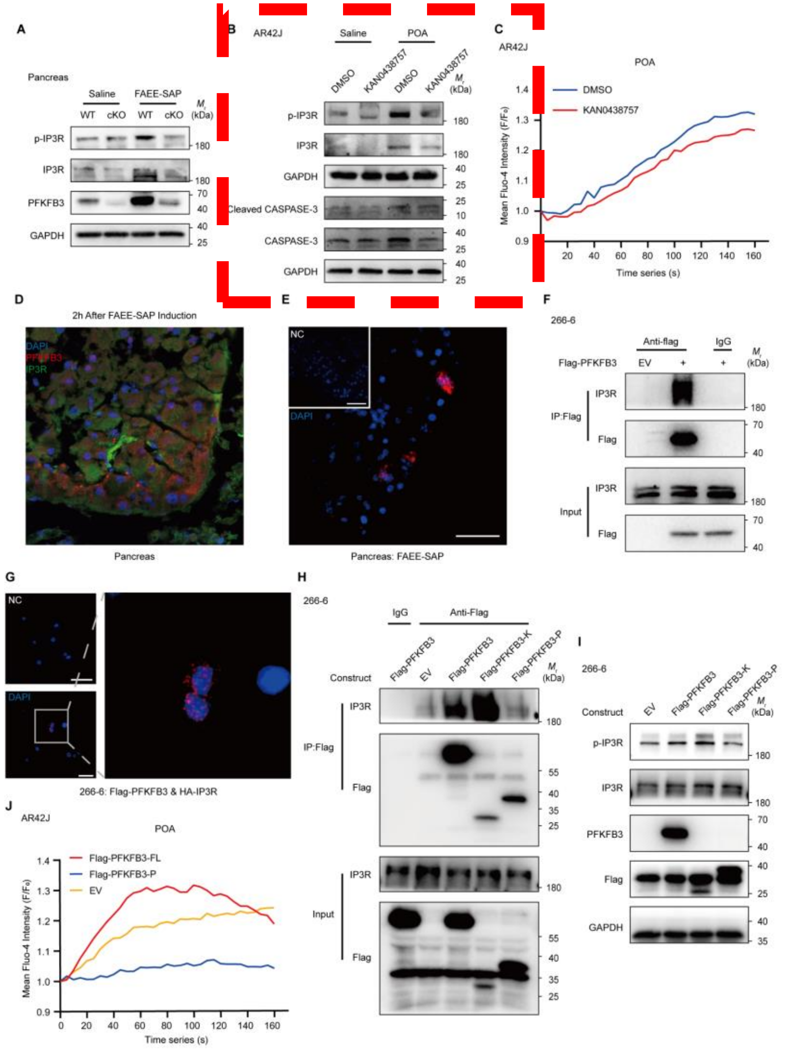

Full unedited gel for Figure.6B

Samples run at different times.
Phosphorylated protein antibody was incubated on the same membrane after elution of total protein antibody.

IP3R

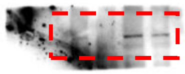

p-IP3R

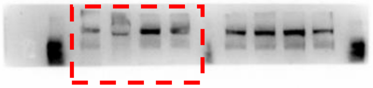

GAPDH

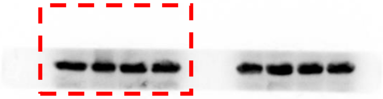

Cleaved CASPASE-3

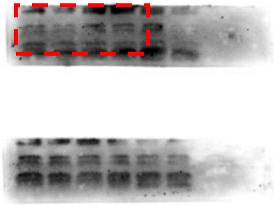

GAPDH

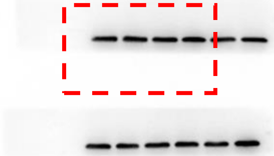

CASPASE-3

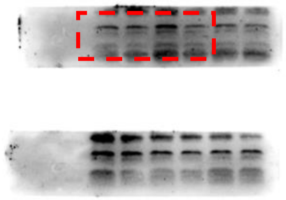

Full unedited gel for Figure.6F

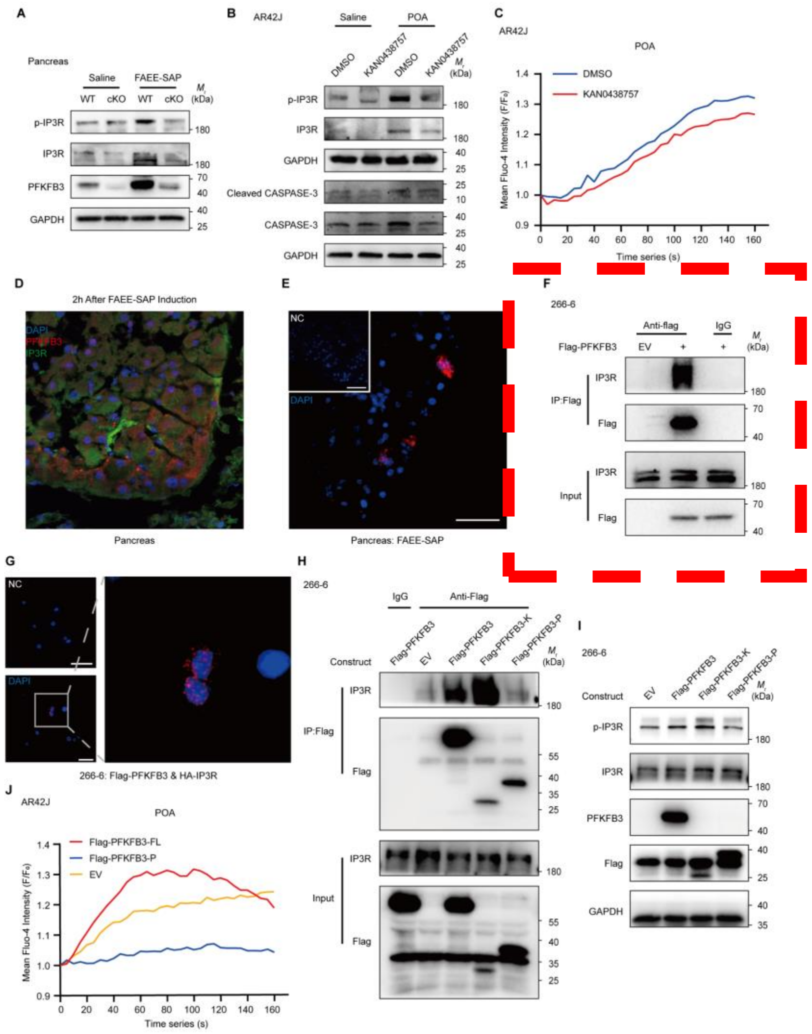

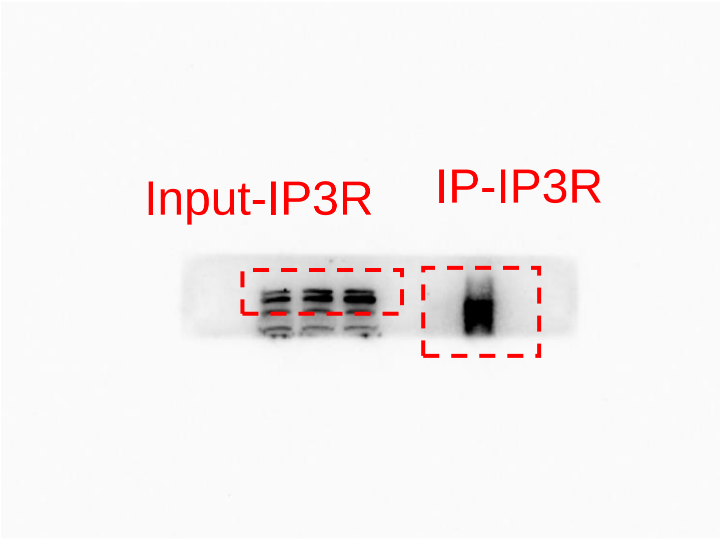

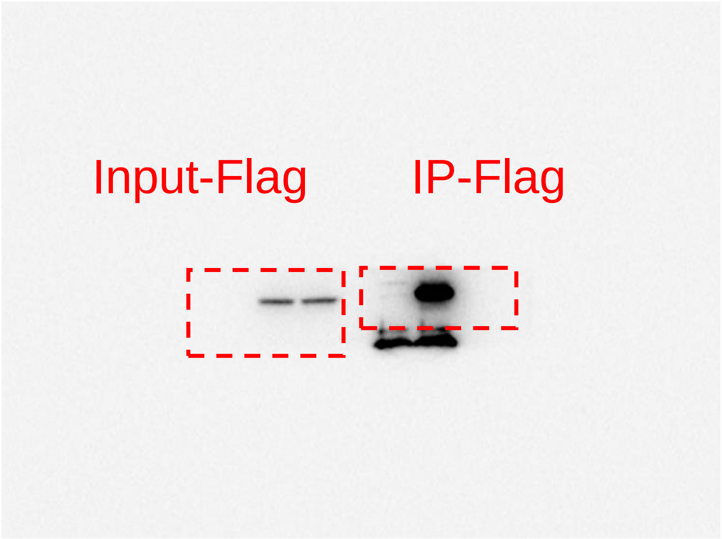

Full unedited gel for Figure.6H

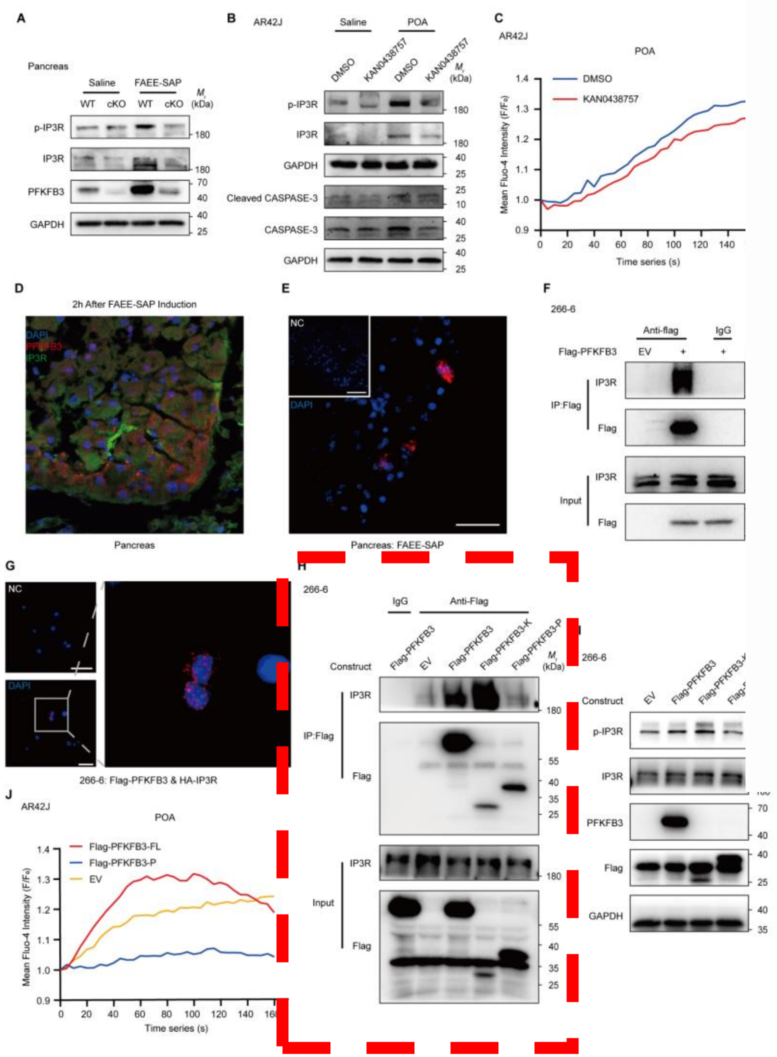

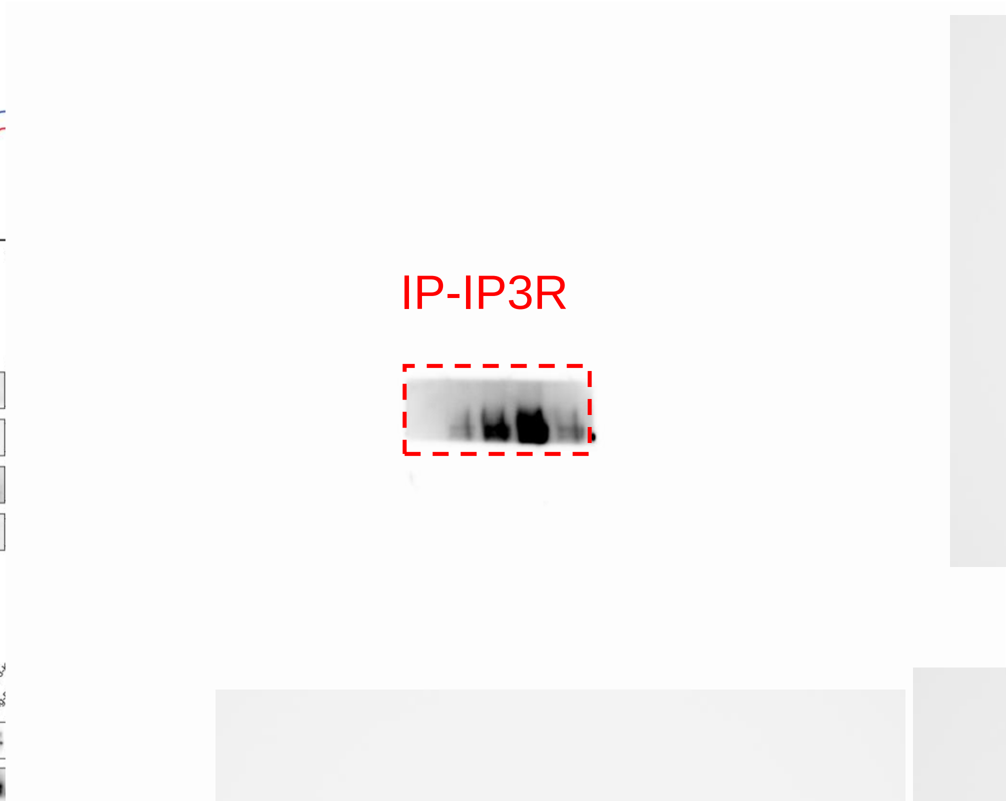

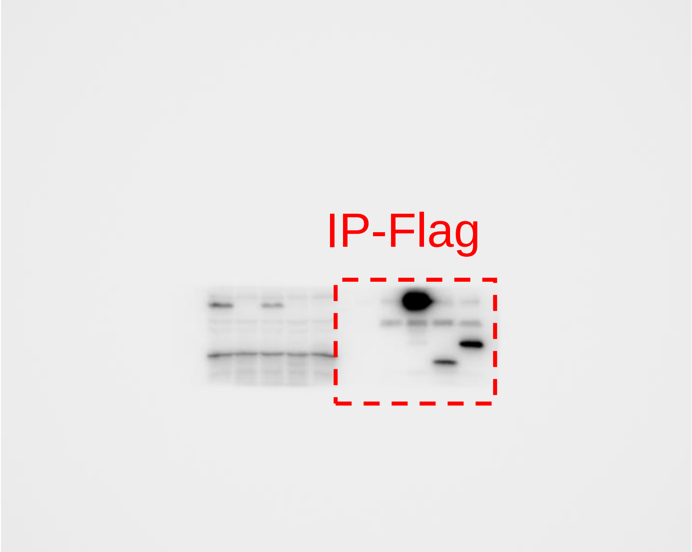

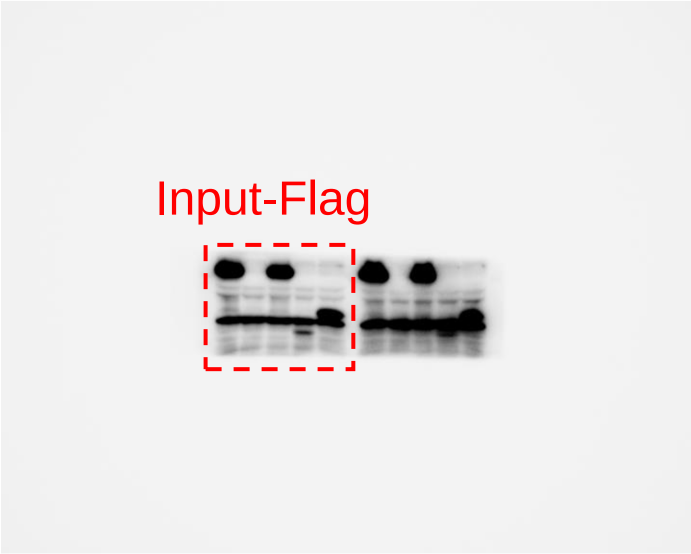

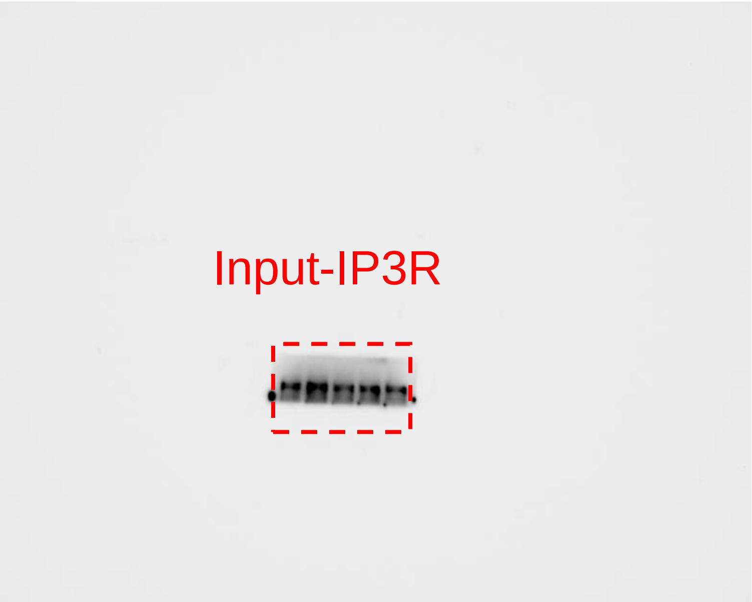

Full unedited gel for Figure.6I

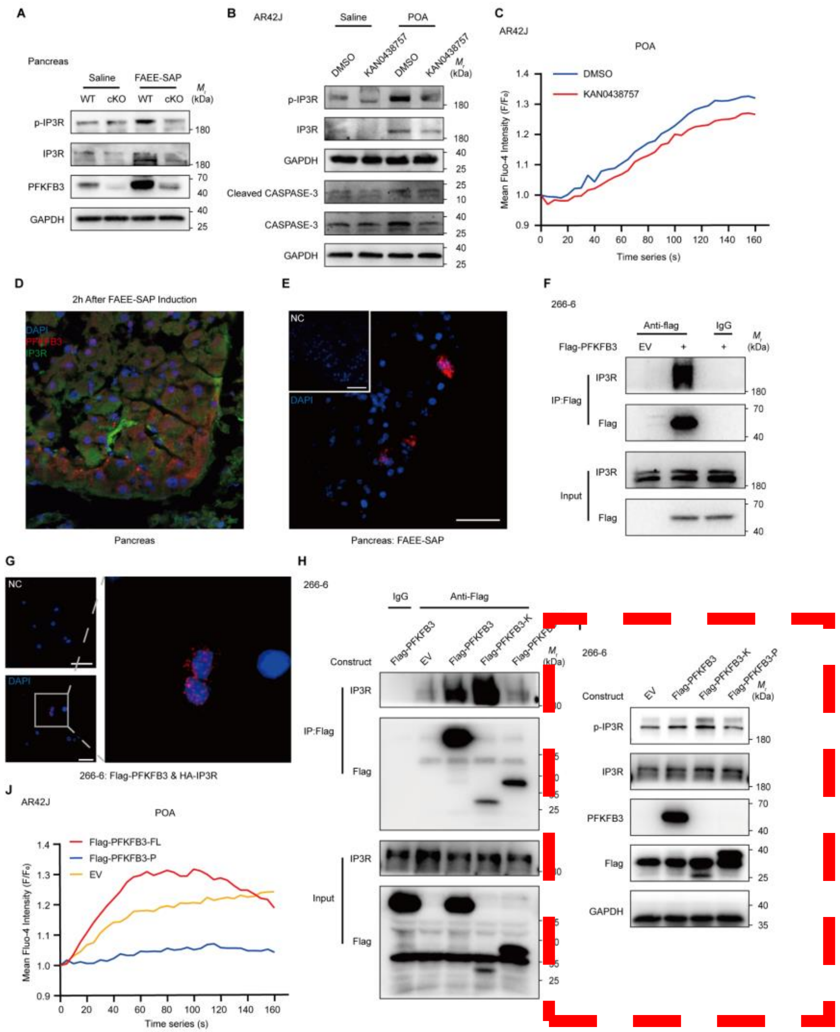

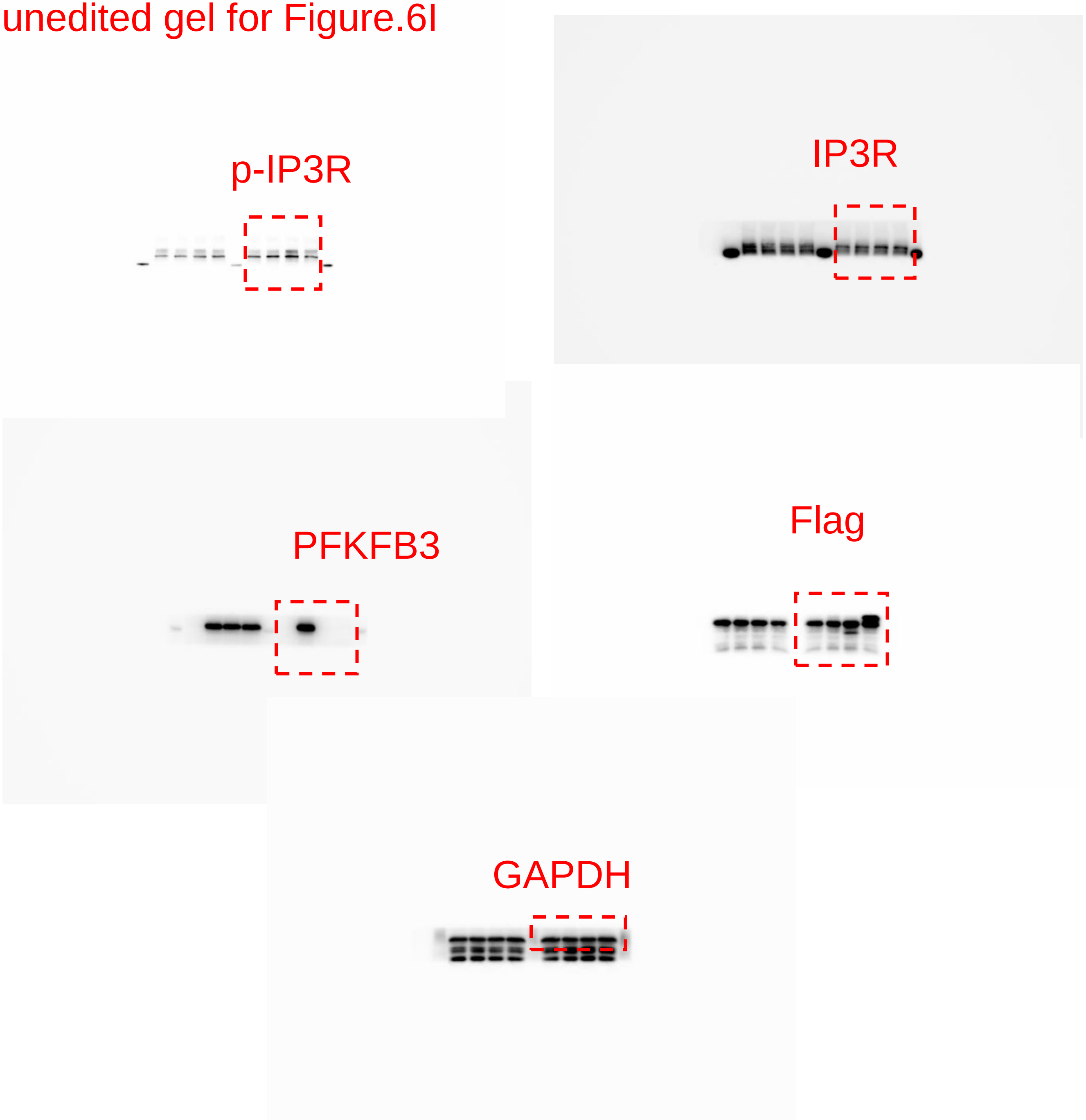

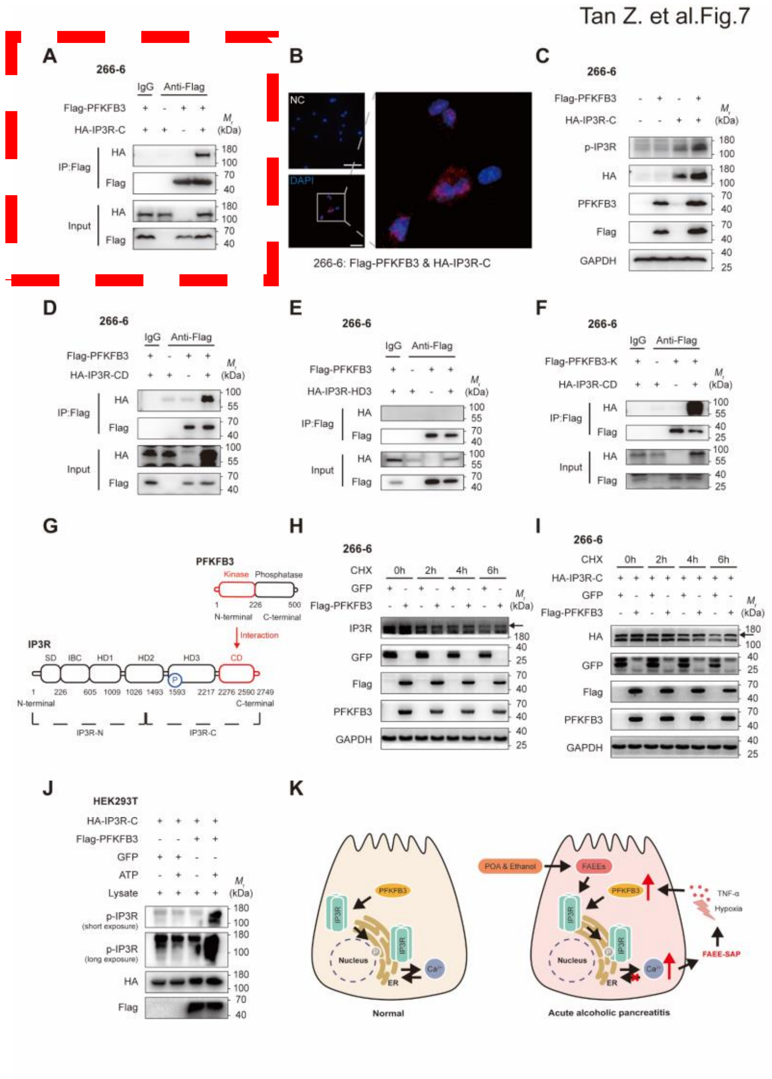

Full unedited gel for Figure.7A

IP-HA

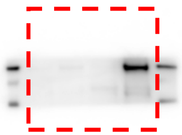

IP-Flag

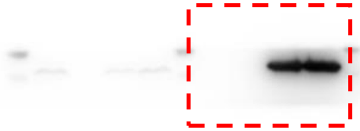

Input-HA

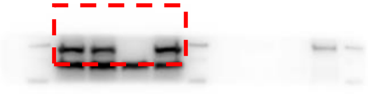

Input-Flag

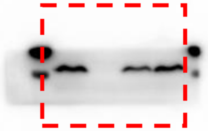

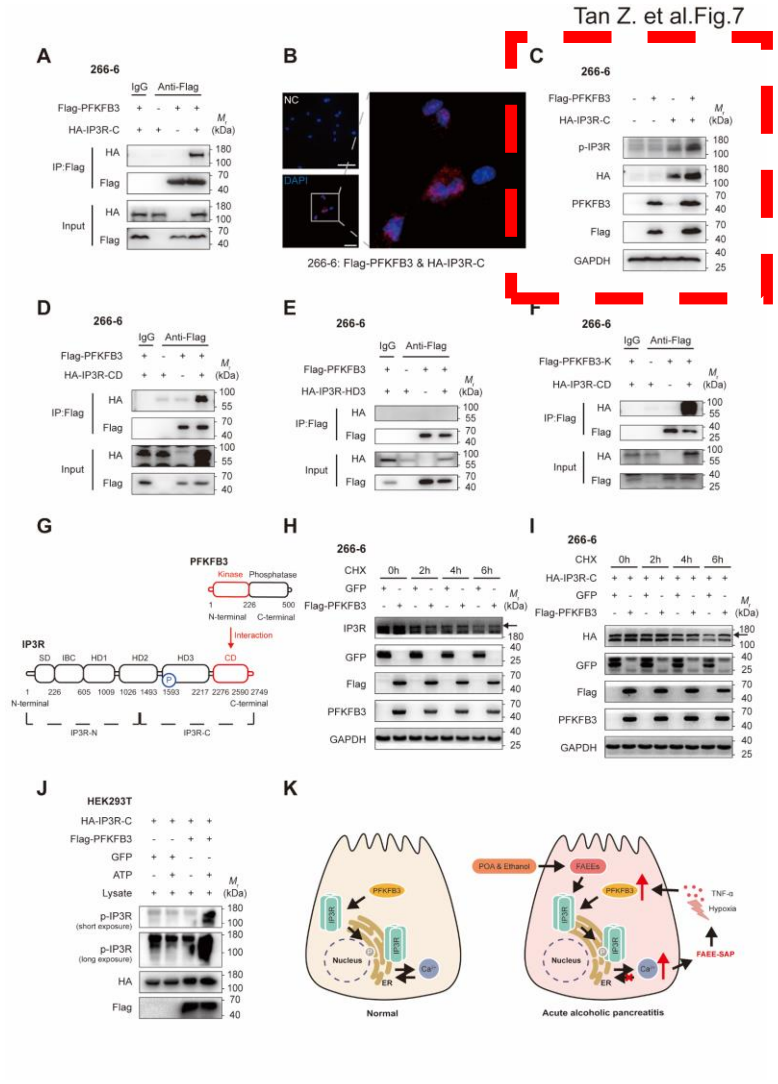

Full unedited gel for Figure.7C

Western Blots set up in parallel and run contemporaneously.

p-IP3R

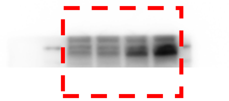

HA

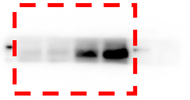

PFKFB3

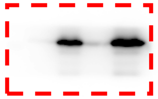

Flag

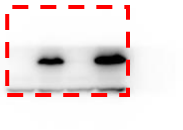

GAPDH

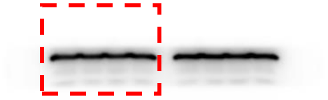

Full unedited gel for Figure.7D

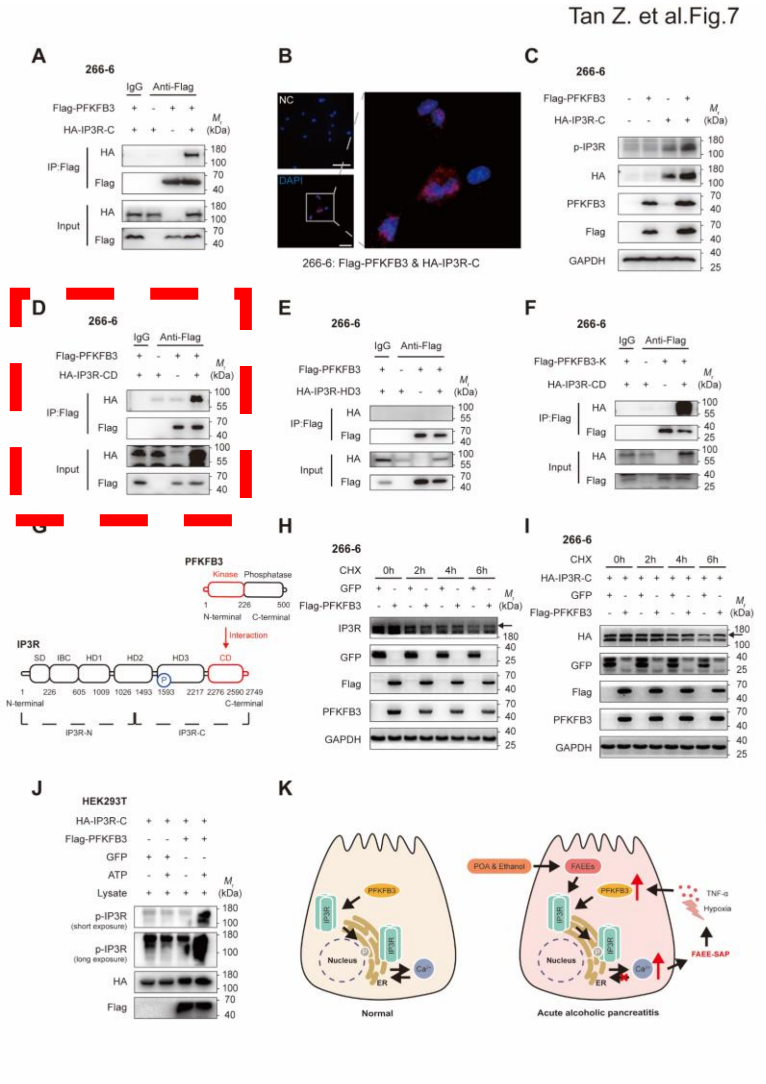

Input-Flag

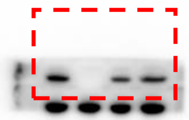

Input-HA

IP-HA

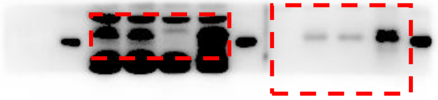

IP-Flag

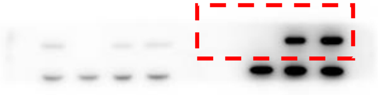

Full unedited gel for Figure.7E

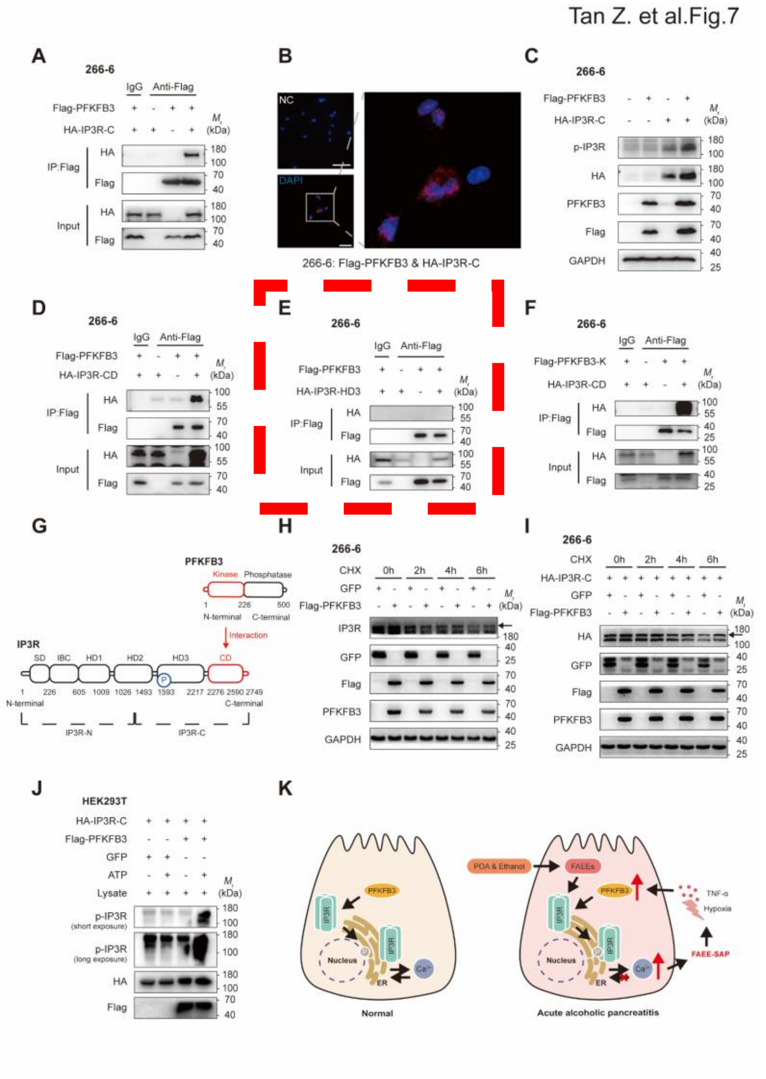

IP-HA

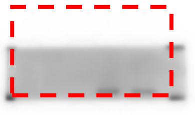

Input-HA

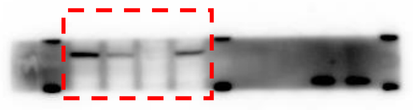

IP-Flag

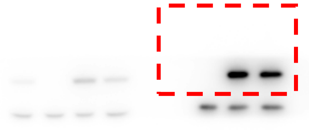

Input-Flag

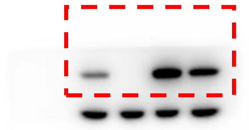

Full unedited gel for Figure.7F

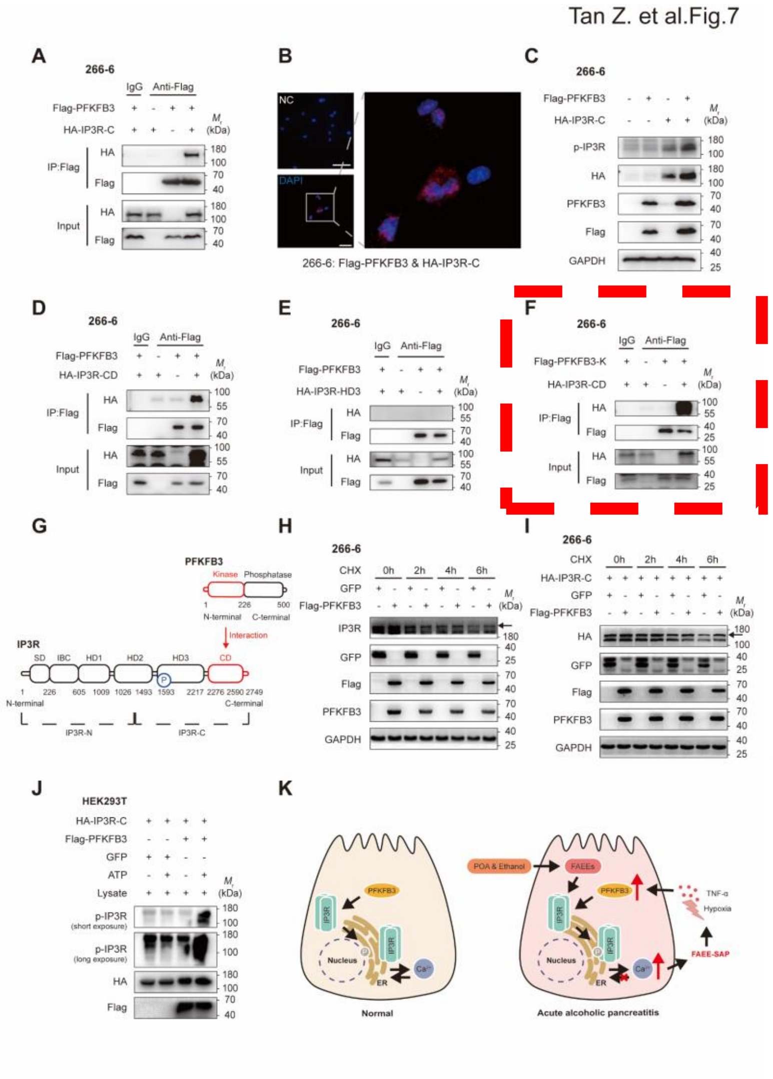

IP-HA

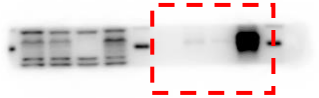

Input-HA

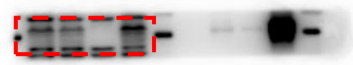

IP-Flag

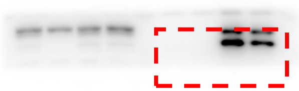

Input-Flag

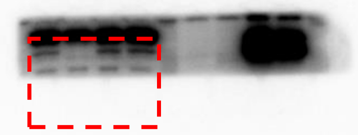

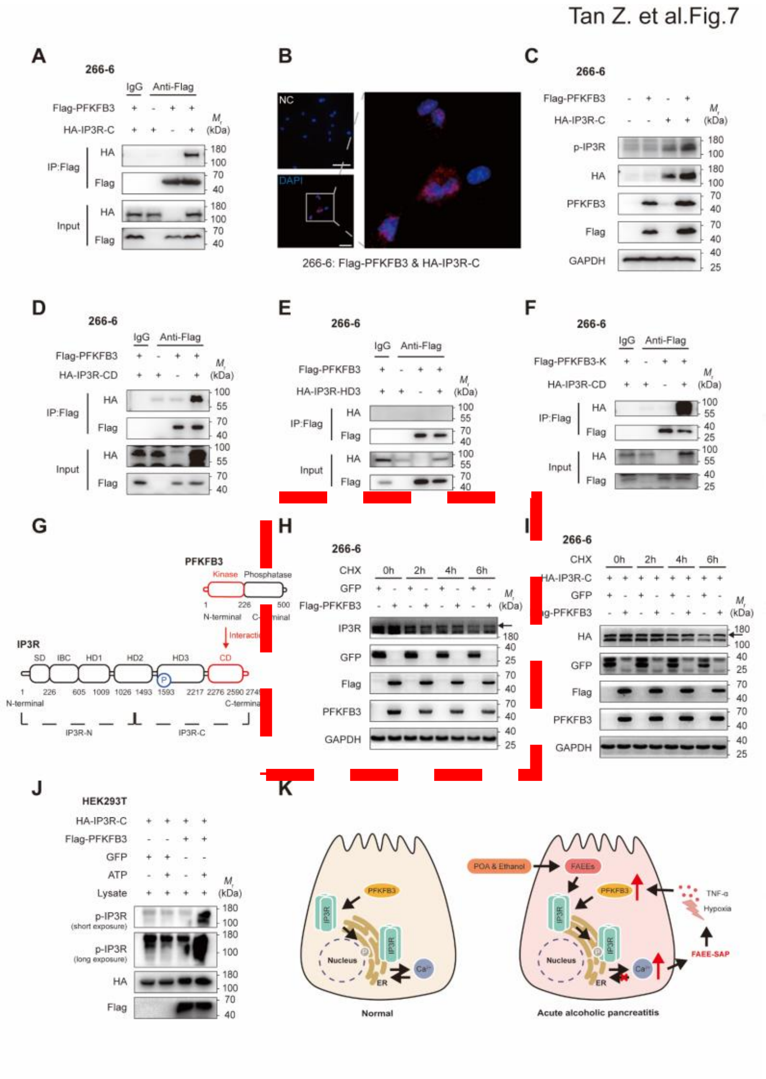

Full unedited gel for Figure.7H

IP3R

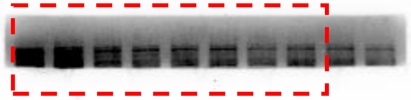

GFP

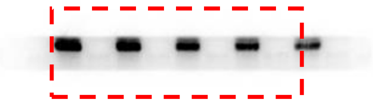

Flag

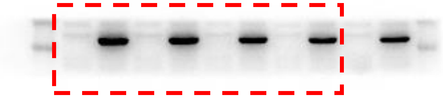

PFKFB3

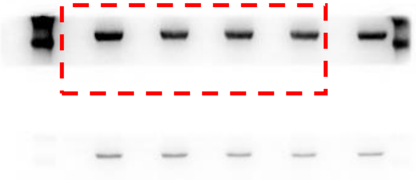

GAPDH

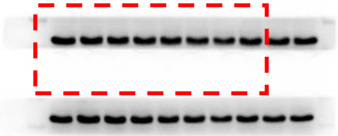

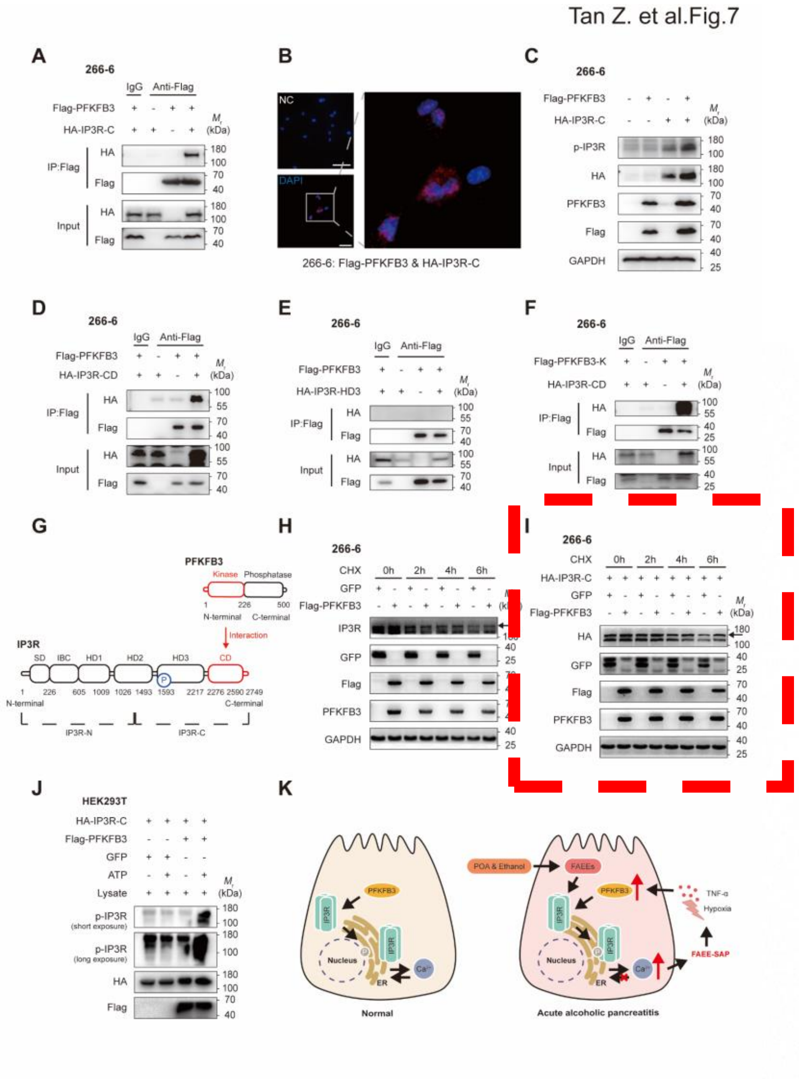

Full unedited gel for Figure.7I

HA

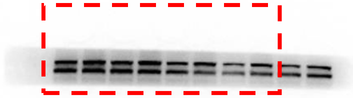

GFP

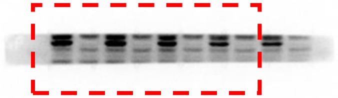

PFKFB3

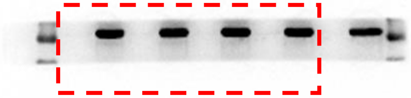

Flag

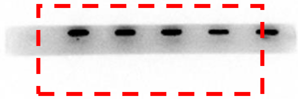

GAPDH

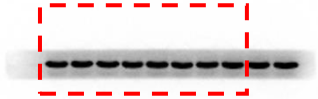

Full unedited gel for Figure.7J

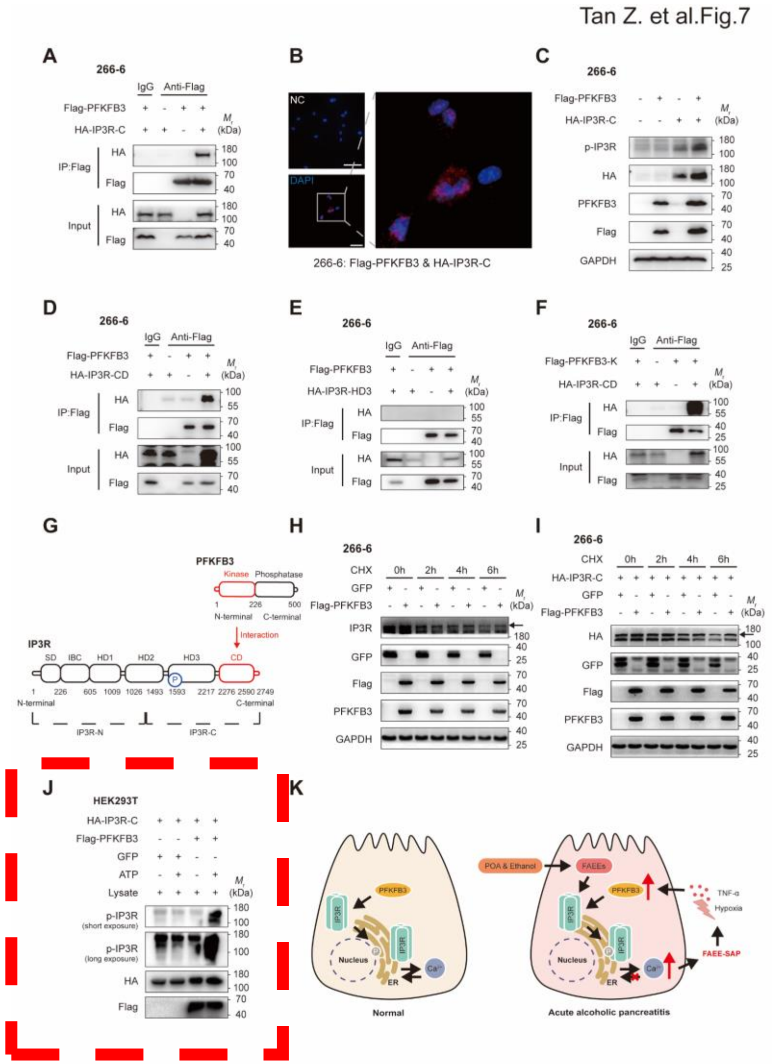

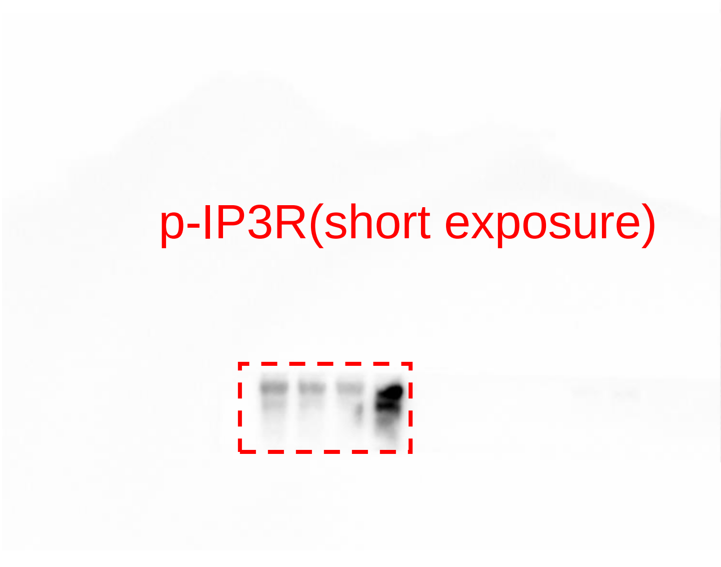

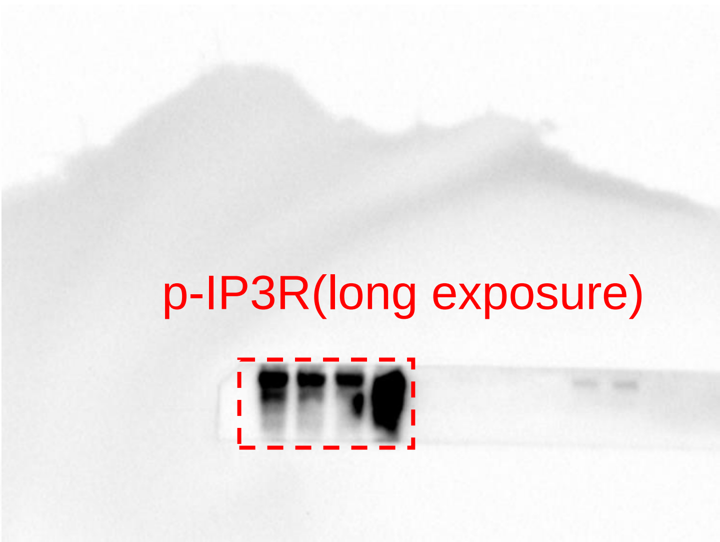

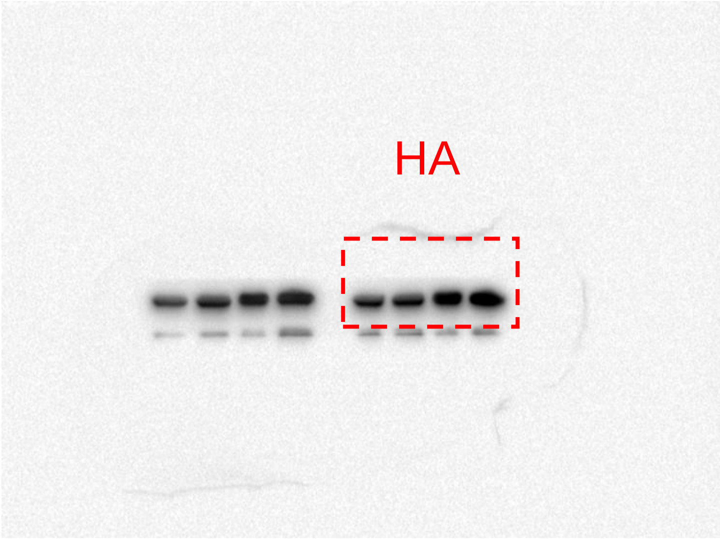

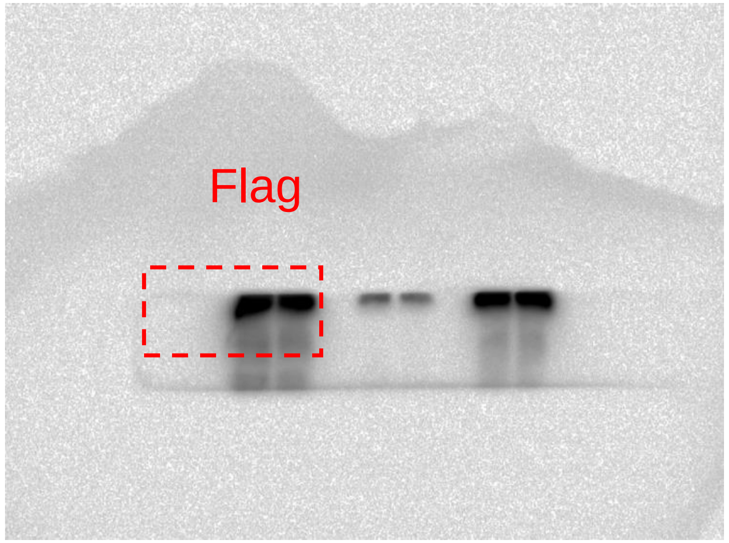

Full unedited gel for Figure.S1C

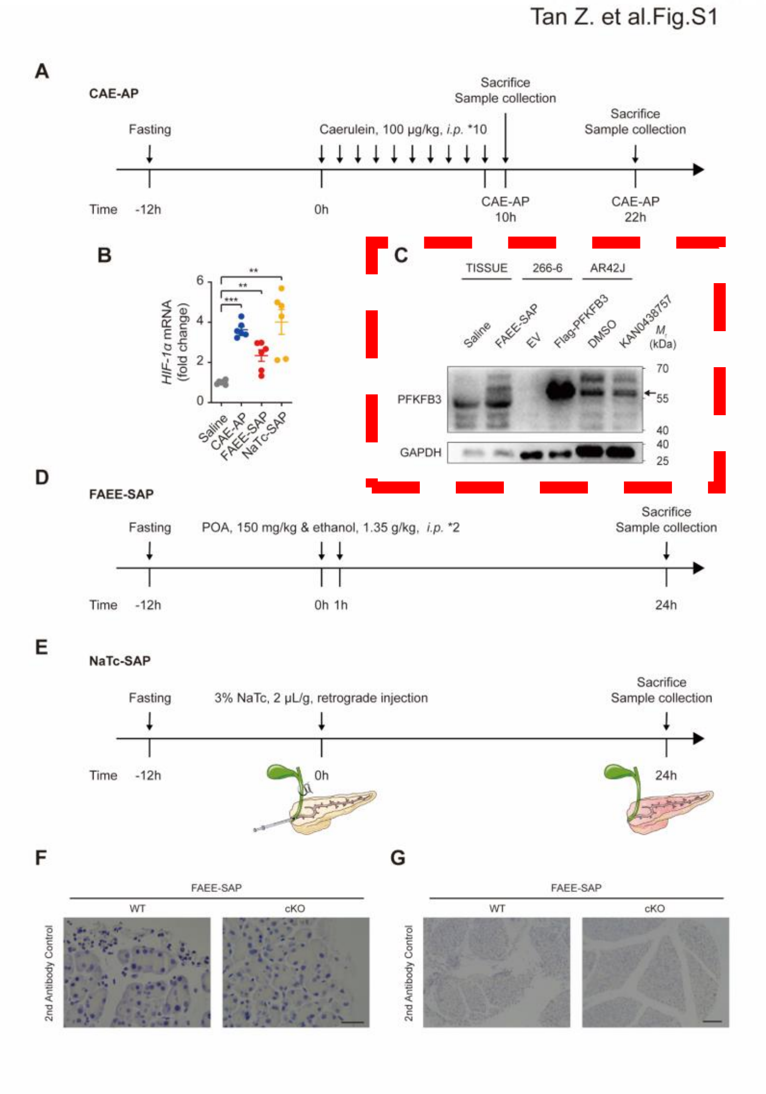

PFKFB3

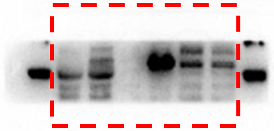

GAPDH

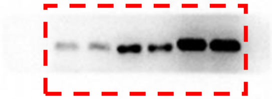

Full unedited gel for Figure.S2E

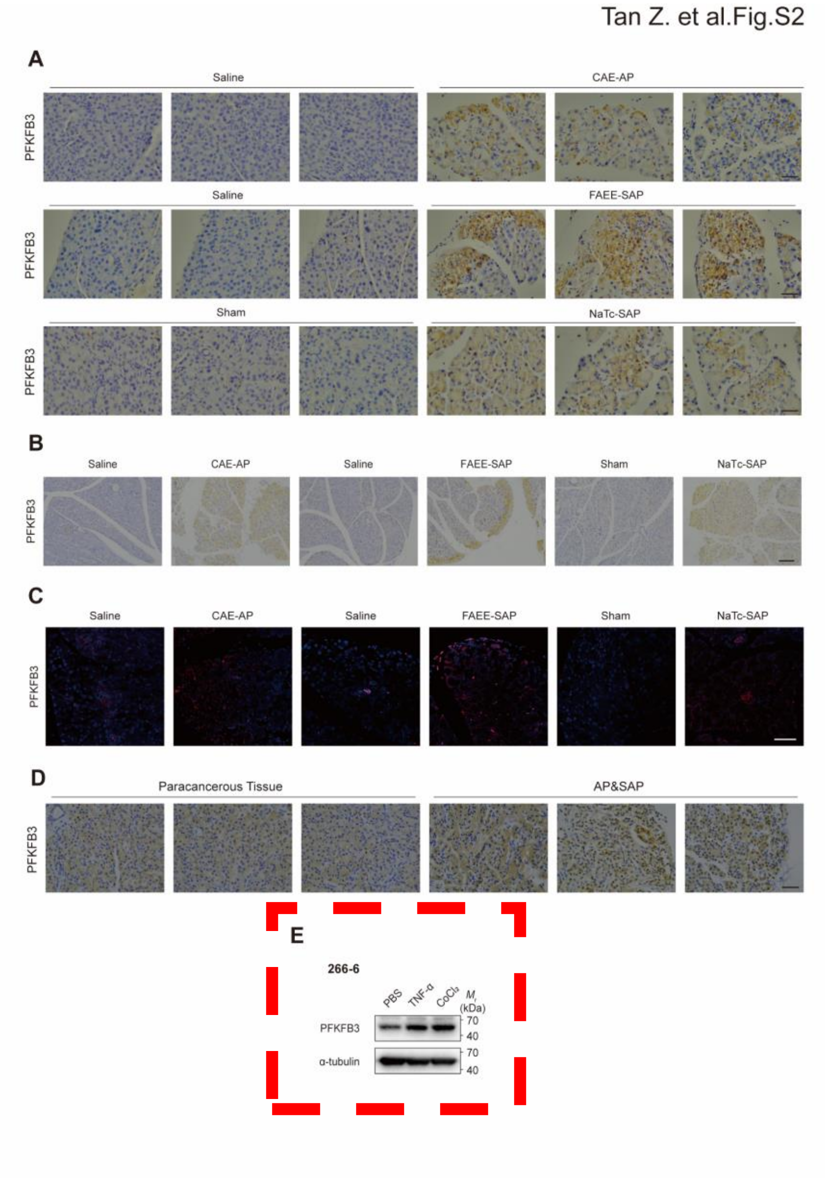

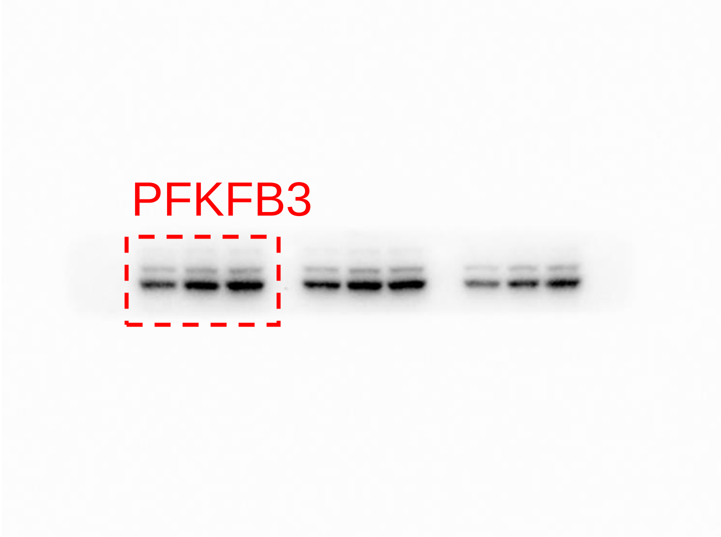

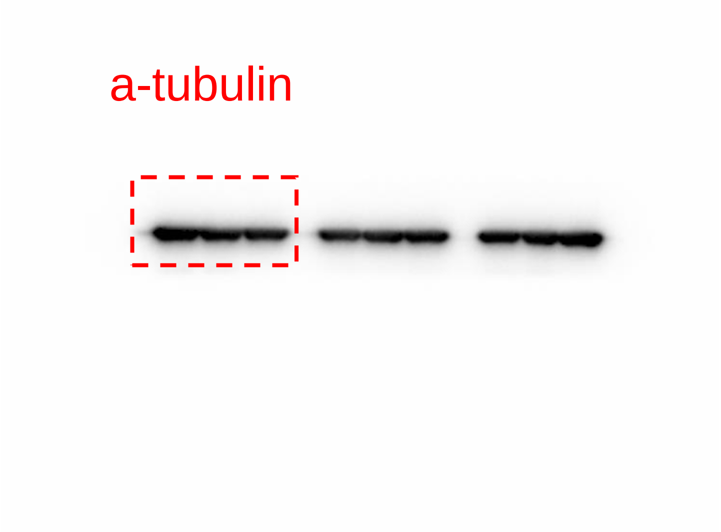

Full unedited gel for Figure.S3D

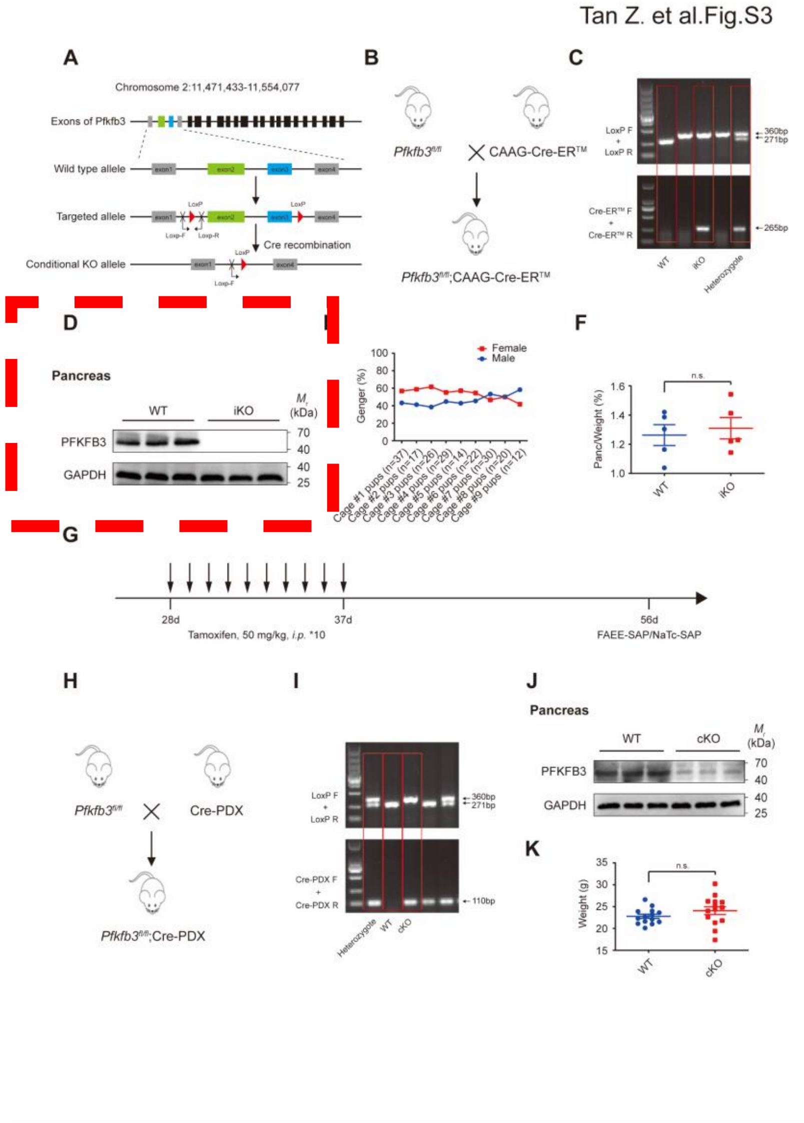

PFKFB3

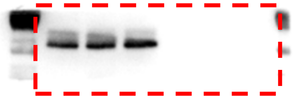

GAPDH

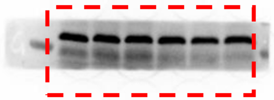

Full unedited gel for Figure.S3J

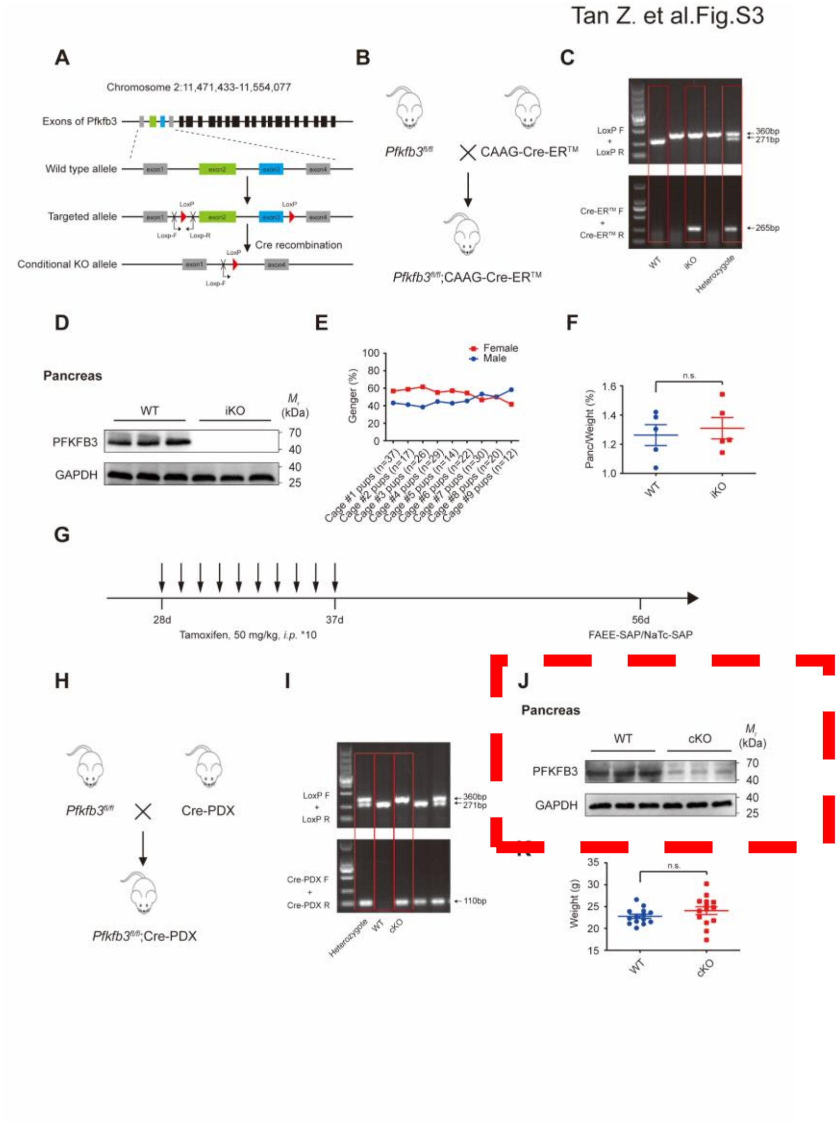

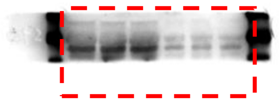

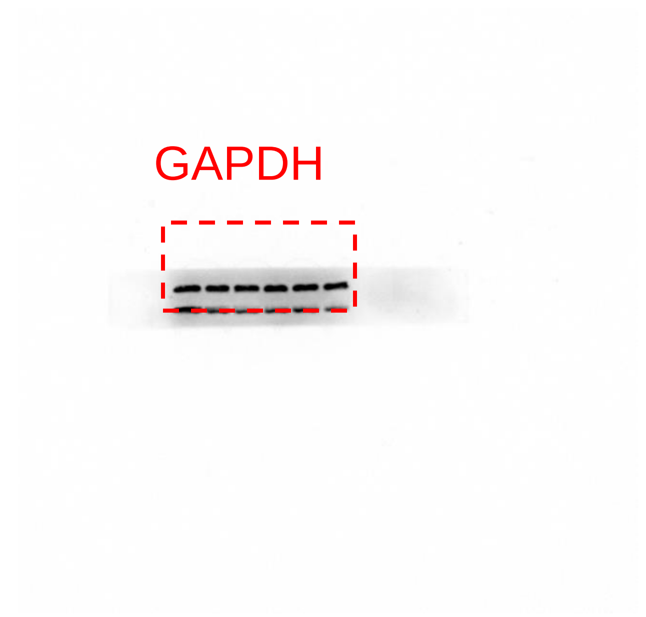

Full unedited gel for Figure.S11C

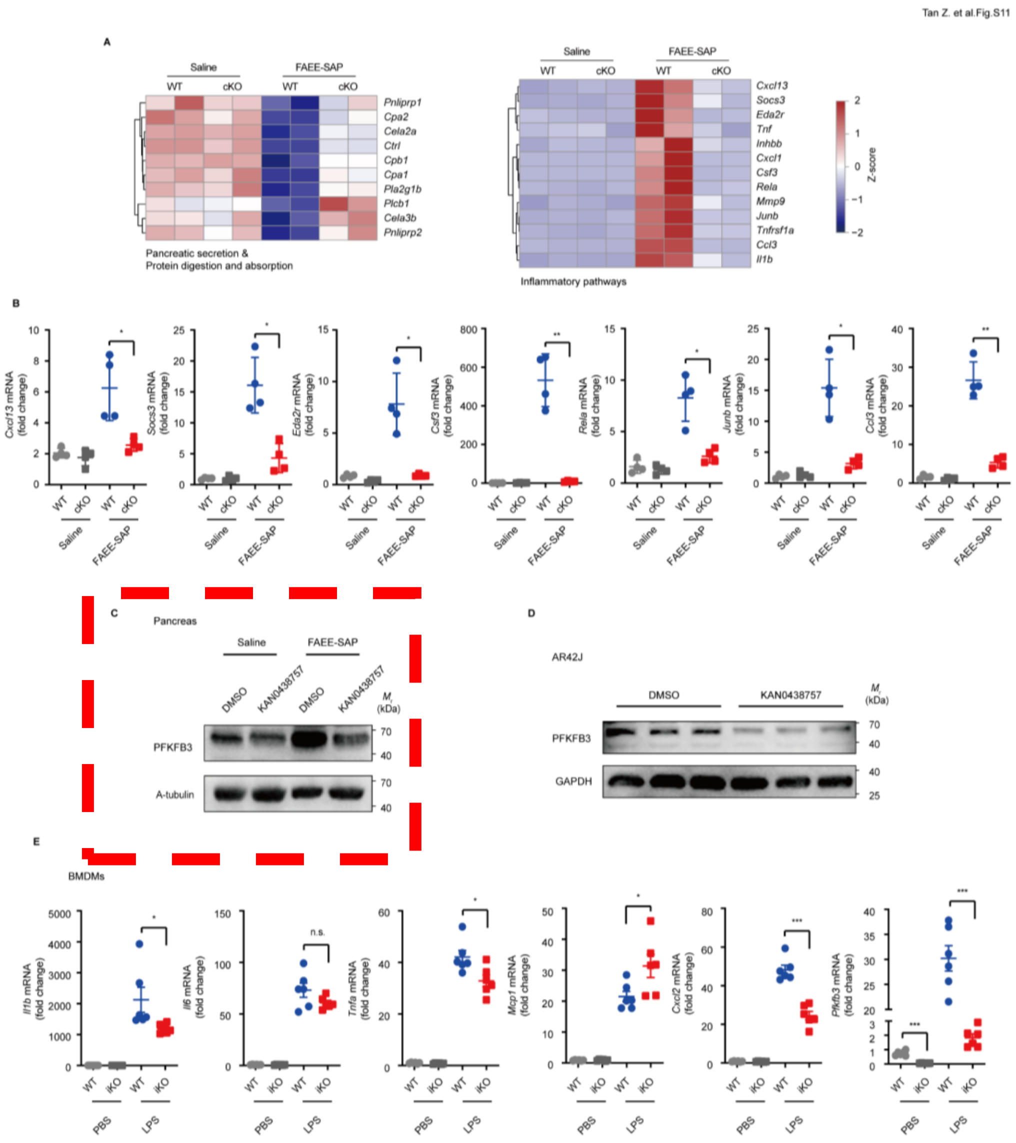

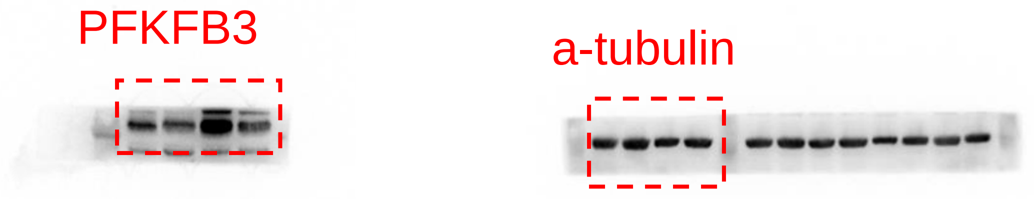

Full unedited gel for Figure.S11D

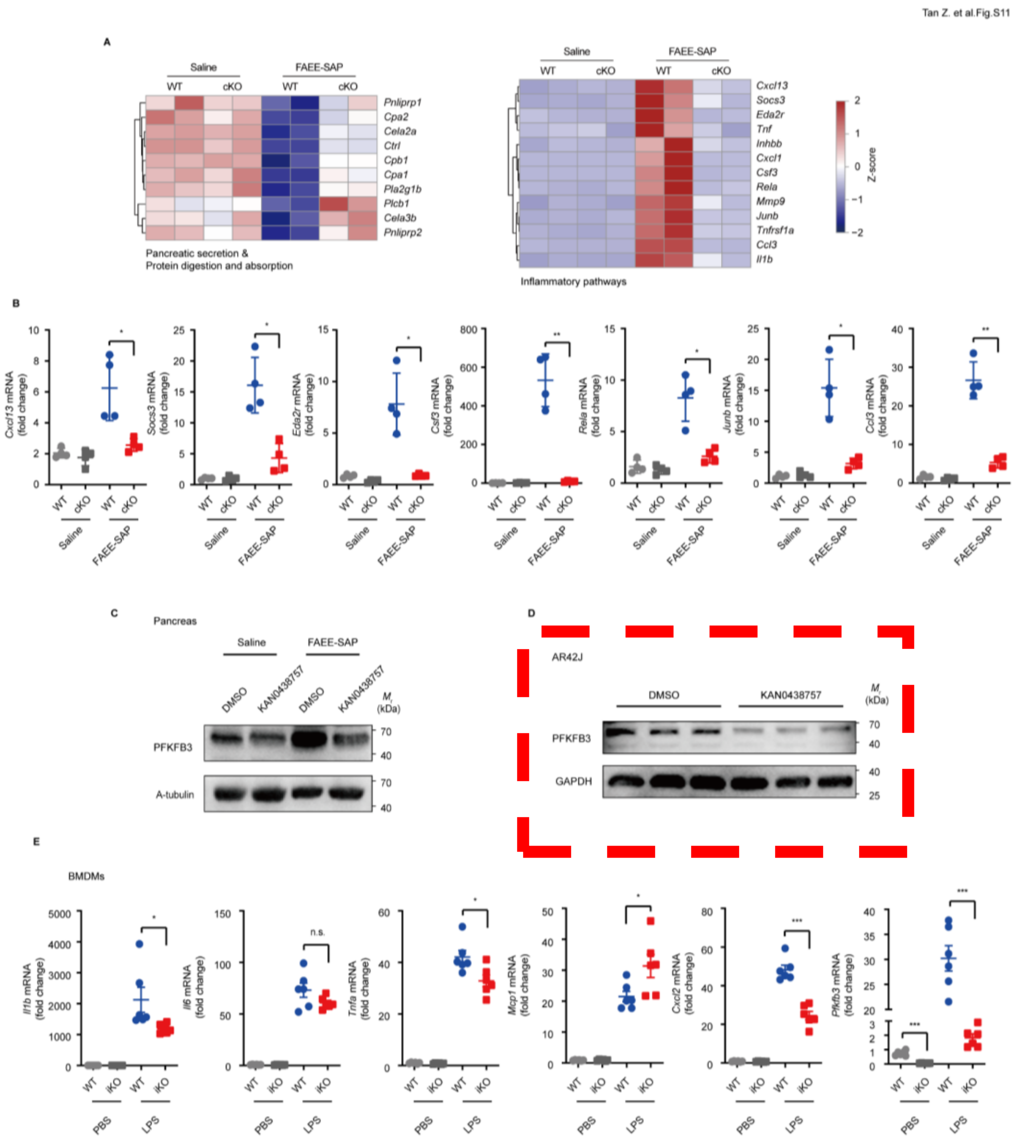

PFKFB3

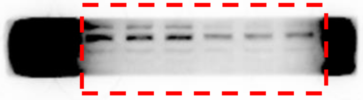

GAPDH

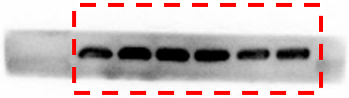

Full unedited gel for Figure.S12F

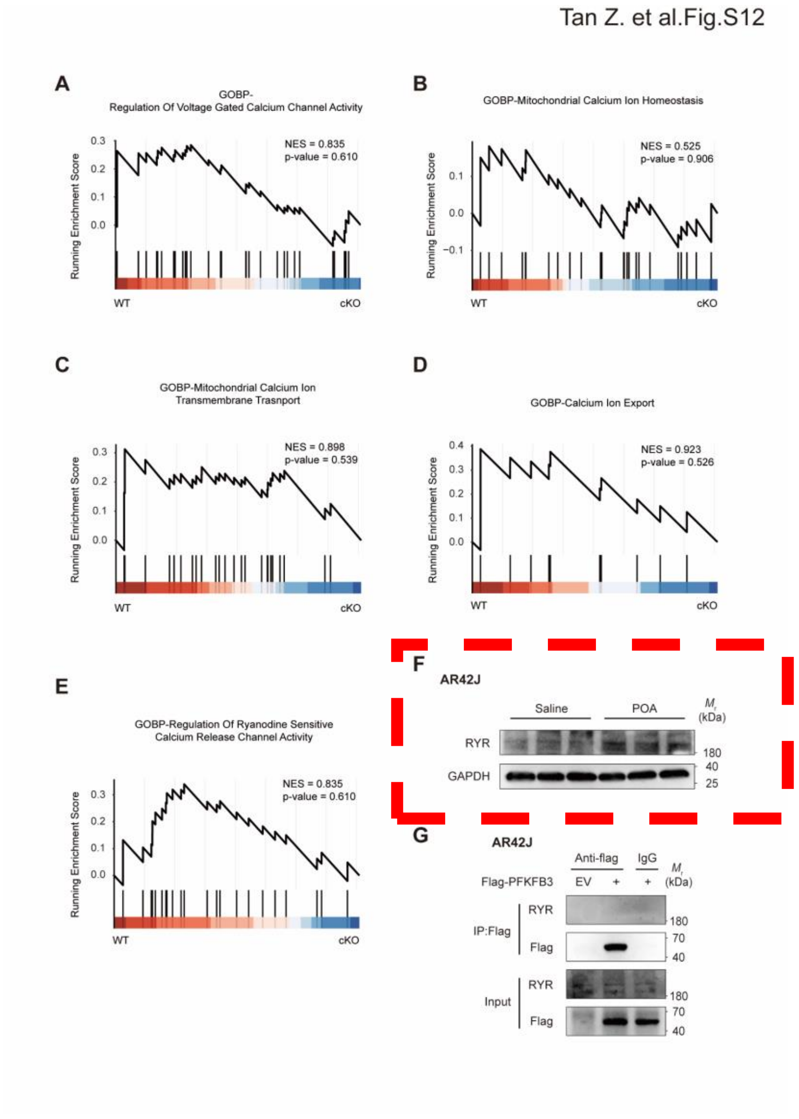

RYR

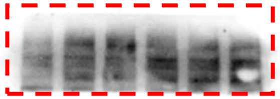

GAPDH

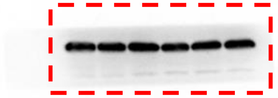

Full unedited gel for Figure.S12G

Tan Z. et al.Fig.S12

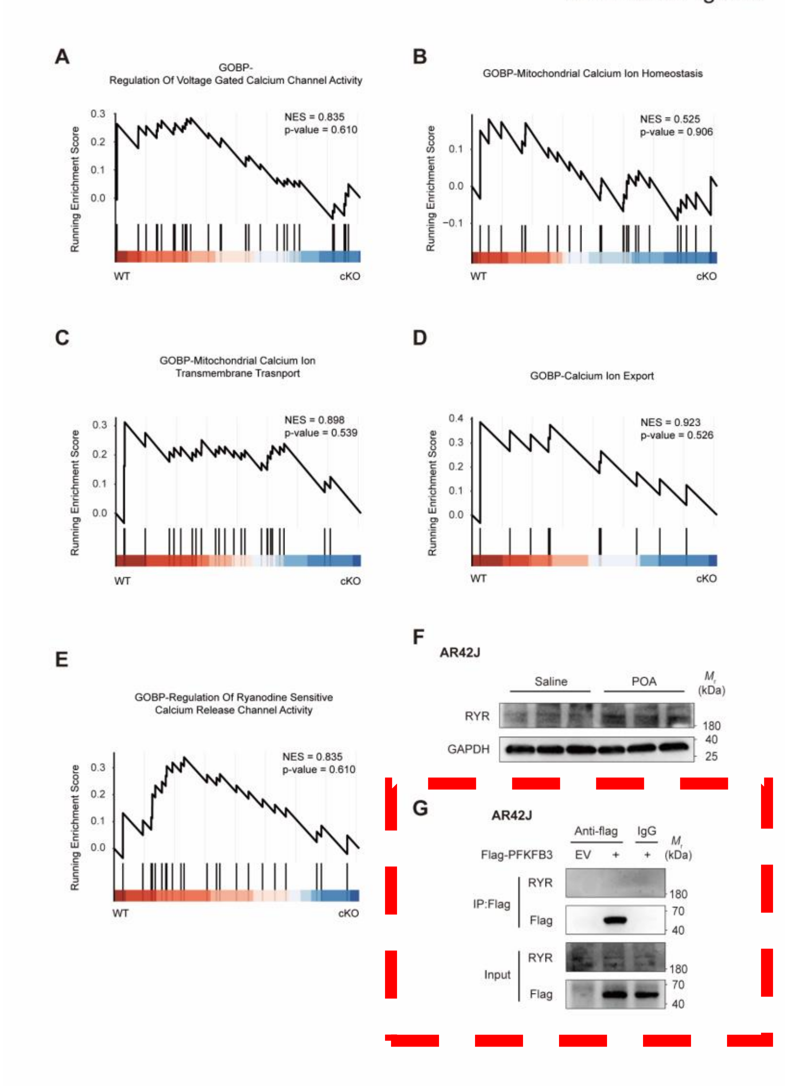

IP-RYR

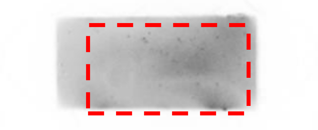

IP-Flag

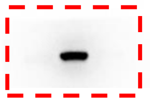

Input-RYR

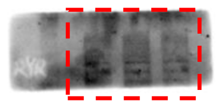

Input-Flag

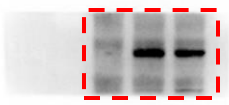

Full unedited gel for Figure.S14B

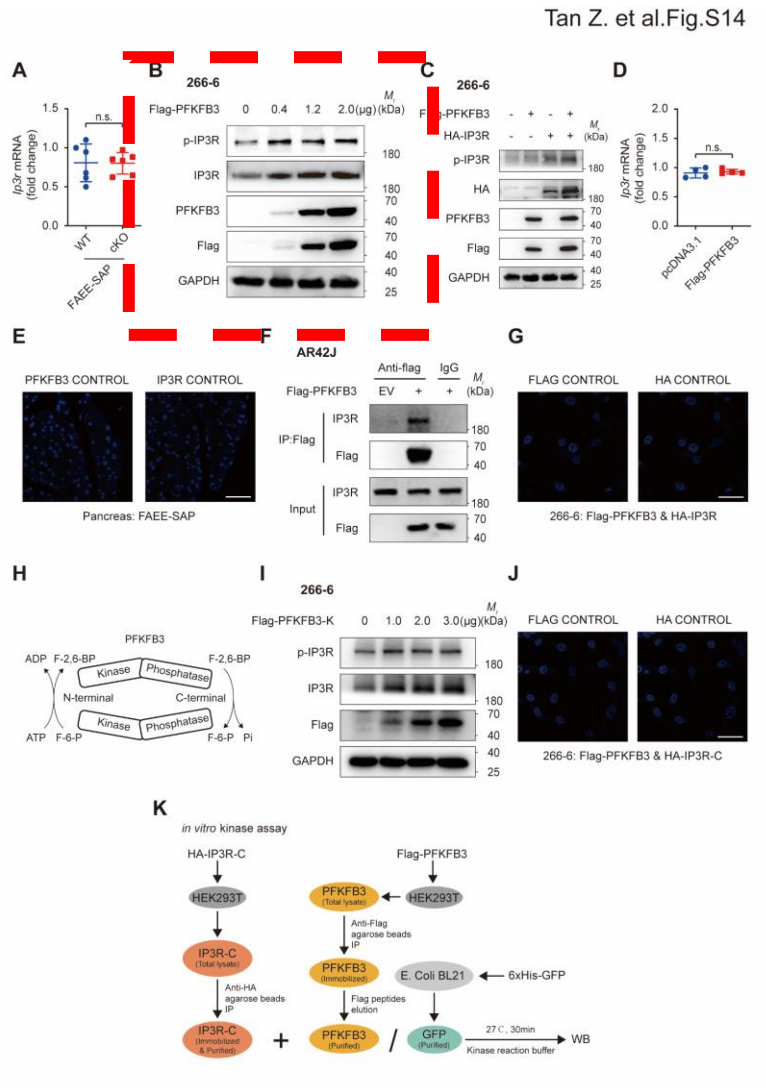

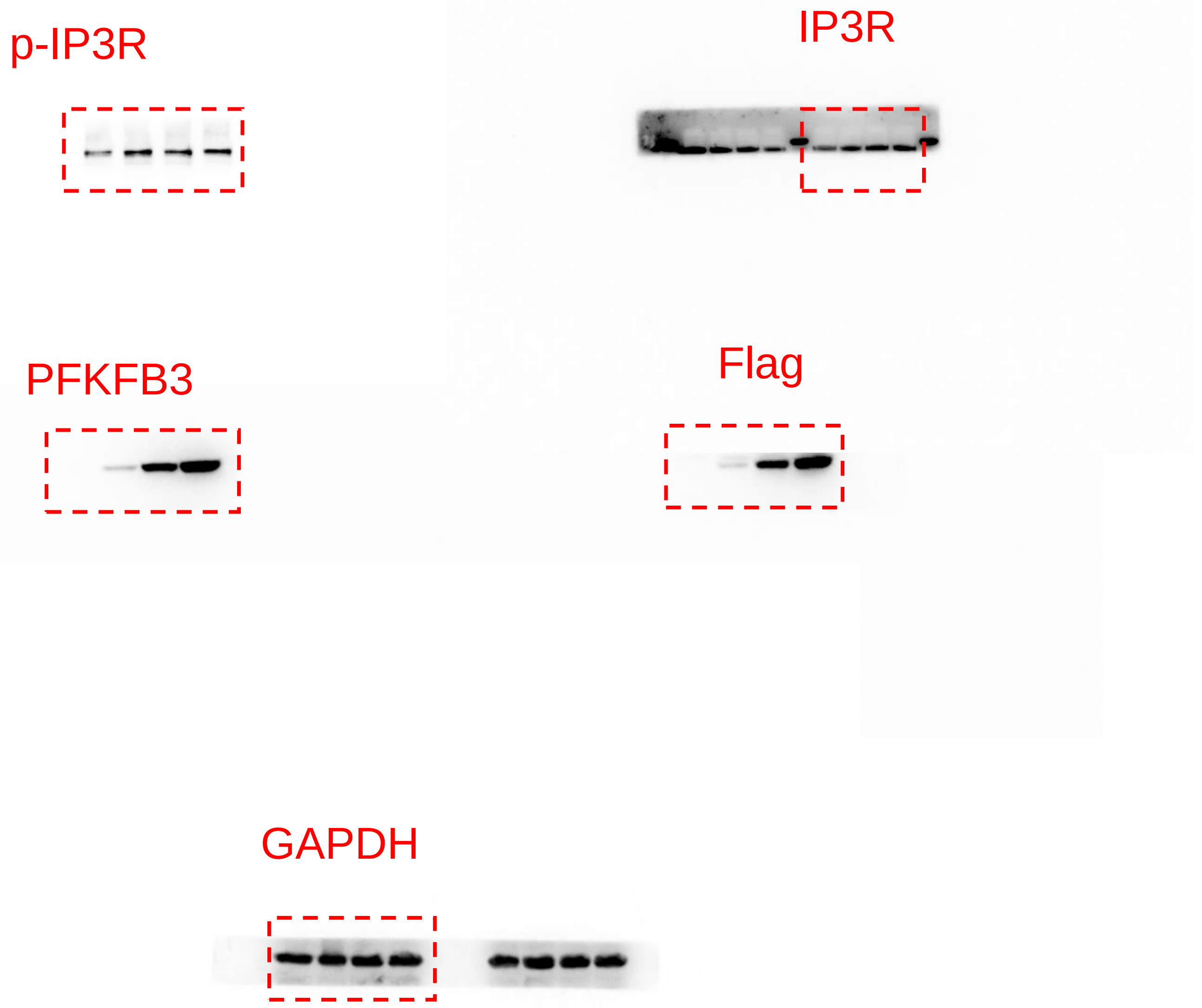

Full unedited gel for Figure.S14C

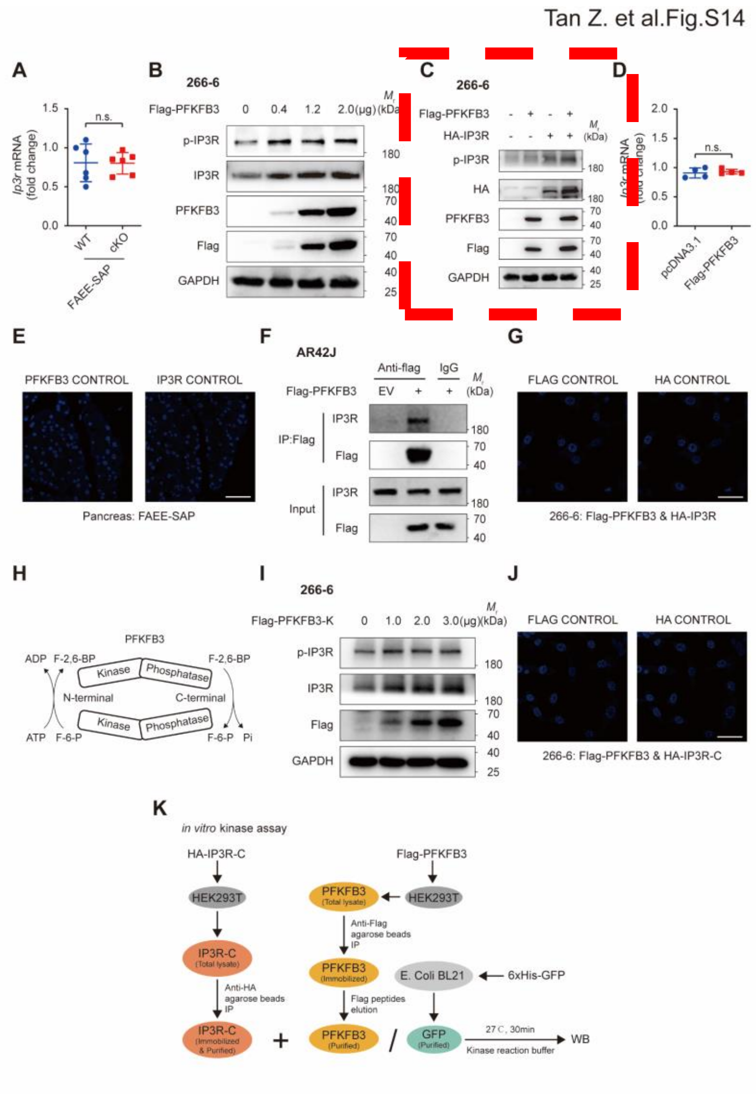

p-IP3R

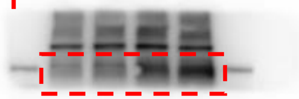

HA

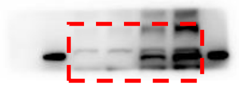

PFKFB3

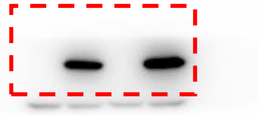

Flag

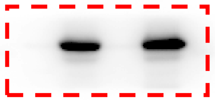

GAPDH

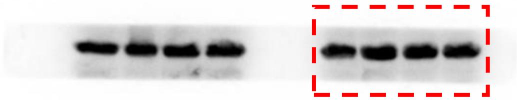

Full unedited gel for Figure.S14F

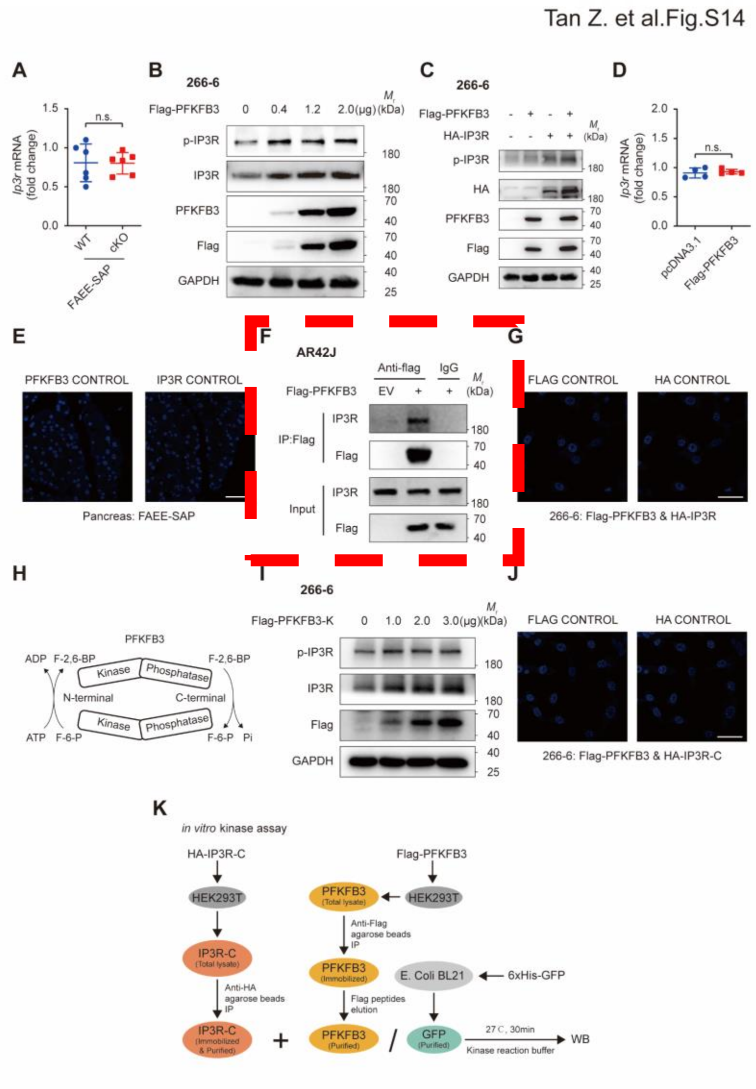

IP-IP3R

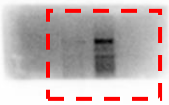

IP-Flag

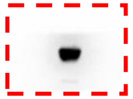

Input-IP3R

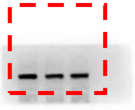

Input-Flag

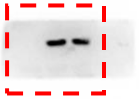

Full unedited gel for Figure.S14I

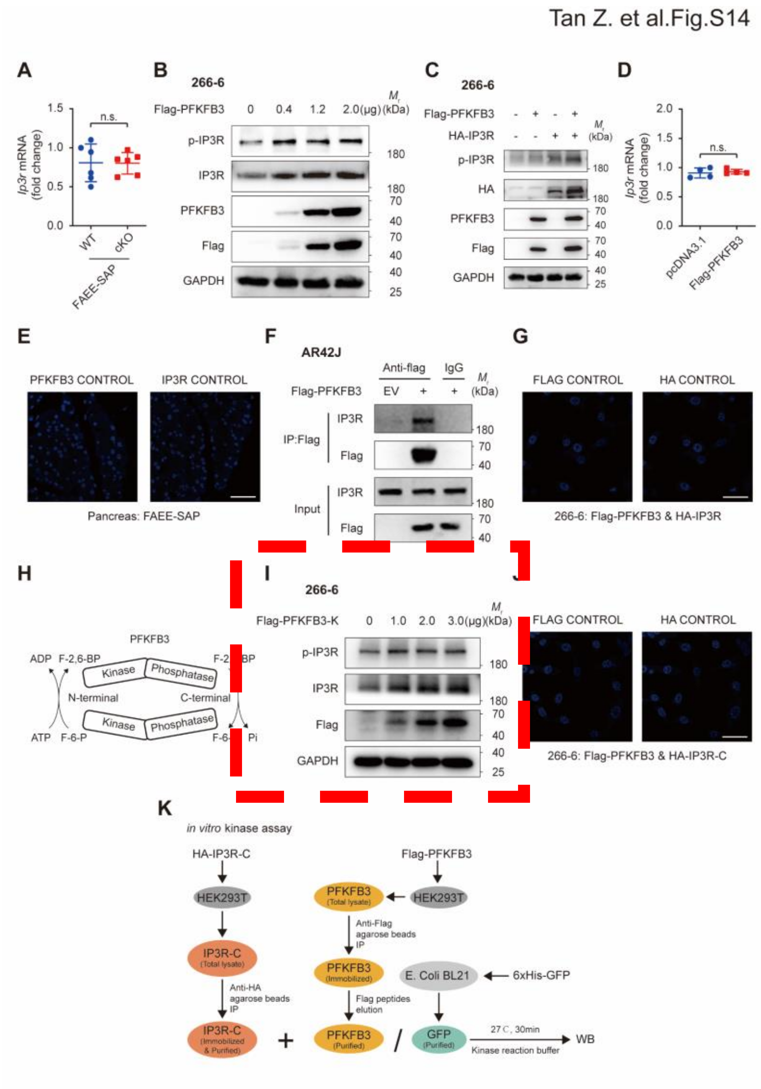

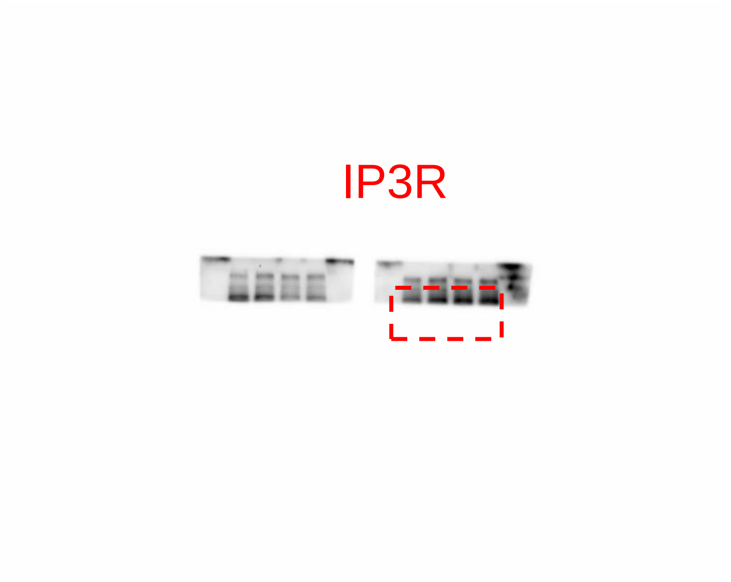

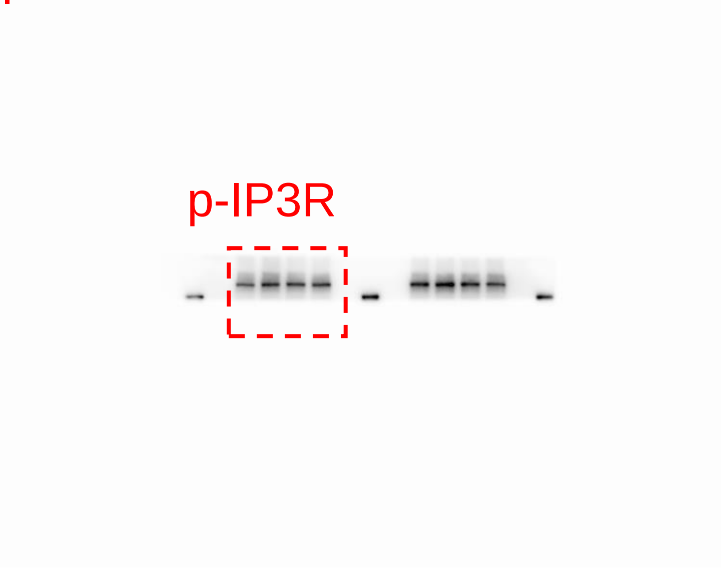

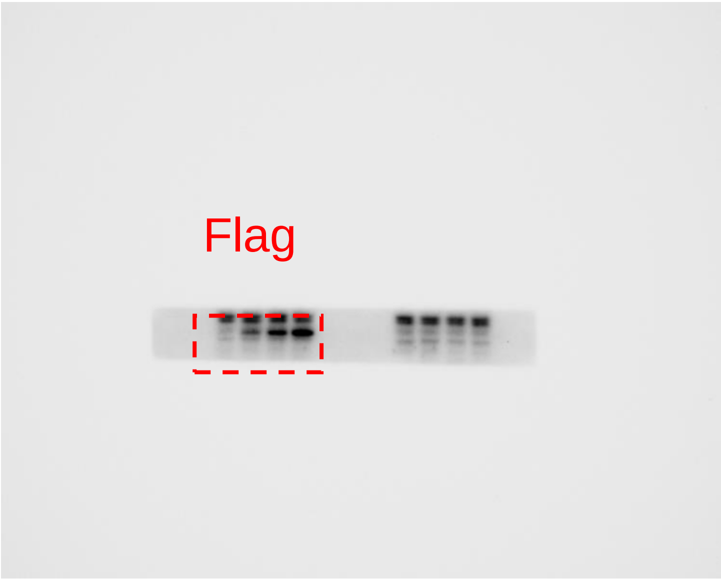

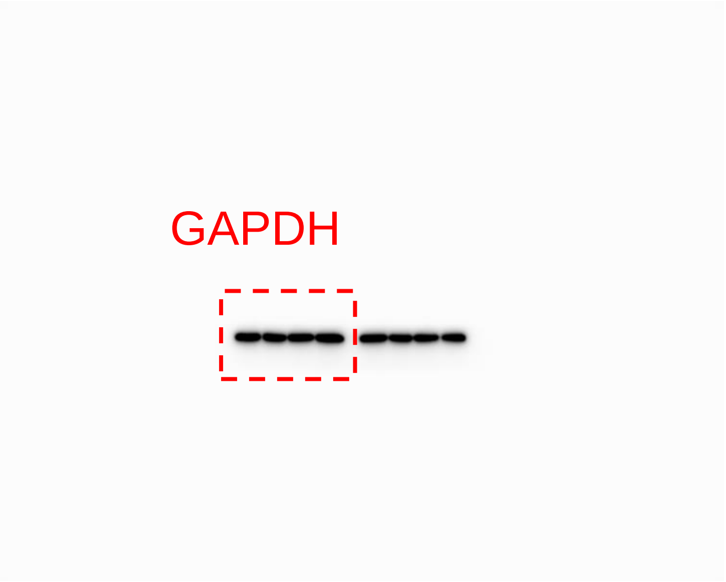

Full unedited gel for Figure.S15B

Tan Z. et al.Fig.S15

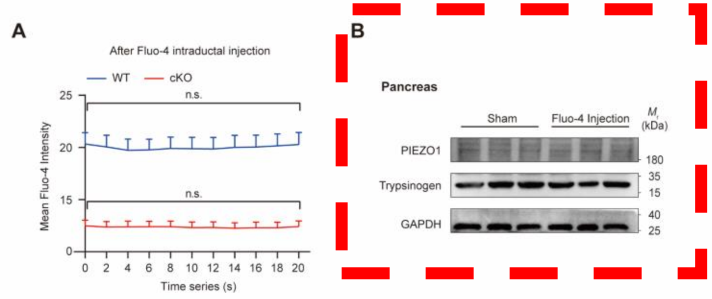

PIEZO1

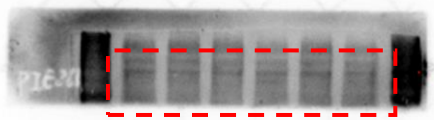

Trypsinogen

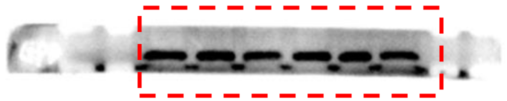

GAPDH

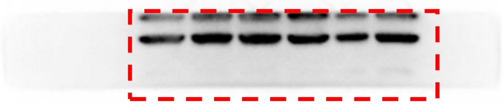

Supplement: Unedited blot and gel images [file jciinsight-9-169481-s112.pdf]
