# Supplementary material for: PFKFB3 controls acinar IP3R-mediated Ca2+ overload to regulate acute pancreatitis severity
Source: JCI Insight. 2024 May 23;9(13):e169481. doi: 10.1172/jci.insight.169481 (PMC11383365; doi:10.1172/jci.insight.169481)
Supplement: Supplemental table 1 [file jciinsight-9-169481-s113.pdf]

Table S1

Name	Chromosome	Region	Max group mean	Log₂ fold change	Fold change	P-value	FDR p-value	Bonferroni
Arid5a	1	36307733.	2.131389	-1.17048	-2.25086	0.00028	0.029608	1
Cnnm3	1	36511867.	6.659136	-1.39577	-2.63128	0.000197	0.023219	1
Mgat4a	1	compleme	4.364181	1.251382	2.380693	0.000237	0.026386	1
2010300C(	1	compleme	5.054346	1.433209	2.700467	0.000153	0.019472	1
Slc39a10	1	compleme	1.953729	-2.19946	-4.59307	1.8E-08	1.03E-05	0.000878
Sdpr	1	51289126.	4.53373	1.169552	2.249419	0.000352	0.034945	1
Kansl1l	1	compleme	3.767524	-1.91278	-3.76533	1.32E-07	6E-05	0.006424
Cyp27a1	1	74713574.	1.452132	1.67697	3.197556	0.000217	0.024883	1
Scg2	1	compleme	6.779121	1.415016	2.666627	1.86E-05	0.0038	0.904449
Neu2	1	87509889.	0.497021	2.549335	5.853645	9.44E-05	0.013441	1
Ramp1	1	91179822.	5.424292	1.342692	2.536241	4.83E-05	0.008121	1
Serpinb2	1	107511423	3.500037	-4.64869	-25.0838	7.11E-10	5.97E-07	3.46E-05
Inhbb	1	compleme	2.898846	-1.70784	-3.2667	5.88E-05	0.009413	1
Tmem37	1	compleme	0.827967	2.484977	5.598255	0.000181	0.022026	1
Pigr	1	130826684	15.49111	-2.8239	-7.08072	1.11E-16	3E-13	5.41E-12
Mapkapk2	1	compleme	40.10114	-1.33178	-2.51712	0.000294	0.030665	1
Ikbke	1	compleme	1.645444	-1.36529	-2.57628	0.00011	0.015255	1
Slc26a9	1	131744022	4.778349	-2.62293	-6.16	7.28E-13	1.12E-09	3.55E-08
Klhdc8a	1	132298626	0.497352	2.268788	4.81918	3.16E-05	0.005729	1
Nfasc	1	compleme	1.212924	1.525126	2.878118	3.01E-06	0.000853	0.146711
Btg2	1	compleme	100.0236	-1.9767	-3.93592	8E-06	0.001948	0.389667
Chil1	1	134182176	1.682272	-2.61634	-6.13192	3.64E-06	0.000997	0.177443
Elf3	1	compleme	8.562042	-1.26587	-2.40472	0.000168	0.021013	1
Shisa4	1	compleme	6.982188	-1.36525	-2.57622	0.000347	0.034552	1
Phlda3	1	135766119	7.023849	-1.73301	-3.3242	2.59E-05	0.004974	1
5730559C1	1	compleme	3.375489	-1.75112	-3.3662	0.00054	0.048948	1
Angptl1	1	156838562	1.149501	2.360216	5.134473	0.000236	0.026316	1
Selp	1	164115264	1.92513	-1.97357	-3.92738	1.62E-06	0.000501	0.078909
Pcp4l1	1	compleme	1.228999	2.412495	5.323943	5.79E-05	0.009307	1
F11r	1	171437535	30.16065	-1.18338	-2.27108	0.000378	0.03708	1
Pydc3	1	173673675	0.616589	-2.06836	-4.19411	0.000245	0.027133	1
Esrrg	1	187608791	0.12348	3.247391	9.496466	7.28E-05	0.010913	1
Il1rn	2	24336853.	2.828965	-3.23587	-9.42093	1.26E-13	2.36E-10	6.13E-09
Fcna	2	compleme	4.801112	1.795164	3.470549	9.71E-07	0.000328	0.047313
Gpsm1	2	26315515.	3.035534	2.013963	4.038903	0.000301	0.031021	1
Sardh	2	compleme	3.864286	1.071574	2.101724	0.000412	0.03962	1
Gpr107	2	31152316.	8.99368	-1.53023	-2.88833	5.53E-05	0.009045	1
Lcn2	2	compleme	511.8955	-2.5816	-5.98603	5.88E-08	2.98E-05	0.002863
Hspa5	2	34771970.	523.6762	-2.28154	-4.86197	3.67E-06	0.000997	0.178522
Kif5c	2	49619298.	0.579155	1.63435	3.104477	0.000129	0.017183	1
Lypd6	2	50066429.	0.138961	3.320224	9.988195	0.000169	0.021139	1
Nr4a2	2	compleme	0.937178	1.941619	3.841364	0.000271	0.028895	1
Gcg	2	compleme	30.78182	1.608433	3.049206	2.71E-06	0.000777	0.132061
Stk39	2	compleme	4.769763	1.34633	2.542645	4.98E-05	0.008218	1
Zak	2	72285637.	1.551539	-1.43496	-2.70375	4.26E-05	0.007337	1
Tnks1bp1	2	85048022.	24.83154	-1.79185	-3.46258	3.22E-05	0.0058	1
Creb3l1	2	compleme	5.352574	1.743945	3.349499	9.03E-08	4.4E-05	0.004401
Bmf	2	compleme	5.658582	-1.52333	-2.87454	0.000185	0.022289	1
Pak6	2	118663303	1.784956	-1.71447	-3.28177	0.000414	0.039729	1
Sord	2	122234749	18.49404	1.621794	3.077574	0.000245	0.027133	1
Duox2	2	compleme	1.284757	-2.42084	-5.35482	3.79E-06	0.001021	0.184826
Il1b	2	compleme	2.644843	-1.80394	-3.49172	0.000148	0.019008	1
Plcb1	2	134786067	0.931864	2.424199	5.367308	7.27E-12	9.3E-09	3.54E-07
Btbd3	2	138256565	3.005948	1.166766	2.245079	0.000477	0.044458	1

Pcsk2	2	143546156	1.193764	1.275406	2.42067	0.00017	0.021139	1
Acss1	2	compleme	2.257032	1.383968	2.609852	8.28E-05	0.012191	1
Myl9	2	156775420	5.030762	1.372134	2.588532	0.000408	0.039383	1
Src	2	157418444	16.23296	-1.67825	-3.20039	1.17E-05	0.002617	0.567986
Blcap	2	compleme	7.461832	-1.76385	-3.39604	2.56E-06	0.000744	0.124927
Lpin3	2	160880670	13.76371	-1.57244	-2.97408	0.000448	0.042253	1
Rims4	2	compleme	1.209241	1.997609	3.993377	5.48E-06	0.00139	0.266932
Slpi	2	compleme	14.86152	-1.37074	-2.58603	4.22E-05	0.007292	1
Sdc4	2	compleme	46.07016	-1.69837	-3.24534	0.000104	0.014553	1
Mmp9	2	164940780	3.295331	-1.40773	-2.6532	0.000383	0.037428	1
Slc13a3	2	compleme	1.106753	-3.89305	-14.8568	2.7E-11	2.99E-08	1.32E-06
Gm14434	2	compleme	0.359135	3.483906	11.1882	2.65E-05	0.005067	1
Cpb1	3	compleme	8135.455	1.737833	3.335339	5.56E-06	0.001403	0.270836
Zmat3	3	compleme	3.489515	-1.68996	-3.22647	2.66E-07	0.000108	0.012958
Mccc1	3	compleme	1.092088	1.828589	3.551895	1.97E-06	0.000599	0.095813
P2ry14	3	compleme	6.081912	1.959619	3.889593	9.37E-05	0.013387	1
P2ry12	3	compleme	0.494439	2.374661	5.18614	0.000306	0.03127	1
Ptx3	3	66219910.	7.975147	-3.6888	-12.8956	0	0	0
Bche	3	compleme	0.540984	1.502394	2.833124	0.000423	0.040349	1
Serpini2	3	compleme	296.0764	3.261038	9.586727	0	0	0
Fgg	3	83007724.	2.610068	-6.29323	-78.4244	3.54E-09	2.54E-06	0.000172
Fga	3	83026076.	2.022148	-4.76225	-27.1382	2.49E-11	2.83E-08	1.21E-06
Trim2	3	compleme	0.586044	1.258199	2.39197	0.000206	0.024004	1
Efna1	3	compleme	10.25372	-1.77906	-3.43203	6.84E-05	0.010466	1
S100a8	3	90668978.	34.03115	-2.39313	-5.25297	2.45E-12	3.32E-09	1.19E-07
S100a9	3	compleme	61.79111	-1.76551	-3.39993	3.94E-08	2.11E-05	0.001917
Gm15441	3	compleme	8.550992	2.6496	6.274933	2.77E-07	0.000111	0.013492
Fmo5	3	97628804.	0.674118	1.84937	3.603427	2.58E-05	0.004963	1
Fam46c	3	compleme	10.07288	1.800585	3.483616	2.56E-07	0.000105	0.01249
Kcnd3	3	105452330	0.761409	3.464258	11.03686	4.72E-12	6.21E-09	2.3E-07
Gstm7	3	compleme	2.128152	2.728898	6.629491	2.71E-08	1.5E-05	0.001321
Gstm2	3	compleme	14.85801	1.648409	3.134878	1.12E-06	0.000368	0.054558
Gstm1	3	compleme	23.38239	1.522511	2.872906	2.2E-05	0.004385	1
Amy2a4	3	compleme	18710.6	1.332018	2.517545	0.000271	0.028895	1
Amy2a3	3	compleme	18705.51	1.333477	2.520093	0.000256	0.027852	1
Amy2a2	3	compleme	18713.1	1.332746	2.518817	0.000264	0.028449	1
Amy2a1	3	compleme	54939.28	1.435992	2.705682	0.000123	0.016513	1
Amy1	3	compleme	57.03979	1.553354	2.934987	0.000301	0.031021	1
Dbt	3	116513070	11.89067	1.93851	3.833096	3.22E-08	1.76E-05	0.001569
A530020G	3	121531619	1.736062	-1.84336	-3.58845	0.00025	0.027325	1
Alpk1	3	compleme	1.086078	-1.72395	-3.30339	0.000189	0.022644	1
Tifa	3	127789805	14.16642	-2.75603	-6.75536	0	0	0
Egf	3	compleme	2.122881	1.860285	3.630793	4.21E-09	2.97E-06	0.000205
Gar1	3	compleme	10.94686	-1.45404	-2.73974	3.02E-05	0.005539	1
Slc39a8	3	135825279	23.1685	-2.33364	-5.04076	1.16E-08	7E-06	0.000565
Bmpr1b	3	compleme	1.628647	3.491537	11.24753	1.96E-10	1.99E-07	9.56E-06
Gbp2b	3	142594847	0.333233	3.854621	14.46627	2.36E-05	0.004661	1
Kyat3	3	142701051	2.235365	1.77388	3.419725	9.13E-06	0.00216	0.444912
Clca3a1	3	compleme	1.6311	1.315766	2.489346	0.000336	0.033609	1
Acadm	3	compleme	6.460336	1.421095	2.677886	1.42E-05	0.003078	0.693311
Cth	3	compleme	5.649865	4.502065	22.65983	0	0	0
Ggh	4	20042052.	51.80019	1.996774	3.991066	8.43E-09	5.47E-06	0.000411
Enho	4	compleme	2.415965	1.87441	3.666518	0.000261	0.028208	1
Frmpd1	4	45184875.	0.183319	3.840206	14.32244	0.000182	0.022026	1
Shb	4	compleme	9.828284	-1.16375	-2.2404	0.000362	0.035872	1
Aldob	4	compleme	3.439593	2.435767	5.41052	5.04E-06	0.001306	0.245518
Susd1	4	compleme	1.159397	-1.66277	-3.16624	0.000122	0.016461	1
Orm1	4	63344560.	3.565555	-4.13856	-17.6128	8.26E-06	0.001993	0.402579

Megf9	4	compleme	1.848255	1.892116	3.711793	6.01E-05	0.009564	1
Plin2	4	compleme	25.17905	-1.42978	-2.69405	0.000125	0.016764	1
Leprot	4	10164771{	48.76695	-1.87489	-3.66774	6.45E-09	4.49E-06	0.000314
Zc3h12a	4	compleme	3.774949	-1.6744	-3.19187	8.11E-06	0.001965	0.394922
Sh3d21	4	compleme	3.08704	-2.17744	-4.5235	7.27E-05	0.010913	1
Adprhl2	4	compleme	6.251184	-1.44112	-2.71532	5.58E-05	0.009092	1
C77080	4	compleme	37.57401	-2.03513	-4.09859	2.45E-06	0.00072	0.119531
Bsdc1	4	12946158	11.34419	-1.62619	-3.08697	1.22E-05	0.002715	0.591863
Stmn1	4	13446832(	15.03951	-2.29063	-4.89269	2.08E-12	2.9E-09	1.01E-07
Cela3b	4	compleme	8126.09	1.344903	2.540132	0.000278	0.029455	1
Padi2	4	14090634	2.192146	1.802297	3.487752	1.05E-07	4.89E-05	0.005091
Arhgef19	4	14123949	1.977967	2.075662	4.215377	4.2E-07	0.000155	0.020444
Cela2a	4	compleme	21897.43	1.508914	2.845957	4.16E-05	0.007212	1
Ctrc	4	compleme	66.84364	2.402103	5.28573	2.13E-13	3.7E-10	1.04E-08
Gm13066	4	compleme	1.448266	2.735882	6.661662	3.39E-05	0.006066	1
H6pd	4	compleme	47.12733	-2.13591	-4.39513	6.94E-07	0.000245	0.033783
Gm13648	4	compleme	2.383563	-1.93751	-3.83043	0.000257	0.02792	1
Steap4	5	7960457..7	10.4031	-1.42331	-2.682	1.84E-05	0.003787	0.897558
Gnai1	5	compleme	4.0137	1.29368	2.451526	4.93E-05	0.008201	1
Fgl2	5	21372642.	33.37258	1.755561	3.376576	6.5E-05	0.010083	1
Gm29609	5	compleme	0.405994	-3.10778	-8.62054	0.000343	0.034286	1
Qdpr	5	compleme	17.28791	1.218159	2.326497	0.000459	0.042953	1
Cckar	5	compleme	74.98566	2.316673	4.981819	1.33E-05	0.002921	0.64549
Klb	5	65348314.	2.554613	1.394004	2.628071	0.000174	0.02139	1
Gabra4	5	compleme	1.740898	1.214741	2.320991	0.000322	0.032605	1
Hopx	5	compleme	1.792459	2.106884	4.307599	0.000326	0.032888	1
Jchain	5	compleme	9.989681	1.621653	3.077273	1.29E-06	0.000413	0.062719
Cxcl5	5	90759360.	8.96824	-4.38979	-20.9633	1.15E-14	2.56E-11	5.62E-10
Cxcl1	5	90891241.	6.415283	-3.85393	-14.4593	2.07E-10	2.05E-07	1.01E-05
Cxcl2	5	90903871.	3.542672	-4.10162	-17.1677	1.27E-11	1.54E-08	6.17E-07
Shroom3	5	92683435.	4.602214	-1.48384	-2.79691	1.93E-05	0.003939	0.941317
Cxcl13	5	95956951.	21.9856	-1.24717	-2.37375	7E-05	0.010617	1
Ptpn13	5	10342519	1.826198	1.374605	2.592969	4.68E-05	0.007944	1
Gfi1	5	compleme	0.774065	1.692876	3.233007	0.000554	0.049812	1
Tmed11	5	compleme	71.30769	2.554034	5.872742	1.41E-13	2.55E-10	6.89E-09
Pla2g1b	5	11546626	743.5068	1.399329	2.637789	0.000296	0.030777	1
Aacs	5	12547581	6.325359	-2.3149	-4.97571	1.72E-11	2.04E-08	8.37E-07
Rabgef1	5	13017179{	4.139097	-1.41559	-2.66768	5.62E-05	0.009124	1
Cldn4	5	compleme	12.78836	-1.82138	-3.5342	4.11E-05	0.007142	1
Cldn3	5	13498621	74.82959	-1.57075	-2.9706	3.16E-06	0.000886	0.154155
Hspb1	5	13588791	204.9689	-2.66292	-6.33315	7.45E-12	9.3E-09	3.63E-07
Ywhag	5	compleme	19.33526	-1.28738	-2.44084	0.000228	0.025823	1
Dtx2	5	13599480(	1.740918	-1.29392	-2.45193	0.000392	0.038094	1
Azgp1	5	13798152(	7.311427	1.793473	3.466484	9.77E-07	0.000328	0.047589
Pdgfa	5	compleme	13.82343	-1.33547	-2.52358	0.000122	0.016513	1
Gpr146	5	13937769	4.139008	-1.34568	-2.5415	3.1E-06	0.000872	0.150871
Lfng	5	14060732(	5.38249	1.232001	2.348925	0.000227	0.025823	1
Bhlha15	5	14419028	20.14007	2.760588	6.776724	7.36E-13	1.12E-09	3.58E-08
Gm45062	6	8259450..8	0.757318	1.781379	3.437546	1.69E-05	0.003539	0.820948
Aass	6	compleme	3.348205	3.073219	8.416492	1.11E-16	3E-13	5.41E-12
Cpa2	6	30541582.	1420.676	1.543837	2.91569	3.05E-05	0.00554	1
Cpa1	6	30639218.	12062.32	1.365534	2.576717	0.000193	0.022989	1
Akr1b8	6	34354119.	7.044986	-1.54538	-2.91881	6.28E-05	0.009834	1
Gm4744	6	compleme	4.977414	4.560015	23.58855	2.3E-14	4.87E-11	1.12E-09
1810009J0	6	40964754.	25.29106	3.392399	10.50059	0	0	0
2210010C(	6	compleme	7367.839	1.726265	3.3087	3.22E-06	0.000895	0.15666
Try4	6	41302269.	43246.8	1.411271	2.659714	0.000192	0.022897	1
Try5	6	compleme	29083.7	1.581689	2.993201	2.95E-05	0.005466	1

Gm5771	6	41392356.	35.40767	1.746167	3.35466	1.75E-08	1.01E-05	0.00085
Gm10334	6	compleme	12.86124	1.343858	2.538291	2.94E-05	0.005466	1
Nfe2l3	6	51432670.	1.7229	-2.05649	-4.15973	9.94E-06	0.002316	0.484057
Fam13a	6	compleme	4.577866	-1.65674	-3.15304	0.000129	0.017136	1
Gadd45a	6	compleme	3.627258	-2.07751	-4.22078	4.91E-05	0.008184	1
Igkv1-110	6	68270485.	14.73534	1.860204	3.630589	3.7E-06	0.001002	0.180395
Igkv4-74	6	compleme	5.126271	4.980792	31.57679	7.34E-10	6.02E-07	3.57E-05
Igkv6-32	6	compleme	2.99768	3.183084	9.082465	5.19E-06	0.001339	0.252992
Igkv6-25	6	70215400.	1.818358	3.944358	15.39466	9.53E-05	0.013532	1
Igkv6-20	6	compleme	2.98963	3.327755	10.04047	1.13E-06	0.000368	0.054807
Igkv3-2	6	70698449.	7.538146	3.132256	8.768048	3.02E-05	0.005539	1
Atoh8	6	compleme	4.518524	-1.39859	-2.63644	0.000146	0.01886	1
Mthfd2	6	compleme	6.921859	1.527752	2.883363	2.47E-07	0.000102	0.012021
Exoc6b	6	compleme	2.649107	-1.4391	-2.71152	4.85E-06	0.001262	0.236081
Rab11fip5	6	compleme	37.08938	-1.91627	-3.77445	7.72E-06	0.001891	0.376252
Nat8	6	compleme	0.673932	4.211305	18.52376	1.24E-05	0.002749	0.602107
Clec4e	6	compleme	0.97193	-2.8567	-7.24356	5.14E-08	2.63E-05	0.002502
Ing4	6	12503976(	4.073806	-1.26683	-2.40633	7.09E-05	0.010726	1
Tnfrsf1a	6	12534936Z	36.54122	-1.50759	-2.84334	2.6E-06	0.00075	0.126685
Dusp16	6	compleme	3.855356	-1.47471	-2.77928	2.46E-05	0.004798	1
Emp1	6	13536254E	3.820276	1.186368	2.275791	0.000249	0.02728	1
Mgst1	6	13814031(	20.6444	-1.31556	-2.48898	2.04E-05	0.004115	0.995809
Iapp	6	14229842E	44.19389	1.20653	2.307819	0.000306	0.03127	1
Isoc2a	7	4877153..4	3.380643	1.552843	2.933948	4.95E-05	0.008207	1
Pnmal2	7	16944682.	2.126904	1.316158	2.49002	0.000327	0.032888	1
Cblc	7	compleme	3.180395	-1.37807	-2.59921	0.000229	0.025823	1
Irgq	7	24530689.	4.275368	-1.2008	-2.29867	0.000206	0.024004	1
Cxcl17	7	compleme	2.180703	-3.11759	-8.67936	0.000114	0.015618	1
Ceacam1	7	compleme	33.59329	-2.01663	-4.04636	4.11E-08	2.18E-05	0.002001
Itpkc	7	compleme	16.13262	-3.0081	-8.04504	1.01E-08	6.32E-06	0.000493
Coq8b	7	27233023.	7.155275	-1.62572	-3.08595	2.22E-06	0.000659	0.108148
Spred3	7	compleme	1.170903	-1.41395	-2.66466	0.000527	0.048284	1
Dpf1	7	29303951.	0.586354	2.103863	4.298588	0.000327	0.032888	1
Kirrel2	7	compleme	0.576654	2.58233	5.989063	0.000113	0.015581	1
Nphs1	7	30458315.	1.805786	1.295912	2.455322	4.07E-05	0.007109	1
Hamp2	7	compleme	18.03752	1.54874	2.925615	2.06E-05	0.004135	1
Fxyd1	7	compleme	4.218647	1.666607	3.174672	1.5E-05	0.003203	0.730222
Rhpn2	7	35334170.	7.033808	-1.33359	-2.52029	0.000116	0.015818	1
Ccne1	7	compleme	1.525292	-2.35862	-5.12881	4.49E-05	0.007673	1
Siglece	7	compleme	2.422362	-2.39682	-5.26641	1.42E-06	0.000448	0.069044
Shank1	7	44310253.	0.166254	2.532689	5.78649	1.13E-05	0.002566	0.549897
Bax	7	compleme	18.52948	-1.18473	-2.27322	0.000548	0.049364	1
Fgf21	7	compleme	2.31595	5.453045	43.80563	1.37E-06	0.000435	0.06649
Fut2	7	compleme	8.463336	-4.77862	-27.4479	0	0	0
Dbp	7	45705088.	4.336178	-1.75387	-3.37263	8.34E-06	0.002002	0.406475
Fam83e	7	45721212.	0.600333	-2.10302	-4.29608	0.000449	0.042253	1
Saa3	7	compleme	121.5246	-5.66418	-50.7095	0	0	0
Saa1	7	compleme	36.51069	-3.75944	-13.5427	0	0	0
Saa2	7	46751790.	30.83228	-5.17292	-36.0747	0	0	0
9130015G	7	compleme	0.421578	4.108035	17.24415	4.53E-06	0.001207	0.220873
Snrpn	7	compleme	2.559193	1.498442	2.825373	0.000116	0.015818	1
Aen	7	78895854.	5.661163	-1.36844	-2.58191	6.61E-06	0.001643	0.321974
Anpep	7	compleme	13.92713	1.366728	2.57885	0.000432	0.040843	1
Homer2	7	compleme	2.275875	1.298883	2.460384	0.00012	0.016253	1
Mesdc1	7	compleme	4.709346	-1.35249	-2.55352	0.000257	0.02792	1
Ddias	7	compleme	0.849972	-3.29577	-9.82031	3.09E-07	0.00012	0.015061
Dgat2	7	compleme	4.68872	-2.39054	-5.24352	1.56E-08	9.15E-06	0.00076
Pold3	7	compleme	4.583051	-1.37797	-2.59902	1.79E-05	0.003727	0.872135

Lipt2	7	100159277	1.987906	-1.88198	-3.68582	0.000426	0.040436	1
Pgm2l1	7	100227394	2.045059	-1.28792	-2.44175	0.000302	0.031021	1
Plekhb1	7	compleme	2.943508	1.777037	3.427216	1.29E-05	0.002857	0.628441
Xylt1	7	117380979	0.929021	1.81814	3.526263	3.97E-07	0.000151	0.019318
Tmc5	7	118597297	2.977139	-2.12627	-4.36586	1.41E-05	0.003078	0.687037
Nupr1	7	compleme	1410.882	-1.68297	-3.21089	1.09E-05	0.002501	0.530291
Sult1a1	7	compleme	12.84722	2.154836	4.45318	5.24E-06	0.001345	0.255472
Mvp	7	compleme	25.98068	-1.27565	-2.42108	9.98E-05	0.014017	1
Zg16	7	compleme	4833.855	2.317229	4.983742	7.41E-10	6.02E-07	3.61E-05
Tbc1d10b	7	compleme	6.395985	-1.06814	-2.09672	0.000502	0.046452	1
Zfp553	7	127233061	4.949723	-1.24974	-2.37798	0.000227	0.025823	1
Tacc2	7	130577438	5.362755	-2.54231	-5.82523	1.66E-10	1.72E-07	8.07E-06
Ctbp2	7	compleme	4.935163	-1.04022	-2.05653	0.000535	0.048698	1
Brsk2	7	141949751	1.599096	2.264702	4.80555	2.3E-07	9.65E-05	0.011199
Shank2	7	144001928	0.324702	1.648918	3.135984	0.000386	0.037568	1
Cttn	7	compleme	15.93684	-1.33577	-2.52411	8.1E-05	0.011956	1
1810010D0	7	compleme	1.980834	2.007677	4.021343	0.000144	0.018641	1
Cd209b	8	compleme	7.44601	2.254811	4.772717	7.09E-08	3.56E-05	0.003454
6430573F1	8	36457548.	0.167539	2.613071	6.118045	9.64E-05	0.013649	1
Lpl	8	68880491.	9.506629	1.731232	3.320112	6.21E-07	0.000221	0.030251
Hapln4	8	70083457.	1.843165	2.408379	5.308775	1.37E-09	1.04E-06	6.66E-05
Upf1	8	compleme	14.56792	-1.26635	-2.40553	0.000405	0.039164	1
Rab3a	8	70754679.	1.36785	1.943016	3.845085	9.99E-05	0.014017	1
Tpm4	8	72134975.	58.79642	-1.35488	-2.55777	0.000144	0.018687	1
Nr3c2	8	76899442.	0.524208	1.933768	3.820516	6.43E-06	0.001605	0.312971
Il15	8	compleme	0.257789	3.247032	9.494104	0.000161	0.020208	1
Junb	8	compleme	34.10436	-2.1522	-4.44504	5.21E-10	4.45E-07	2.54E-05
Gpt2	8	85492576.	4.084254	1.334999	2.522754	0.000326	0.032888	1
Phkb	8	85840959.	1.154604	1.282968	2.433391	0.000425	0.040436	1
Ces1d	8	compleme	5.50476	2.008338	4.023184	1.5E-07	6.68E-05	0.007284
Gfod2	8	compleme	1.971484	-1.44338	-2.71956	0.000282	0.029719	1
Ctrl	8	compleme	7873.924	1.456575	2.74456	6.42E-05	0.010025	1
Psmb10	8	compleme	12.12026	1.20606	2.307067	0.000265	0.028476	1
Lcat	8	compleme	4.045655	2.150319	4.439259	7.31E-07	0.000254	0.035591
Cdh1	8	106603351	40.22166	-1.41671	-2.66976	0.000204	0.023928	1
Tmed6	8	compleme	120.1559	2.021743	4.060741	8.27E-09	5.47E-06	0.000403
Hp	8	compleme	127.888	-2.68295	-6.42167	3.56E-07	0.000137	0.017317
Marveld3	8	compleme	4.024623	-1.2994	-2.46126	0.000139	0.018231	1
Znrf1	8	111536097	3.869722	-1.22403	-2.33598	3.55E-05	0.006302	1
Ctrb1	8	compleme	77621.84	1.393016	2.626272	0.000194	0.022998	1
Cbfa2t3	8	compleme	1.847228	1.753375	3.371464	2.38E-06	0.000702	0.115808
Trim67	8	124793092	0.18105	-2.61083	-6.10856	0.000401	0.038931	1
Mmp7	9	7692090..7	37.61508	-2.13784	-4.40102	2.96E-07	0.000117	0.014423
Muc16	9	compleme	0.031206	3.299754	9.847478	5.92E-05	0.009448	1
Icam1	9	21015960.	9.499062	-1.30887	-2.47747	0.000427	0.04049	1
Spc24	9	compleme	0.844933	2.386514	5.228924	0.000134	0.017678	1
St14	9	compleme	22.98348	-1.64032	-3.11735	2.21E-06	0.000659	0.107613
Tirap	9	compleme	4.592351	-1.45952	-2.75017	1.81E-05	0.003738	0.882226
Vwa5a	9	38718268.	8.2755	-1.07904	-2.11263	0.000304	0.031194	1
1700110K1	9	40323426.	5.663421	1.348492	2.546459	0.000373	0.036661	1
Usp2	9	44067021.	3.632497	2.662115	6.329603	1.95E-05	0.003966	0.95173
Mpzl2	9	45042425.	3.54898	1.800197	3.482678	5.12E-05	0.008429	1
Scn4b	9	45138437.	2.509584	3.54705	11.68876	6.3E-13	1.02E-09	3.07E-08
Fam81a	9	compleme	0.258749	3.645068	12.51051	1.43E-05	0.003078	0.696709
Scg3	9	compleme	2.280424	1.497653	2.82383	0.00015	0.019174	1
Ibtk	9	compleme	21.19078	-1.95931	-3.88877	2.99E-06	0.000853	0.145795
Plscr1	9	92249750.	3.33925	-1.75001	-3.36361	9.8E-06	0.002295	0.477463
Tfdp2	9	96196275.	5.333001	1.922831	3.791663	2.07E-06	0.000627	0.100914

Rbp2	9	98486115.	0.663602	3.963422	15.59943	0.000278	0.029462	1
Mapkapk3	9	compleme	4.769912	-1.3067	-2.47374	1.11E-05	0.002543	0.541645
Rassf1	9	107551555	4.208798	-1.58153	-2.99288	3.59E-06	0.00099	0.174931
Sema3f	9	compleme	5.599343	-1.30489	-2.47066	3.88E-05	0.0068	1
Ngp	9	110419747	3.870831	-2.07095	-4.20164	0.000265	0.028456	1
Dclk3	9	111439081	0.310653	4.468339	22.13625	1.51E-06	0.00047	0.073341
Myd88	9	compleme	17.39103	-1.86686	-3.64739	7.81E-09	5.36E-06	0.00038
Myrip	9	120303936	1.067821	2.003706	4.010289	5.41E-06	0.001381	0.263705
Vipr1	9	121642716	1.710889	1.785331	3.446976	2.69E-05	0.005111	1
Ccr2	9	124101950	1.386559	1.411209	2.6596	0.000139	0.018221	1
Iyd	10	3540240..3	2.053454	2.115451	4.333254	6.67E-06	0.00165	0.325073
Plagl1	10	13060504.	0.630038	1.494594	2.817848	0.000283	0.029747	1
Scml4	10	42860370.	1.157191	2.066185	4.187777	2.33E-08	1.31E-05	0.001137
Adarb1	10	compleme	1.089468	2.082777	4.236219	5.29E-05	0.008674	1
Sbno2	10	compleme	22.6828	-2.01081	-4.03008	1.38E-08	8.22E-06	0.000674
Gamt	10	compleme	9.589758	2.124634	4.360926	1.52E-09	1.14E-06	7.4E-05
Klf16	10	compleme	3.503513	-1.49187	-2.81254	0.000298	0.030914	1
Mknk2	10	compleme	39.0079	-1.4684	-2.76715	0.000246	0.027133	1
Creb3l3	10	compleme	0.585449	4.042675	16.48035	7.88E-05	0.011671	1
Aldh1l2	10	compleme	3.458866	3.985883	15.8442	0	0	0
Nuak1	10	compleme	1.867334	1.285199	2.437157	0.000404	0.039156	1
Pah	10	87521795.	23.76758	2.167796	4.493366	8.6E-08	4.23E-05	0.004188
Tbc1d30	10	compleme	1.375904	1.463531	2.757824	0.000299	0.030925	1
Slc16a7	10	compleme	1.037417	1.789746	3.45754	7.29E-07	0.000254	0.035531
Rdh7	10	compleme	0.337719	3.456646	10.97878	9.34E-05	0.013381	1
Erbb3	10	compleme	29.66811	-2.457	-5.49074	1.63E-06	0.000501	0.079196
Limk2	11	compleme	6.697035	-1.2981	-2.45905	2.3E-05	0.004562	1
Ddc	11	compleme	0.56647	2.165743	4.486976	7.8E-05	0.011584	1
Stc2	11	compleme	2.265093	2.408558	5.309435	9.21E-08	4.44E-05	0.004485
Tnip1	11	compleme	11.43864	-1.78865	-3.45491	4.37E-08	2.29E-05	0.00213
Usp22	11	compleme	10.15793	-1.34956	-2.54834	0.000471	0.043922	1
Fam83g	11	61684091.	1.974752	-1.47509	-2.78001	8.32E-05	0.012206	1
Adora2b	11	62248984.	0.921904	-2.58639	-6.00594	0.000322	0.032605	1
Mfsd6l	11	68556186.	2.210273	-2.93652	-7.65564	2.33E-08	1.31E-05	0.001135
Cldn7	11	69964779.	23.44549	-1.67874	-3.20149	2.19E-06	0.000659	0.106775
Mgl2	11	70130329.	0.388961	2.996323	7.979637	8.51E-05	0.012367	1
Clec10a	11	70156197.	1.333679	3.209811	9.252291	1.16E-08	7E-06	0.000567
Pitpnm3	11	compleme	0.130897	2.425579	5.372446	0.000171	0.02119	1
P2rx1	11	72999103.	18.99605	2.061058	4.172921	4.87E-05	0.008152	1
Traf4	11	compleme	8.894485	-1.11648	-2.16817	0.000247	0.027133	1
Vtn	11	78499091.	7.68008	1.156969	2.229885	0.000213	0.024615	1
Adap2	11	80154105.	6.279775	2.058471	4.165445	1.67E-07	7.34E-05	0.008119
Ccl8	11	82115185.	2.330811	3.061754	8.349872	1.03E-05	0.002381	0.502337
Slfn4	11	83175186.	2.590492	-1.57736	-2.98423	3.65E-05	0.006434	1
Ccl9	11	compleme	7.821686	1.10755	2.154794	0.000358	0.035511	1
Ccl6	11	compleme	27.35179	1.487094	2.803238	9.45E-06	0.002223	0.460213
Ccl3	11	compleme	2.149312	-4.03121	-16.3499	3.05E-05	0.00554	1
Wfdc21	11	83746940.	4.034492	-2.73796	-6.67126	0.00023	0.025823	1
Vmp1	11	compleme	51.03715	-1.71609	-3.28544	9.32E-05	0.013381	1
Wfikkn2	11	compleme	5.676465	-3.98067	-15.7871	0	0	0
Abcc3	11	compleme	4.698631	-1.5307	-2.88926	8.76E-07	0.000298	0.042672
Epn3	11	compleme	8.300608	-1.69787	-3.24421	7.42E-06	0.001824	0.361225
Itga3	11	compleme	7.716679	-1.51274	-2.85352	1.15E-05	0.002587	0.558891
Mllt6	11	97663414.	10.03714	-1.30628	-2.47303	0.000218	0.024885	1
Ppp1r1b	11	98348404.	2.252853	2.43721	5.415934	2.77E-07	0.000111	0.013482
Grb7	11	98446394.	8.612664	-1.58297	-2.99586	0.00023	0.025823	1
Csf3	11	98701263.	0.916744	-5.0772	-33.7589	0.000291	0.030453	1
Thra	11	98740638.	2.57133	1.210895	2.314811	0.000157	0.019802	1

Nr1d1	11	compleme	4.925576	-2.41212	-5.32254	4.19E-10	3.65E-07	2.04E-05
Rapgefl1	11	98836785.	0.76773	1.666443	3.174309	0.000512	0.047212	1
Krt20	11	compleme	37.88124	-2.28395	-4.8701	2.22E-10	2.17E-07	1.08E-05
Krt23	11	compleme	9.337054	-1.40277	-2.64408	0.000105	0.014652	1
Stat3	11	compleme	19.91205	-1.56573	-2.96027	0.000206	0.024004	1
Gprc5c	11	114851152	5.619864	-1.25323	-2.38375	7.22E-05	0.010883	1
Fads6	11	compleme	2.246355	1.581929	2.993698	4.62E-05	0.007869	1
Slc16a5	11	115462474	1.933163	1.920667	3.785981	4.54E-07	0.000164	0.022114
Myo15b	11	115858406	2.046905	1.169008	2.24857	0.000141	0.018382	1
Itgb4	11	115974709	21.17871	-1.43188	-2.69798	0.000228	0.025823	1
Rhbdf2	11	compleme	9.567343	-2.12456	-4.3607	9.43E-08	4.5E-05	0.004593
Socs3	11	compleme	21.04402	-1.68128	-3.20712	4.76E-05	0.008057	1
Pgs1	11	117986292	7.703155	-1.16406	-2.24088	0.000229	0.025823	1
Pycr1	11	compleme	2.472725	2.623852	6.163936	3.08E-10	2.89E-07	1.5E-05
Cbr2	11	compleme	3.650384	2.184178	4.544678	0.000523	0.048067	1
Sdc1	12	8771323..8	27.25768	-1.83895	-3.5775	2.49E-06	0.000725	0.12114
Mycn	12	compleme	0.501043	2.410224	5.315569	0.0002	0.023498	1
Sostdc1	12	36314169.	10.28231	3.256185	9.55453	9.97E-08	4.72E-05	0.004858
2310015A1	12	compleme	2.029342	-1.33049	-2.51488	0.00054	0.048948	1
Actn1	12	compleme	41.13758	-1.30218	-2.46601	0.000281	0.029651	1
Mfsd7c	12	85746539.	0.372316	2.485046	5.59852	6.19E-05	0.009729	1
Irf2bpl	12	compleme	7.945108	-1.44298	-2.71882	6.63E-05	0.010253	1
9030617O	12	100779057	4.711078	-1.80366	-3.49104	0.000495	0.04597	1
Ifi27	12	103434211	25.09958	1.557298	2.943022	0.000137	0.018077	1
Ifi27l2b	12	compleme	4.906353	4.738002	26.68583	0	0	0
Ppp4r4	12	103532283	1.317923	2.074668	4.212474	1.52E-05	0.00324	0.741981
Serpina3k	12	104338486	2.610179	-2.21372	-4.63869	8.92E-05	0.012929	1
Serpina3m	12	104338486	25.52162	-3.93579	-15.3036	0	0	0
Serpina3n	12	104406729	234.9179	-2.39973	-5.27703	1.21E-09	9.47E-07	5.9E-05
Tnfaip2	12	111442469	2.656489	-1.39908	-2.63733	0.000421	0.040209	1
Trmt61a	12	111678105	8.042011	-1.56001	-2.94855	0.00011	0.015255	1
Cep170b	12	112722174	24.56606	-1.36999	-2.58468	0.000546	0.049247	1
Pld4	12	112760655	2.66682	1.565229	2.959245	0.000522	0.048067	1
Crip1	12	113146316	4.361209	1.211558	2.315876	0.000368	0.036343	1
Igha	12	compleme	19.64985	1.322809	2.501527	8.4E-05	0.012255	1
Ighg2c	12	compleme	9.642915	3.199447	9.186067	0	0	0
Ighg2b	12	compleme	8.675416	1.525031	2.877928	6.19E-05	0.009729	1
Ighv2-3	12	compleme	2.492623	2.951147	7.733638	6.84E-05	0.010466	1
Ighv2-5	12	compleme	1.650985	3.932167	15.26512	1.55E-05	0.003276	0.75356
Ighv5-9-1	12	compleme	1.005635	4.228324	18.74357	0.000274	0.029137	1
Ighv14-2	12	compleme	2.681776	2.827862	7.10021	7.64E-05	0.011385	1
Ighv1-7	12	compleme	6.595325	3.051043	8.288107	7.47E-08	3.71E-05	0.003639
Ighv1-26	12	compleme	7.488662	1.785348	3.447016	0.000455	0.042681	1
Ighv1-34	12	compleme	2.749383	4.666822	25.40115	3E-07	0.000118	0.014617
Ighv1-53	12	compleme	4.797993	2.417162	5.341195	0.000318	0.032334	1
Ighv1-55	12	compleme	3.284484	2.228429	4.686234	0.000188	0.022596	1
Ighv8-8	12	compleme	3.319859	2.836138	7.141059	0.000129	0.017136	1
Ighv1-81	12	compleme	10.83588	4.651019	25.12443	9.42E-13	1.39E-09	4.59E-08
Vipr2	12	116077726	1.785999	2.781152	6.874009	1.23E-09	9.47E-07	5.97E-05
Ptprn2	12	116485720	4.474669	1.689371	3.225161	1.22E-06	0.000393	0.059333
Rapgef5	12	117516479	2.910519	1.537829	2.903572	4.62E-06	0.001223	0.225074
Akr1c14	13	4049011..4	0.297681	3.1135	8.654795	3.56E-05	0.006302	1
Edaradd	13	compleme	1.142034	1.293528	2.451268	0.000241	0.026763	1
Ero1lb	13	12565883.	43.06187	2.306473	4.946723	9.96E-10	7.95E-07	4.85E-05
Sox4	13	compleme	17.08029	-2.70127	-6.50374	3.96E-10	3.51E-07	1.93E-05
Foxq1	13	31558324.	1.071154	-2.61489	-6.12577	0.000217	0.024883	1
Eci3	13	compleme	1.444424	2.35255	5.10726	7.46E-05	0.011146	1
Gcnt2	13	40859768.	2.156561	-1.46464	-2.75994	8.4E-05	0.012255	1

Susd3	13	compleme	2.436505	2.83518	7.136319	4.13E-06	0.001104	0.200958
RP24-267I	13	compleme	1.580373	2.657658	6.310077	8.95E-05	0.012938	1
Agtpbp1	13	compleme	1.001843	1.197619	2.293608	0.000171	0.02119	1
Ctla2b	13	compleme	5.152419	-1.69824	-3.24505	0.000214	0.024628	1
Aaed1	13	compleme	30.94487	-1.14855	-2.2169	0.000418	0.04003	1
Pde8b	13	compleme	0.260481	2.379798	5.204638	0.000234	0.026209	1
Mccc2	13	compleme	2.840525	1.699859	3.248692	1.43E-05	0.003078	0.698681
Elovl7	13	108214404	2.001239	-1.29541	-2.45446	0.000485	0.045126	1
Nnt	13	compleme	1.950281	2.291174	4.894542	1.81E-11	2.1E-08	8.81E-07
Acox2	14	compleme	0.69495	-2.78186	-6.87736	6.85E-05	0.010466	1
Thrb	14	17660960.	1.380259	1.787334	3.451764	4.35E-07	0.000158	0.021201
Nr1d2	14	compleme	9.283587	-1.55039	-2.92896	0.000212	0.024558	1
Adk	14	21052574.	45.72802	1.407798	2.653319	0.000145	0.018761	1
Plac9a	14	compleme	1.143942	-3.93694	-15.3157	0.000113	0.015581	1
Tmem254c	14	compleme	5.342504	-1.60078	-3.03307	4.34E-05	0.007443	1
Plac9b	14	compleme	5.517756	-1.55942	-2.94736	0.000175	0.021422	1
Itih4	14	30886476.	4.154165	1.29706	2.457276	0.000288	0.0302	1
Mat1a	14	41105381.	33.64335	2.009451	4.026291	3.6E-06	0.00099	0.175176
Sftpd	14	compleme	1.989989	-3.3776	-10.3934	4.66E-06	0.001228	0.227162
Pnp	14	50944302.	19.21452	-1.34033	-2.5321	0.000125	0.016763	1
Rnase1	14	compleme	6889.277	2.446289	5.450123	3.78E-08	2.05E-05	0.001843
Tgm1	14	compleme	1.981903	-2.58131	-5.98484	4.63E-08	2.4E-05	0.002256
Gata4	14	compleme	6.047651	1.51039	2.848869	2.46E-05	0.004798	1
Reep4	14	70545251.	8.159776	-2.17226	-4.5073	4.08E-07	0.000154	0.019894
Acod1	14	103046977	0.896121	-5.86946	-58.4632	2.48E-05	0.004803	1
Cldn10	14	118787908	6.18984	1.653822	3.146662	2.45E-05	0.004798	1
Pcca	14	122534324	2.197127	1.934392	3.82217	3.83E-07	0.000147	0.018678
Oxct1	15	4026383..4	3.057162	1.11689	2.16879	0.000314	0.031998	1
Fam134b	15	25843264.	48.34319	2.163406	4.479712	2.47E-05	0.004798	1
Slc39a4	15	compleme	2.630548	-1.87725	-3.67375	2.96E-05	0.005466	1
Foxred2	15	compleme	3.811517	2.18908	4.560145	1.16E-08	7E-06	0.000563
Lgals1	15	78926725.	64.07474	1.579359	2.988371	4.83E-05	0.008121	1
Triobp	15	78947724.	4.072104	-1.65187	-3.1424	5.38E-07	0.000193	0.026184
Gcat	15	79030874.	24.09428	1.613382	3.059683	6.39E-06	0.001605	0.311464
Sun2	15	compleme	14.68368	-1.58819	-3.00673	2.91E-05	0.005463	1
1810041L1	15	compleme	1.260695	1.738942	3.337904	2.21E-05	0.004385	1
Gtse1	15	85859707.	2.121815	-3.65279	-12.5776	8.53E-09	5.47E-06	0.000416
Cpne8	15	compleme	6.190722	-1.14076	-2.20496	0.000194	0.022998	1
Rnd1	15	compleme	1.434548	-1.62938	-3.0938	0.000542	0.049049	1
Fkbp11	15	compleme	19.27359	2.161448	4.473637	1.46E-10	1.54E-07	7.1E-06
Faim2	15	compleme	1.92908	-2.26534	-4.80769	4.26E-07	0.000156	0.020767
Abat	16	8513429..8	0.806005	2.799945	6.964137	3.95E-10	3.51E-07	1.93E-05
Litaf	16	compleme	35.39678	-1.15652	-2.22919	0.000211	0.02451	1
Mpv17l	16	13903161.	0.98652	2.122643	4.354909	1.2E-06	0.00039	0.058517
Ypel1	16	17069696.	2.023848	-3.73602	-13.3246	3.11E-15	7.21E-12	1.51E-10
Ccdc116	16	compleme	0.554106	-2.6771	-6.3957	5.67E-05	0.009166	1
Dgcr8	16	compleme	2.808131	-1.38715	-2.61562	0.000269	0.028814	1
Camk2n2	16	compleme	8.201058	-2.16465	-4.48357	8.54E-07	0.000295	0.041606
Ehhadh	16	compleme	6.827321	1.292595	2.449682	3.82E-05	0.006726	1
Sst	16	compleme	8.58678	1.737602	3.334805	2.95E-05	0.005466	1
Bdh1	16	31422280.	0.891807	1.800125	3.482504	0.000196	0.023203	1
B4galt4	16	38742264.	4.938523	1.974785	3.930697	1.76E-09	1.3E-06	8.58E-05
Nepro	16	44724301.	2.812798	-1.30558	-2.47183	0.000527	0.048284	1
Retnlg	16	48872608.	11.45208	-2.41573	-5.33589	1.67E-07	7.34E-05	0.008148
RP23-299I	16	compleme	1.456004	-2.78933	-6.9131	2.35E-07	9.78E-05	0.011441
Cxadr	16	78301496.	12.99248	-1.88933	-3.70463	1.16E-07	5.31E-05	0.005629
Cldn8	16	compleme	3.998822	2.097712	4.280299	2.15E-07	9.16E-05	0.010457
1600012H(	17	14943184.	11.14909	-1.29253	-2.44957	0.000157	0.019802	1

Fpr1	17	compleme	4.616332	-4.54647	-23.3681	2.03E-12	2.9E-09	9.89E-08
Fpr2	17	17887824.	3.358508	-3.5271	-11.5282	8.36E-09	5.47E-06	0.000407
Eci1	17	24426683.	7.536562	1.437884	2.709232	0.000107	0.014934	1
Hs3st6	17	24753003.	1.142629	3.271023	9.653308	1.47E-06	0.000463	0.071763
Mettl26	17	25875464.	13.84936	1.640123	3.116924	2.86E-05	0.005422	1
Rab40c	17	compleme	8.187952	-1.38575	-2.61308	0.000296	0.030777	1
Arhgdig	17	compleme	19.75697	1.757313	3.380678	8.69E-07	0.000298	0.042349
Rgs11	17	26202951.	7.782505	3.162261	8.952315	8.89E-14	1.73E-10	4.33E-09
Scube3	17	28142316.	0.053493	4.317398	19.9373	0.00053	0.048399	1
Clps	17	compleme	11380.15	2.911059	7.521701	1.11E-15	2.85E-12	5.41E-11
4930539E(	17	compleme	8.012522	-5.10737	-34.4724	0	0	0
Glp1r	17	30901867.	4.899769	1.92202	3.789533	6.82E-05	0.010466	1
Tff2	17	compleme	545.6543	1.434434	2.70276	0.000154	0.019588	1
Cbs	17	compleme	16.9909	1.400141	2.639274	2.89E-05	0.005453	1
B3galt4	17	compleme	8.261128	-1.44857	-2.72938	0.000259	0.028079	1
Clic1	17	35049966.	42.13021	-1.21763	-2.32565	0.000254	0.02779	1
Tnf	17	compleme	0.915282	-2.92041	-7.57064	0.000503	0.046454	1
Ddr1	17	compleme	22.3208	-1.50225	-2.83284	6.48E-05	0.01008	1
Ier3	17	35821684.	13.53665	-1.52833	-2.88452	2.92E-05	0.005463	1
Flot1	17	35823230.	25.91174	-1.55181	-2.93185	1.02E-05	0.002369	0.497503
Gm26917	17	39843013.	30.07065	2.456623	5.489303	4.47E-11	4.84E-08	2.18E-06
Gtpbp2	17	46161032.	7.379776	-1.19795	-2.29414	0.000206	0.024004	1
Cul7	17	46650337.	6.358897	-1.33677	-2.52585	0.000176	0.021539	1
Gnmt	17	compleme	45.55385	1.612672	3.058177	3.53E-05	0.006302	1
Trem2	17	48346401.	0.208631	3.06764	8.384008	1.76E-05	0.003674	0.85614
Trip10	17	57249451.	10.75952	-1.23729	-2.35756	0.000147	0.018875	1
Tgif1	17	compleme	7.642936	-1.8918	-3.71097	1.86E-06	0.00057	0.090573
Ypel5	17	72836704.	18.86246	-1.66743	-3.17649	0.000371	0.03657	1
Epcam	17	87635979.	47.54016	-1.28578	-2.43814	0.000182	0.022026	1
Arhgap12	18	compleme	8.339878	-1.4108	-2.65885	0.000247	0.027133	1
Ttr	18	20665250.	4.222141	1.840376	3.581034	1.81E-05	0.003738	0.881434
Cd14	18	compleme	26.68284	-2.47971	-5.57786	6.51E-14	1.32E-10	3.17E-09
Fchsd1	18	compleme	1.360743	-1.64872	-3.13555	0.000142	0.018439	1
Pcdh1	18	compleme	4.103107	-1.69066	-3.22805	4.16E-07	0.000155	0.020286
Tnfaip8	18	49979427.	3.892628	-1.64197	-3.12092	1.02E-06	0.00034	0.049666
Ptpn2	18	compleme	9.006176	-2.06556	-4.18596	3.85E-10	3.51E-07	1.87E-05
Rab27b	18	compleme	0.720686	2.589584	6.019252	2.77E-09	2.01E-06	0.000135
Galr1	18	compleme	0.483253	5.526976	46.10897	1.94E-07	8.46E-05	0.009472
Gal	19	compleme	35.36669	2.593257	6.034594	0	0	0
Ssh3	19	compleme	10.08165	-1.15026	-2.21954	0.000329	0.032965	1
Syt12	19	compleme	1.792504	-2.36492	-5.15126	7.97E-09	5.39E-06	0.000388
Rela	19	5637483..5	39.31264	-1.92313	-3.79245	1.07E-07	4.98E-05	0.005225
Fam89b	19	compleme	18.81074	-1.28892	-2.44345	6.88E-05	0.010474	1
Neat1	19	compleme	160.9953	-2.03142	-4.08806	1.37E-05	0.003015	0.669367
Atg2a	19	6241668..6	20.94556	-2.13646	-4.39681	4.73E-06	0.001239	0.230446
Lgals12	19	compleme	0.556669	2.571593	5.944657	0.000154	0.019588	1
Fads2	19	compleme	4.613349	1.674516	3.192123	1.67E-05	0.003514	0.811625
Fads1	19	10182888.	3.578782	1.303345	2.468005	5.68E-05	0.009166	1
Dagla	19	compleme	1.981149	1.444587	2.721848	1.97E-05	0.003989	0.961355
Stx3	19	compleme	3.633681	-1.35477	-2.55757	0.000185	0.022277	1
Gcnt1	19	compleme	0.859547	1.571872	2.972903	0.000173	0.021232	1
Aldh1a7	19	compleme	7.362309	1.859668	3.629242	4.2E-07	0.000155	0.020434
Ric1	19	29522282.	3.868942	1.601797	3.035212	0.000157	0.019802	1
Uhrf2	19	30030513.	2.949071	-1.09436	-2.13518	0.000449	0.042253	1
A1cf	19	31868761.	0.829502	2.44558	5.447446	0.000471	0.043922	1
Cpeb3	19	compleme	0.753216	1.702565	3.25479	9.9E-05	0.013972	1
Aldh18a1	19	compleme	6.369762	1.597092	3.02533	8.58E-06	0.002049	0.417967
Cnnm1	19	43440436.	0.298841	2.741389	6.687138	3.23E-05	0.0058	1

Entpd7	19	43689672.	1.373581	1.218607	2.327219	0.000418	0.04003	1
Cpn1	19	compleme	3.398971	1.967426	3.910697	9.54E-09	6.03E-06	0.000465
Scd2	19	44293676.	5.476156	1.783157	3.441786	1.47E-07	6.63E-05	0.007161
Kcnip2	19	compleme	0.622752	3.092352	8.528856	2.07E-07	8.91E-05	0.010063
Sfxn2	19	46573365.	3.245017	1.668447	3.178723	1.13E-05	0.002566	0.551619
1810018F1	19	58698917.	16.80354	2.610328	6.106425	0.000531	0.048399	1
Pnliprp1	19	58728887.	8554.408	1.322809	2.501528	0.000373	0.036661	1
Pnliprp2	19	58759723.	2050.375	1.50138	2.831134	6.07E-05	0.009612	1
Ccdc120	X	compleme	2.482285	-1.77727	-3.42776	1.42E-05	0.003078	0.692755
Slc38a5	X	8271133..8	6.395936	2.847956	7.199795	1.33E-15	3.24E-12	6.49E-11
Gpr34	X	13632089.	0.443299	2.867376	7.297367	0.000545	0.049247	1
Smarca1	X	compleme	1.613072	4.457252	21.96679	2.75E-10	2.63E-07	1.34E-05
Ocrl	X	47912387.	2.116495	2.056875	4.160841	6.08E-05	0.009612	1
Ddx26b	X	56454857.	1.62961	1.222157	2.332953	0.00017	0.021139	1
Fhl1	X	56731787.	1.705264	1.40727	2.652348	0.00012	0.016253	1
F8a	X	73228291.	5.64023	-1.40286	-2.64425	0.000179	0.021786	1
Slc10a3	X	compleme	5.571869	-1.89046	-3.70754	1.03E-06	0.000341	0.050163
F8	X	compleme	0.691744	1.533843	2.895561	9.16E-05	0.013205	1
Eda2r	X	compleme	2.180474	-3.19192	-9.13828	2.84E-13	4.78E-10	1.39E-08
Eda	X	99975606.	1.535993	1.302135	2.465936	0.000383	0.037428	1
Cox7b	X	10601570(	17.75227	1.323961	2.503526	8.73E-06	0.002075	0.425314
Prdx4	X	compleme	24.3487	1.251486	2.380865	0.000132	0.01748	1
Grpr	X	compleme	0.842234	2.098357	4.282213	0.000172	0.02119	1
Mid1	X	16968519(	2.387124	-1.96425	-3.9021	2.16E-07	9.16E-05	0.010538

ENSEMBL
ENSMUSG00000037447
ENSMUSG00000001138
ENSMUSG00000026110
ENSMUSG00000026090
ENSMUSG00000025986
ENSMUSG00000045954
ENSMUSG00000026004
ENSMUSG00000026170
ENSMUSG00000050711
ENSMUSG00000079434
ENSMUSG00000034353
ENSMUSG00000062345
ENSMUSG00000037035
ENSMUSG00000050777
ENSMUSG00000026417
ENSMUSG00000016528
ENSMUSG00000042349
ENSMUSG00000042268
ENSMUSG00000042115
ENSMUSG00000026442
ENSMUSG00000020423
ENSMUSG00000064246
ENSMUSG00000003051
ENSMUSG00000041889
ENSMUSG00000041801
ENSMUSG00000041605
ENSMUSG00000033544
ENSMUSG00000026580
ENSMUSG00000038370
ENSMUSG00000038235
ENSMUSG00000066677
ENSMUSG00000026610
ENSMUSG00000026981
ENSMUSG00000026938
ENSMUSG00000026930
ENSMUSG00000009614
ENSMUSG00000000194
ENSMUSG00000026822
ENSMUSG00000026864
ENSMUSG00000026764
ENSMUSG00000050447
ENSMUSG00000026826
ENSMUSG00000000394
ENSMUSG00000027030
ENSMUSG00000004085
ENSMUSG00000033955
ENSMUSG00000027230
ENSMUSG00000040093
ENSMUSG00000074923
ENSMUSG00000027227
ENSMUSG00000068452
ENSMUSG00000027398
ENSMUSG00000051177
ENSMUSG00000062098

RPKM	2-wt-ctr-1	3-wt-ctr-2	5-cko-ctr-1	6-cko-ctr-2	7-wt-faee-1
Arid5a	0.265407	0.536908	0.175426	0.248175	1.930915
Cnnm3	1.730692	0.868896	0.729101	1.667521	6.730823
Mgat4a	2.380558	2.938378	2.217039	2.523384	3.980962
2010300C[illegible]	5.151617	4.512848	3.851544	3.708927	4.897091
Slc39a10	0.156087	0.264114	0.178166	0.182891	2.189007
Sdpr	4.659571	5.341166	3.675947	4.024138	3.625116
Kansl1l	0.407128	0.543516	0.540528	0.382342	2.957351
Cyp27a1	1.070188	1.060702	0.658186	1.143225	1.083511
Scg2	4.006857	2.938501	3.443692	3.406521	4.126087
Neu2	0.897883	0.466151	0.664704	0.381205	0.235683
Ramp1	5.445004	5.932176	3.575858	5.912419	4.847795
Serpinb2	0.015	0.404951	0.270609	0.054234	6.051095
Inhbb	0.107651	0.309539	0.298783	0.217068	1.936697
Tmem37	0.268765	0.139534	0.181825	0.412993	0.133032
Pigr	0.533998	0.908964	2.566335	0.585548	16.24893
Mapkapk2	5.932384	5.257478	3.597064	6.04305	38.74078
Ikbke	0.199267	0.316444	0.149787	0.113015	1.851774
Slc26a9	0.013485	0.007001	0	0.024377	3.696015
Klhdc8a	0.453954	0.385655	0.483931	0.283501	0.153202
Nfasc	0.154442	0.108632	0.082388	0.1396	0.952478
Btg2	9.826783	12.01742	8.487458	28.89987	68.79563
Chil1	0.201056	0.195716	0.06612	0.022717	1.088477
Elf3	0.731085	0.660095	0.62123	0.818853	8.928727
Shisa4	0.670312	0.650616	0.449099	0.302948	9.556942
Phlda3	0.700634	0.821362	0.645583	0.367725	9.229267
5730559C[illegible]	0.155751	0.095563	0.117076	0.076791	1.357886
Angptl1	1.227834	1.725623	0.894983	0.986525	0.736664
Selp	0.031938	0.190681	0.150045	0.057737	1.995799
Pcp4l1	0.804353	1.090382	0.369492	0.929016	0.691206
F11r	4.154648	3.970444	3.212521	3.659713	31.32155
Pydc3	0.160922	0.410763	0.045361	0.036364	0.779928
Esrrg	0.192976	0.12376	0.046931	0.061564	0.04214
Il1rn	0.022601	0.058669	0.042473	0.010215	2.670894
Fcna	1.872026	2.423076	2.351731	3.500153	2.665581
Gpsm1	3.141902	1.529222	1.116904	3.260048	1.133974
Sardh	2.815177	3.158825	3.066941	2.732266	3.002004
Gpr107	1.442865	1.228882	0.884144	1.45609	7.426594
Lcn2	0.617924	0.878728	0.555366	0.837814	523.1491
Hspa5	42.16716	55.68757	51.04906	41.73743	233.1107
Kif5c	0.217074	0.143434	0.252176	0.218511	0.451764
Lypd6	0.170081	0.117734	0.092335	0.076868	0.023385
Nr4a2	0.989604	0.676012	0.456766	2.503044	0.537094
Gcg	14.8346	15.01144	14.96258	15.43422	20.02041
Stk39	4.956383	4.289878	3.308732	5.17427	4.258936
Zak	0.225089	0.233718	0.20564	0.222239	1.84261
Tnks1bp1	2.820405	2.680039	2.184315	2.240354	16.86518
Creb3l1	3.221651	3.612197	3.113593	3.352534	3.299829
Bmf	0.784791	0.724333	0.357986	0.964021	3.585715
Pak6	0.182565	0.189564	0.177894	0.085567	0.962224
Sord	11.17576	19.78619	17.12799	9.24869	17.31446
Duox2	0.044023	0.131419	0.08273	0.029845	2.001996
Il1b	0	0.095925	0.055555	0.033402	2.743658
Plcb1	0.694241	0.576025	0.602873	0.636122	0.341279
Btbd3	2.934227	2.495637	2.444075	4.918521	2.136282

ENSMUSG00000027419	Pcsk2	0.766175	0.72154	0.582623	0.40667	0.959118
ENSMUSG00000027452	Acss1	2.543621	2.463869	1.610455	3.155589	1.72166
ENSMUSG00000067818	Myl9	2.941314	3.935649	3.168278	2.877928	4.27758
ENSMUSG00000027646	Src	2.410272	1.849796	1.545162	2.23694	12.96767
ENSMUSG00000067787	Blcap	1.12317	1.166225	0.792053	0.921038	5.129564
ENSMUSG00000027412	Lpin3	1.211386	1.218206	1.039646	1.457091	9.159341
ENSMUSG00000035226	Rims4	1.36339	0.862664	0.683231	0.815162	0.685389
ENSMUSG00000017002	Slpi	2.149939	3.689584	4.758482	3.697732	14.5584
ENSMUSG00000017009	Sdc4	6.612637	5.860621	3.930768	5.689568	29.70092
ENSMUSG00000017737	Mmp9	0.139387	0.330812	0.269424	0.197988	3.018457
ENSMUSG00000018459	Slc13a3	0.013513	0.007015	0.015236	0.018322	1.387884
ENSMUSG00000078881	Gm14434	0	0.052755	0.009548	0.103332	0.125743
ENSMUSG00000011463	Cpb1	9887.26	9683.96	10147.43	9958.936	3790.166
ENSMUSG00000027663	Zmat3	0.613958	0.628451	0.448414	0.425074	4.375217
ENSMUSG00000027709	Mccc1	1.145334	1.099044	1.085844	1.227201	0.796225
ENSMUSG00000036381	P2ry14	6.671239	4.022464	4.570363	7.289603	4.976661
ENSMUSG00000036353	P2ry12	0.207591	0.344878	0.093626	0.137604	0.085627
ENSMUSG00000027832	Ptx3	0	0	0.041864	0.01678	10.06184
ENSMUSG00000027792	Bche	0.53838	0.792394	0.664014	0.193712	0.342808
ENSMUSG00000034139	Serpini2	344.1355	415.9786	560.7463	288.5152	50.30681
ENSMUSG00000033860	Fgg	0.050504	0	0.075927	0	1.791549
ENSMUSG00000028001	Fga	0.081942	0	0.12319	0	1.274722
ENSMUSG00000027993	Trim2	0.52912	0.541323	0.355329	0.411455	0.517544
ENSMUSG00000027954	Efna1	0.808675	0.779697	0.853917	0.783174	5.289338
ENSMUSG00000056054	S100a8	0.101542	0.263586	0.610626	0.22946	37.56999
ENSMUSG00000056071	S100a9	0.472502	0.841053	0.862567	0.305068	67.07249
ENSMUSG00000074398	Gm15441	6.201886	14.66924	10.17716	2.204351	2.939706
ENSMUSG00000028088	Fmo5	0.658136	0.431938	0.462045	0.875499	0.35342
ENSMUSG00000044468	Fam46c	10.29834	11.04714	10.02181	9.752702	4.433788
ENSMUSG00000040896	Kcnd3	0.911475	0.507553	0.345336	0.880359	0.118247
ENSMUSG00000004035	Gstm7	2.508934	2.148073	1.356554	2.188256	0.653611
ENSMUSG00000040562	Gstm2	29.86276	23.09764	15.901	18.53279	8.335924
ENSMUSG00000058135	Gstm1	27.48483	28.61292	21.84681	26.48684	18.08581
ENSMUSG00000096770	Amy2a4	21138.18	20921.57	24867.47	21130	13150.39
ENSMUSG00000093931	Amy2a3	21135.34	20911.76	24843.84	21107.57	13131.86
ENSMUSG00000096569	Amy2a2	21165.14	20917.75	24873.49	21146.03	13135.45
ENSMUSG00000070360	Amy2a1	59766.88	57328.53	61282.69	63739.66	33264.31
ENSMUSG00000074264	Amy1	40.23511	25.85212	24.60282	49.81617	41.18463
ENSMUSG00000000340	Dbt	8.682489	8.173886	8.401438	7.343932	6.745959
ENSMUSG00000097124	A530020G	0.289501	0.213328	0.280794	0.32077	2.160997
ENSMUSG00000028028	Alpk1	0.128258	0.165137	0.061703	0.097384	1.491891
ENSMUSG00000046688	Tifa	1.068398	0.754818	0.447089	0.696916	15.83767
ENSMUSG00000028017	Egf	3.116031	3.16253	3.025716	3.02951	0.986987
ENSMUSG00000028010	Gar1	1.891267	2.219908	1.298025	1.18923	8.852539
ENSMUSG00000053897	Slc39a8	0.988459	0.85927	0.8225	0.664908	17.37452
ENSMUSG00000052430	Bmpr1b	1.282459	1.221163	0.750774	1.431659	0.255962
ENSMUSG00000040264	Gbp2b	0.099852	0.336957	0.112586	0.078974	0.046335
ENSMUSG00000040213	Kyat3	1.763681	1.047593	1.314163	1.357502	1.267535
ENSMUSG00000056025	Clca3a1	0.605477	0.703753	0.210583	0.4411	1.386653
ENSMUSG00000062908	Acadm	8.734492	7.507894	4.886995	7.325369	5.549053
ENSMUSG00000028179	Cth	1.780062	4.378235	5.807726	2.715901	0.390553
ENSMUSG00000073987	Ggh	77.44554	72.96677	93.19259	65.88625	23.94188
ENSMUSG00000028445	Enho	0.805215	1.239708	0.772244	2.133311	1.614863
ENSMUSG00000035615	Frmpd1	0.205163	0.020616	0.029849	0.143572	0.032758
ENSMUSG00000044813	Shb	1.905494	1.532509	1.415782	2.488987	9.118245
ENSMUSG00000028307	Aldob	3.181767	1.085949	2.269903	4.074338	1.184818
ENSMUSG00000038578	Susd1	0.088716	0.253322	0.177832	0.113603	1.152699
ENSMUSG00000039196	Orm1	0.181859	0	0.034175	0	1.181454

ENSMUSG00000039270	Megf9	1.439217	1.000705	0.505505	3.310352	0.710257
ENSMUSG00000028494	Plin2	1.063475	1.906378	1.530933	1.0701	19.66528
ENSMUSG00000035212	Leprot	3.471532	3.816642	3.056064	3.188264	46.0823
ENSMUSG00000042677	Zc3h12a	0.366778	0.520478	0.220563	0.386786	3.343472
ENSMUSG00000073758	Sh3d21	0.518736	0.26931	0.204712	0.199277	5.038943
ENSMUSG00000042558	Adprhl2	1.025022	1.138225	0.877512	0.965123	5.90159
ENSMUSG00000050390	C77080	4.940245	3.144983	1.960048	4.266783	32.45538
ENSMUSG00000040859	Bsdc1	1.814953	2.046985	1.496006	2.409894	9.974861
ENSMUSG00000028832	Stmn1	1.191614	1.427645	0.757921	1.507943	15.65291
ENSMUSG00000023433	Cela3b	7576.799	7495.428	7061.345	7989.661	5616.711
ENSMUSG00000028927	Padi2	2.382746	3.028746	2.599453	1.188066	1.113299
ENSMUSG00000028919	Arhgef19	1.204406	1.233326	0.78671	1.201285	0.719493
ENSMUSG00000058579	Cela2a	27547.18	28103.7	26889.51	27955.5	13204.06
ENSMUSG00000062478	Ctrc	75.6272	62.18047	73.61519	59.72817	23.93797
ENSMUSG00000086949	Gm13066	1.315338	2.110719	1.288341	0.576454	0.641196
ENSMUSG00000028980	H6pd	2.981123	2.948664	1.694571	2.688558	30.39433
ENSMUSG00000086549	Gm13648	0.435851	0.738385	0.413842	0.373231	2.639922
ENSMUSG00000012428	Steap4	1.027458	1.217594	0.747146	0.999385	11.45657
ENSMUSG00000057614	Gnai1	3.450457	3.967837	3.286447	2.600754	3.282267
ENSMUSG00000039899	Fgl2	25.84963	12.39165	9.278452	28.43547	15.59656
ENSMUSG00000101678	Gm29609	0.059552	0.1752	0	0	0.528128
ENSMUSG00000015806	Qdpr	14.66159	15.61239	13.79103	17.84831	16.21637
ENSMUSG00000029193	Cckar	52.25937	52.81191	37.00975	42.09925	48.07747
ENSMUSG00000029195	Klb	2.538123	2.449388	1.915364	2.492145	1.884463
ENSMUSG00000029211	Gabra4	1.587115	2.135123	1.654552	1.685912	1.605571
ENSMUSG00000059325	Hopx	1.510741	0.675727	0.559069	1.512621	0.603976
ENSMUSG00000067149	Jchain	2.771773	5.901403	4.598464	5.099395	6.669249
ENSMUSG00000029371	Cxcl5	0	0	0	0.017096	13.69123
ENSMUSG00000029380	Cxcl1	0.110919	0.03839	0.083376	0	9.241828
ENSMUSG00000058427	Cxcl2	0	0	0.053615	0.032236	2.449263
ENSMUSG00000029381	Shroom3	1.029608	0.948805	0.689296	0.79979	5.219733
ENSMUSG00000023078	Cxcl13	2.175386	2.926863	3.592868	3.240297	24.72004
ENSMUSG00000034573	Ptpn13	1.182074	1.091346	0.680641	2.155379	1.635679
ENSMUSG00000029275	Gfi1	0.748991	0.890412	0.742519	0.588707	0.416402
ENSMUSG00000004821	Tmed11	95.91526	84.63161	107.9916	80.72885	20.91491
ENSMUSG00000029522	Pla2g1b	827.1605	797.1905	778.0462	889.764	496.3087
ENSMUSG00000029482	Aacs	0.457124	0.55059	0.432956	0.454515	5.464304
ENSMUSG00000025340	Rabgef1	0.532585	0.754889	0.641813	0.542535	3.274282
ENSMUSG00000047501	Cldn4	0.485065	0.523805	0.437542	0.263071	7.803095
ENSMUSG00000070473	Cldn3	12.23014	10.57762	8.183488	10.5489	70.61401
ENSMUSG00000004951	Hspb1	7.621175	6.387432	4.392907	18.84378	231.9623
ENSMUSG00000051391	Ywhag	3.11991	3.359241	2.687114	3.20228	16.41787
ENSMUSG00000004947	Dtx2	0.240513	0.426626	0.214689	0.158521	1.64931
ENSMUSG00000037053	Azgp1	5.799569	6.203677	5.001062	7.062186	3.060215
ENSMUSG00000025856	Pdgfa	3.840956	3.225743	1.570981	2.583461	16.39767
ENSMUSG00000044197	Gpr146	0.653373	0.597009	0.322184	0.680354	4.087824
ENSMUSG00000029570	Lfng	3.010106	4.17706	2.230947	2.504698	3.864067
ENSMUSG00000052271	Bhlha15	20.59248	25.92628	24.57563	12.08697	7.711964
ENSMUSG00000107705	Gm45062	0.868514	0.551104	0.176814	0.392525	0.477659
ENSMUSG00000029695	Aass	3.387815	3.316396	3.032312	3.387667	0.925257
ENSMUSG00000071553	Cpa2	1981.087	1814.71	1524.679	1779.413	914.3512
ENSMUSG00000054446	Cpa1	13379.74	13967.26	12878.24	14592.15	8579.34
ENSMUSG00000029762	Akr1b8	0.16454	0.268475	0.229698	0.169975	7.795295
ENSMUSG00000063252	Gm4744	3.959502	6.975321	1.203904	16.72881	0.220209
ENSMUSG00000094808	1810009J0	11.58524	9.747914	7.327162	45.78756	3.954766
ENSMUSG00000029882	2210010C0	10850.87	10027.77	8087.631	9475.1	4003.605
ENSMUSG00000054106	Try4	46173.4	39347.45	41864.34	49138.43	28022.51
ENSMUSG00000036938	Try5	24612.36	18728.52	21322.58	29170.75	19101.62

ENSMUSG00000058119	Gm5771	29.23014	19.75999	192.8359	33.60516	21.74601
ENSMUSG00000071517	Gm10334	8.486998	5.930573	31.62585	10.50729	9.904861
ENSMUSG00000029832	Nfe2l3	0.201479	0.295344	0.338535	0.160692	1.83322
ENSMUSG00000037709	Fam13a	0.924309	0.630191	0.841287	0.412709	2.308223
ENSMUSG00000036390	Gadd45a	0.21972	0.456285	0.25525	0.198605	2.37286
ENSMUSG00000093861	Igkv1-110	2.196703	5.565419	1.056794	1.667907	6.088959
ENSMUSG00000076543	Igkv4-74	0	0.371429	0.8739	0.565847	0.553316
ENSMUSG00000076576	Igkv6-32	0.192542	1.099573	0.506561	1.740387	0.238258
ENSMUSG00000094930	Igkv6-25	0.185448	0.962784	0.209099	0.50288	0.344221
ENSMUSG00000076587	Igkv6-20	0.281881	0.195124	0.211887	1.443825	0.46508
ENSMUSG00000095351	Igkv3-2	0.279644	2.226119	2.732671	1.85365	0.922779
ENSMUSG00000037621	Atoh8	0.379477	0.42321	0.443721	0.673315	5.078412
ENSMUSG00000005667	Mthfd2	6.568061	8.561515	8.636208	5.023513	4.48892
ENSMUSG00000033769	Exoc6b	0.371659	0.359742	0.277004	0.370106	2.295626
ENSMUSG00000051343	Rab11fip5	3.728341	2.312002	1.448853	5.471134	31.24143
ENSMUSG00000030004	Nat8	0.847149	0.586415	0.754718	0.198525	0.077651
ENSMUSG00000030142	Clec4e	0.007097	0.066319	0.037342	0.019244	1.027463
ENSMUSG00000030330	Ing4	0.933863	0.921522	1.025581	0.796227	4.433891
ENSMUSG00000030341	Tnfrsf1a	4.188505	4.109018	3.005346	4.228515	32.64628
ENSMUSG00000030203	Dusp16	0.668735	0.919571	0.754023	0.812768	2.784489
ENSMUSG00000030208	Emp1	0.606061	1.443034	1.24889	0.500593	2.637801
ENSMUSG00000008540	Mgst1	2.275492	3.01701	2.486753	1.708743	22.56957
ENSMUSG00000041681	Iapp	25.93797	24.94144	21.78152	16.70932	37.37893
ENSMUSG00000086784	Isoc2a	3.297404	3.130335	2.986154	2.838598	2.681363
ENSMUSG00000070802	Pnmal2	2.090147	1.235112	1.334833	1.658886	1.671716
ENSMUSG00000040525	Cblc	0.540799	0.561529	0.448207	0.363488	3.140117
ENSMUSG00000041037	Irgq	1.036838	1.064554	0.718424	0.942435	3.591032
ENSMUSG00000060188	Cxcl17	0	0	0	0	1.833432
ENSMUSG00000074272	Ceacam1	2.239366	2.24329	1.505398	1.530467	33.77861
ENSMUSG00000003752	Itpkc	0.802556	0.702801	0.603274	0.891494	6.580876
ENSMUSG00000003762	Coq8b	0.562824	0.815723	0.511209	0.582928	6.181082
ENSMUSG00000037239	Spred3	0.062333	0.088258	0.038336	0.046099	1.423458
ENSMUSG00000030584	Dpf1	0.299533	0.196976	0.232662	0.324898	0.284168
ENSMUSG00000036915	Kirrel2	0.286246	0.352947	0.262237	0.307252	0.309936
ENSMUSG00000006649	Nphs1	1.649983	1.65244	2.148478	1.698297	1.178947
ENSMUSG00000056978	Hamp2	25.95944	26.43743	29.9955	23.29597	15.02165
ENSMUSG00000036570	Fxyd1	4.803324	4.51883	4.301231	4.691401	3.171366
ENSMUSG00000030494	Rhpn2	1.406863	1.130936	0.975652	0.787612	5.233953
ENSMUSG00000002068	Ccne1	0.185309	0.054975	0.029849	0.107679	1.490503
ENSMUSG00000030474	Siglece	0.076225	0.118721	0.085947	0.03445	2.499588
ENSMUSG00000038738	Shank1	0.315018	0.205304	0.221681	0.19084	0.066352
ENSMUSG00000003873	Bax	2.302803	3.041078	2.067109	2.445265	24.87155
ENSMUSG00000030827	Fgf21	0.185253	3.270023	4.790298	0.669802	0.183391
ENSMUSG00000055978	Fut2	0	0.030087	0.029042	0.008731	7.266883
ENSMUSG00000059824	Dbp	0.51799	0.586742	0.522107	0.372442	5.448341
ENSMUSG00000054161	Fam83e	0.018337	0.00952	0.03446	0.03315	0.612665
ENSMUSG00000040026	Saa3	1.162786	4.057288	3.140499	2.517617	114.5412
ENSMUSG00000074115	Saa1	0.057857	0.180226	0	0	26.99128
ENSMUSG00000057465	Saa2	0.216166	0	0	0.048848	20.26257
ENSMUSG00000108950	9130015G	0.429495	0.291589	0.285596	0.313563	0.081765
ENSMUSG00000102252	Snrpn	1.925773	2.287671	1.864685	1.991489	2.20127
ENSMUSG00000030609	Aen	1.075093	1.154146	0.894886	1.224331	5.426674
ENSMUSG00000039062	Anpep	9.049503	12.91784	9.479835	5.807546	13.92922
ENSMUSG00000025813	Homer2	1.820244	2.632113	2.459509	1.577187	2.1488
ENSMUSG00000070462	Mesdc1	0.874321	1.221452	0.836459	0.847776	3.363781
ENSMUSG00000030641	Ddias	0.047589	0.019765	0.007154	0.060222	0.883333
ENSMUSG00000030747	Dgat2	0.205719	0.391609	0.463911	0.134297	4.652879
ENSMUSG00000030726	Pold3	0.704549	0.771315	0.535359	0.609154	4.520282

ENSMUSG00000030725	Lipt2	0.423754	0.329998	0.254825	0.36388	1.809074
ENSMUSG00000030729	Pgm2l1	0.547849	0.320694	0.334425	0.442025	1.792644
ENSMUSG00000030701	Plekhb1	2.82792	3.093977	2.289785	2.736293	2.348575
ENSMUSG00000030657	Xylt1	0.927336	0.856739	0.680603	0.653416	0.569243
ENSMUSG00000030650	Tmc5	0.047295	0.016369	0.065178	0	1.472885
ENSMUSG00000030717	Nupr1	186.421	177.8064	194.409	219.6623	1430.917
ENSMUSG00000030711	Sult1a1	15.29115	7.113229	3.638075	25.61452	5.005325
ENSMUSG00000030681	Mvp	2.513325	3.580415	2.017016	2.502271	27.35974
ENSMUSG00000049350	Zg16	5258.835	4974.241	5056.129	5245.545	2132.128
ENSMUSG00000042492	Tbc1d10b	1.620695	1.682822	1.17878	1.824404	6.601474
ENSMUSG00000045598	Zfp553	1.034176	0.856886	0.808789	1.057544	4.214059
ENSMUSG00000030852	Tacc2	0.564263	0.613018	0.43397	0.646993	3.539696
ENSMUSG00000030970	Ctbp2	1.325566	0.941114	0.719633	1.259626	4.80876
ENSMUSG00000053046	Brsk2	2.172965	1.806194	1.200396	1.077445	0.33876
ENSMUSG00000037541	Shank2	0.259351	0.365961	0.237441	0.15929	0.271557
ENSMUSG00000031078	Cttn	1.731497	2.36516	1.598504	1.876603	16.20487
ENSMUSG00000109305	1810010D0	3.280891	4.8094	5.139672	2.242893	0.887037
ENSMUSG00000065987	Cd209b	2.648881	3.632632	1.427608	1.942568	3.690133
ENSMUSG00000039620	6430573F1	0.209634	0.131748	0.07879	0.099731	0.047786
ENSMUSG00000015568	Lpl	10.14627	10.04095	7.93021	9.246053	4.609829
ENSMUSG00000007594	Hapln4	0.520805	0.606034	1.120453	0.57627	0.866692
ENSMUSG00000058301	Upf1	2.815452	3.16105	2.253996	2.110398	12.27408
ENSMUSG00000031840	Rab3a	1.092194	0.838219	0.928077	1.230467	0.822666
ENSMUSG00000031799	Tpm4	4.526345	6.593081	4.304107	5.826227	58.82118
ENSMUSG00000031618	Nr3c2	0.364651	0.502956	0.286378	0.226299	0.269393
ENSMUSG00000031712	Il15	0.124671	0.242719	0.046857	0.056345	0.077136
ENSMUSG00000052837	Junb	1.225037	1.733095	1.139549	2.588343	29.74969
ENSMUSG00000031700	Gpt2	4.907816	3.280597	2.802069	3.059046	2.967021
ENSMUSG00000036879	Phkb	0.831353	1.119721	0.92131	0.66558	1.087534
ENSMUSG00000056973	Ces1d	6.812024	7.212142	5.09817	3.818125	2.539881
ENSMUSG00000013150	Gfod2	0.550549	0.468438	0.402346	0.483818	1.835645
ENSMUSG00000031896	Ctrl	10484.4	10551.84	9461.099	10324.97	5290.919
ENSMUSG00000031897	Psmb10	7.377676	13.94766	12.76915	5.678662	11.63554
ENSMUSG00000035237	Lcat	6.773965	3.653126	3.335406	4.817707	1.949387
ENSMUSG00000000303	Cdh1	5.538708	5.343093	4.47029	4.581699	32.89639
ENSMUSG00000031919	Tmed6	148.6267	168.8155	153.5634	143.4488	56.41637
ENSMUSG00000031722	Hp	2.106209	3.234858	6.01952	2.062456	100.0712
ENSMUSG00000001672	Marveld3	1.27679	0.802124	0.935555	1.192886	3.83702
ENSMUSG00000033545	Znrf1	0.697874	1.026988	0.437783	0.567275	3.684179
ENSMUSG00000031957	Ctrb1	60215.09	57331.56	56470.6	68954.66	59054.63
ENSMUSG00000006362	Cbfa2t3	1.716939	1.638561	1.195708	0.578175	1.327879
ENSMUSG00000036913	Trim67	0	0.008368	0.003029	0.007285	0.194471
ENSMUSG00000018623	Mmp7	0.857784	0.513845	0.371993	0.954279	31.59869
ENSMUSG00000109564	Muc16	0.038779	0.065257	0.081418	0.059226	0.006619
ENSMUSG00000037405	Icam1	0.971047	2.794348	0.834201	0.827574	10.88316
ENSMUSG00000074476	Spc24	0.705225	0.705138	0.647913	0.259703	0.355533
ENSMUSG00000031995	St14	2.317188	2.350059	1.498765	2.595522	24.57753
ENSMUSG00000032041	Tirap	0.471803	0.556692	0.472866	0.639695	3.8568
ENSMUSG00000023186	Vwa5a	1.424077	1.42639	0.97315	1.332733	8.535138
ENSMUSG00000087047	1700110K1	6.615154	4.911322	2.705271	2.261656	3.30827
ENSMUSG00000032010	Usp2	3.137223	1.811404	1.305836	2.06055	1.21543
ENSMUSG00000032092	Mpzl2	1.599019	1.024449	0.912131	1.752878	2.301452
ENSMUSG00000046480	Scn4b	2.467885	1.177559	0.879289	1.431274	0.582527
ENSMUSG00000032224	Fam81a	0.274203	0.427071	0.2282	0	0.048473
ENSMUSG00000032181	Scg3	1.269028	0.976055	0.989246	1.047946	1.531571
ENSMUSG00000035941	Ibtk	1.498759	2.192264	1.762042	1.812459	14.01831
ENSMUSG00000032369	Plscr1	0.171739	0.326924	0.311978	0.245789	2.833553
ENSMUSG00000032411	Tfdp2	8.517746	5.190292	3.523659	8.044602	2.79862

ENSMUSG00000032454	Rbp2	1.025554	0.190155	4.212413	3.01275	0.135971
ENSMUSG00000032577	Mapkapk3	0.921886	1.003436	0.592611	1.097649	5.302648
ENSMUSG00000010067	Rassf1	0.729418	0.625933	0.530171	0.686567	3.595522
ENSMUSG00000034684	Sema3f	0.305095	0.584303	0.473963	0.361573	5.998639
ENSMUSG00000032484	Ngp	0.115336	0.329332	0.086697	0.052126	2.56898
ENSMUSG00000032500	Dclk3	0.23451	0.182625	0.149837	0.08479	0.01451
ENSMUSG00000032508	Myd88	1.504605	1.562281	0.86367	1.434197	16.26586
ENSMUSG00000041794	Myrip	1.690508	1.661156	1.051646	0.954301	0.368687
ENSMUSG00000032528	Vipr1	0.683931	1.670164	1.432828	0.69262	1.026558
ENSMUSG00000049103	Ccr2	0.209438	0.366258	0.443296	0.169383	1.138967
ENSMUSG00000019762	Iyd	1.910469	1.870992	1.729824	1.511016	0.725345
ENSMUSG00000019817	Plagl1	0.590331	0.959213	0.538593	0.35845	0.507446
ENSMUSG00000044770	Scml4	1.039949	1.056133	0.548575	0.766852	0.524909
ENSMUSG00000020262	Adarb1	0.62518	0.324572	0.303112	0.686597	0.425492
ENSMUSG00000035673	Sbno2	0.991952	1.488705	0.764438	0.979508	17.90675
ENSMUSG00000020150	Gamt	12.78615	19.07832	16.69125	4.455342	4.831169
ENSMUSG00000035397	Klf16	0.803793	0.793559	0.455629	0.762282	2.804571
ENSMUSG00000020190	Mknk2	5.866484	5.010335	2.524734	6.129228	36.8012
ENSMUSG00000035041	Creb3l3	0.070096	0.509484	0.658635	0.274561	0
ENSMUSG00000020256	Aldh1l2	2.892821	4.934669	6.527044	2.495969	0.261503
ENSMUSG00000020032	Nuak1	1.547213	1.378878	0.90876	1.528756	1.433998
ENSMUSG00000020051	Pah	19.69068	17.1044	18.73627	25.2651	14.08474
ENSMUSG00000052302	Tbc1d30	1.269232	1.157168	0.753947	0.94579	0.965342
ENSMUSG00000020102	Slc16a7	1.017857	1.192847	0.915004	0.852788	0.710791
ENSMUSG00000040134	Rdh7	0.335973	0.348851	0.378821	0.208784	0.051968
ENSMUSG00000018166	Erbb3	2.301762	1.179794	1.052215	2.271152	19.59739
ENSMUSG00000020451	Limk2	1.42119	1.223725	0.803392	0.976517	6.619905
ENSMUSG00000020182	Ddc	0.797746	1.664854	1.181505	0.692526	0.273669
ENSMUSG00000020303	Stc2	0.497036	1.336177	1.253921	0.492353	0.791985
ENSMUSG00000020400	Tnip1	1.16488	1.829806	0.78582	0.854948	13.42905
ENSMUSG00000042506	Usp22	2.221125	2.11907	1.593707	2.066344	7.710094
ENSMUSG00000042377	Fam83g	0.31219	0.214273	0.270467	0.330017	1.519072
ENSMUSG00000018500	Adora2b	0.019098	0	0.100488	0.069049	0.850757
ENSMUSG00000048329	Mfsd6l	0.188149	0.0888	0.180001	0.309214	2.137733
ENSMUSG00000018569	Cldn7	2.459947	2.422776	1.87631	3.041031	25.80713
ENSMUSG00000040950	Mgl2	0.134614	0.50668	0.442696	0.167307	0.10411
ENSMUSG00000000318	Clec10a	0.437552	0.769829	0.438538	0.615229	0.135361
ENSMUSG00000040543	Pitpnm3	0.068209	0.104371	0.051272	0.051919	0.022212
ENSMUSG00000020787	P2rx1	14.49673	13.51932	10.70534	10.13104	14.33445
ENSMUSG00000017386	Traf4	1.669708	1.889303	1.343604	1.451203	9.681819
ENSMUSG00000017344	Vtn	10.32754	11.53444	7.880531	7.860289	7.152331
ENSMUSG00000020709	Adap2	4.895412	3.750812	3.279205	4.175173	3.053312
ENSMUSG00000009185	Ccl8	0.689533	0.930754	0.881135	0.498616	0.341302
ENSMUSG00000000204	Slfn4	0.017755	0.165922	0.086751	0	2.526656
ENSMUSG00000019122	Ccl9	0.729002	1.153444	1.374309	1.213298	6.88743
ENSMUSG00000018927	Ccl6	2.257403	4.035647	5.99068	4.116426	18.60646
ENSMUSG00000000982	Ccl3	0.045999	0	0	0	2.106068
ENSMUSG00000051748	Wfdc21	0	0	0.122055	0	4.621336
ENSMUSG00000018171	Vmp1	6.678433	5.475553	4.780079	8.370485	31.20507
ENSMUSG00000044177	Wfikkn2	0.156519	0.191198	0.048446	0.141477	7.371319
ENSMUSG00000020865	Abcc3	0.432864	0.667764	0.283545	0.301834	5.249296
ENSMUSG00000010080	Epn3	1.429504	0.899577	0.46238	0.994549	5.714161
ENSMUSG00000001507	Itga3	1.548864	1.287871	0.955533	1.439065	7.20834
ENSMUSG00000038437	Mllt6	1.990872	2.366709	1.425425	1.765356	8.701534
ENSMUSG00000061718	Ppp1r1b	1.648638	1.974233	1.383996	0.978967	1.221075
ENSMUSG00000019312	Grb7	1.696312	1.163352	0.944519	1.410256	7.152427
ENSMUSG00000038067	Csf3	0	0	0	0	0.709717
ENSMUSG00000058756	Thra	1.672408	1.879244	1.222691	1.463373	2.211249

ENSMUSG00000020889	Nr1d1	0.492398	0.749075	0.611147	0.7556	3.995959
ENSMUSG00000038020	Rapgefl1	0.496532	0.457564	0.354576	0.185136	0.652829
ENSMUSG00000035775	Krt20	0.018285	0	0.673487	0	31.20197
ENSMUSG00000006777	Krt23	1.148237	2.407883	1.929324	0.895437	8.85956
ENSMUSG00000004040	Stat3	2.281943	2.058359	1.056433	2.48053	13.75269
ENSMUSG00000051043	Gprc5c	0.678503	0.94865	0.888754	0.564721	5.004381
ENSMUSG00000044788	Fads6	2.568621	1.523262	0.959432	1.823914	1.933321
ENSMUSG00000045775	Slc16a5	1.990846	1.750658	0.880715	1.635935	0.78975
ENSMUSG00000034427	Myo15b	2.344957	1.280521	0.85447	0.907943	1.689733
ENSMUSG00000020758	Itgb4	3.686754	2.910174	1.721677	3.005572	25.446
ENSMUSG00000020806	Rhbdf2	0.765547	0.760332	0.375296	0.661893	6.655932
ENSMUSG00000053113	Socs3	0.609393	1.063025	0.659628	1.277929	25.26191
ENSMUSG00000017715	Pgs1	1.308186	1.30345	0.735029	1.206359	7.464463
ENSMUSG00000025140	Pycr1	2.453475	3.895666	4.624093	1.885854	0.808868
ENSMUSG00000025150	Cbr2	0.770021	0.992531	1.057839	0.816028	2.759992
ENSMUSG00000020592	Sdc1	1.221065	1.325502	1.258584	1.153891	37.51154
ENSMUSG00000037169	Mycn	0.39602	0.477882	0.152865	0.251542	0.211914
ENSMUSG00000036169	Sostdc1	10.38339	12.62011	11.54125	5.620425	3.311806
ENSMUSG00000097729	2310015A1	0.53111	0.695002	0.470326	0.322239	1.971646
ENSMUSG00000015143	Actn1	4.444903	5.6345	4.510606	4.377678	48.36822
ENSMUSG00000034258	Mfsd7c	0.184638	0.266272	0.13108	0.157623	0.152319
ENSMUSG00000034168	Irf2bpl	1.225346	1.726081	1.159402	1.611043	5.545593
ENSMUSG00000021185	9030617O	0.588047	0.660095	0.579416	0.632097	7.886387
ENSMUSG00000064215	Ifi27	10.20786	12.27008	10.1105	8.619007	19.91334
ENSMUSG00000021208	Ifi27l2b	0.096711	0.435147	0.945059	0.233113	0.518588
ENSMUSG00000021209	Ppp4r4	0.882559	0.733112	0.600229	0.919308	0.603262
ENSMUSG00000058207	Serpina3k	0.27407	0	0.206016	0.077416	1.71692
ENSMUSG00000079012	Serpina3m	0.16398	0.042566	0.046223	0.259387	20.77339
ENSMUSG00000021091	Serpina3n	6.060304	4.590949	1.475716	6.450055	201.7405
ENSMUSG00000021281	Tnfaip2	0.19795	0.389054	0.180682	0.223659	3.25435
ENSMUSG00000060950	Trmt61a	1.593039	1.518769	1.306332	1.845754	5.214952
ENSMUSG00000072825	Cep170b	4.397759	3.541804	2.619878	4.507397	22.62777
ENSMUSG00000052160	Pld4	1.246946	2.312044	1.044439	0.949998	2.4027
ENSMUSG00000006360	Crip1	1.597012	3.4561	2.451461	1.519517	3.557167
ENSMUSG00000095079	Igha	5.176974	6.266756	3.861698	6.32684	13.13922
ENSMUSG00000076612	Ighg2c	0.21258	1.958968	2.456836	5.356217	1.841382
ENSMUSG00000076613	Ighg2b	1.315725	2.677675	2.383532	5.518286	6.838142
ENSMUSG00000094164	Ighv2-3	0	2.741298	0.152656	0.550704	0.376956
ENSMUSG00000096498	Ighv2-5	0	0	0.220716	0.132704	0.363344
ENSMUSG00000095210	Ighv5-9-1	0.085315	0.265756	0.384783	0.539815	0
ENSMUSG00000095583	Ighv14-2	0.100385	0.833864	0.45275	0.272214	0.496881
ENSMUSG00000095200	Ighv1-7	1.543807	0.754346	0	0.574597	1.348494
ENSMUSG00000094546	Ighv1-26	1.907314	4.273554	2.33921	4.355429	3.478166
ENSMUSG00000093955	Ighv1-34	0	0.188587	0.477838	3.693836	0.112374
ENSMUSG00000093894	Ighv1-53	0.903465	3.43969	3.320168	0.725905	1.739083
ENSMUSG00000095589	Ighv1-55	0.254712	0.528951	1.084964	0.613958	1.365824
ENSMUSG00000104452	Ighv8-8	0.492111	1.737314	0.813812	2.668917	0.730748
ENSMUSG00000094689	Ighv1-81	0.100385	1.667728	0.45275	4.627643	1.366422
ENSMUSG00000011171	Vipr2	1.713202	1.385391	1.080088	2.412052	0.762021
ENSMUSG00000056553	Ptprn2	3.264635	3.836362	3.420812	3.432236	2.666157
ENSMUSG00000041992	Rapgef5	2.288913	2.982288	2.411432	2.092916	1.77161
ENSMUSG00000033715	Akr1c14	0.343699	0.553771	0.320717	0.117841	0.073329
ENSMUSG00000095105	Edaradd	2.04375	1.526101	1.278043	0.915813	1.092319
ENSMUSG00000057069	Ero1lb	55.49303	60.46974	53.24683	52.4604	18.88326
ENSMUSG00000076431	Sox4	1.161032	1.602297	0.778834	0.943184	9.502264
ENSMUSG00000038415	Foxq1	0.119531	0.055161	0.099834	0.07203	0.427303
ENSMUSG00000021416	Eci3	1.192726	1.548059	1.344841	1.123028	0.79946
ENSMUSG00000021360	Gcnt2	0.165212	0.322296	0.259666	0.334872	1.709855

ENSMUSG00000021384	Susd3	1.726359	0.851132	0.989603	1.032822	0.676227
ENSMUSG00000110086	RP24-267I	1.402633	1.205297	1.654232	0.67764	0.688284
ENSMUSG00000021557	Agtpbp1	0.952746	1.163173	0.93907	0.977833	0.91735
ENSMUSG00000074874	Ctla2b	0.153597	0.382763	0.207823	0.194371	3.915374
ENSMUSG00000021482	Aaed1	5.220018	4.403845	3.923835	10.15763	26.10695
ENSMUSG00000021684	Pde8b	0.203775	0.448065	0.373929	0.124601	0.111247
ENSMUSG00000021646	Mccc2	2.041413	2.75729	2.021062	1.935248	2.218059
ENSMUSG00000021696	Elovl7	0.350082	0.242335	0.160816	0.32816	2.125914
ENSMUSG00000025453	Nnt	1.186688	1.388149	2.574442	2.869449	0.842657
ENSMUSG00000021751	Acox2	0	0	0.007153	0	0.400336
ENSMUSG00000021779	Thrb	1.187942	1.211154	0.86064	1.117511	0.844754
ENSMUSG00000021775	Nr1d2	2.254597	1.68261	1.535874	1.751349	7.759479
ENSMUSG00000039197	Adk	47.60005	29.2522	28.80582	47.19243	34.07805
ENSMUSG00000095304	Plac9a	0.132963	0.938805	0.059968	0.312481	0.559413
ENSMUSG00000072680	Tmem254c	1.781801	1.410704	0.435293	0.865683	5.898026
ENSMUSG00000072674	Plac9b	1.566616	2.568425	0.909032	0.447179	5.985825
ENSMUSG00000021922	Itih4	1.985825	2.337609	0.774301	1.065475	3.837124
ENSMUSG00000037798	Mat1a	22.50522	12.31691	11.68252	86.84036	12.12355
ENSMUSG00000021795	Sftpd	0.10629	0.110364	0.059923	0.192151	1.4468
ENSMUSG00000021871	Pnp	2.135808	2.06351	1.828685	3.066183	17.75145
ENSMUSG00000035896	Rnase1	7848.932	8595.322	7278.568	7938.464	3537.099
ENSMUSG00000022218	Tgm1	0.175824	0.182564	0.08811	0.042381	1.76958
ENSMUSG00000021944	Gata4	4.729304	4.123398	3.005443	6.004334	5.036933
ENSMUSG00000033589	Reep4	0.802719	0.833489	0.476365	0.553731	5.097274
ENSMUSG00000022126	Acod1	0	0.083688	0.010098	0	1.163588
ENSMUSG00000022132	Cldn10	5.53481	5.139802	3.874239	4.978339	5.065222
ENSMUSG00000041650	Pcca	2.711954	3.152979	3.029864	2.280174	1.481272
ENSMUSG00000022186	Oxct1	2.151618	2.560313	2.748063	2.848431	2.780304
ENSMUSG00000022270	Fam134b	54.95878	29.36923	31.00575	76.32927	15.37643
ENSMUSG00000063354	Slc39a4	0.126745	0.230306	0.262001	0.128886	3.077969
ENSMUSG00000016552	Foxred2	4.526755	2.094489	1.859676	5.08384	1.438938
ENSMUSG00000068220	Lgals1	15.1511	26.75336	29.26691	11.50548	53.8476
ENSMUSG00000033088	Triobp	0.696059	0.739868	0.424032	0.465168	3.412666
ENSMUSG00000006378	Gcat	23.28574	22.90531	18.34273	23.26446	14.07333
ENSMUSG00000042524	Sun2	2.127296	2.532806	1.17265	2.217706	11.33686
ENSMUSG00000062760	1810041L1	1.005796	0.865638	0.638799	0.549373	0.93845
ENSMUSG00000022385	Gtse1	0.064747	0.067229	0.048669	0.081934	3.300943
ENSMUSG00000052560	Cpne8	1.125425	1.238856	0.826889	0.703678	7.204057
ENSMUSG00000054855	Rnd1	0.249389	0.147971	0.071415	0.311299	1.778145
ENSMUSG00000003355	Fkbp11	17.97907	22.52203	24.23948	13.02719	6.859301
ENSMUSG00000023011	Faim2	0.007795	0.048565	0.046878	0.028185	2.66238
ENSMUSG00000057880	Abat	0.400192	0.373035	0.403373	0.452168	0.191332
ENSMUSG00000022500	Litaf	6.241497	5.81606	4.150325	5.002788	34.75561
ENSMUSG00000022679	Mpv17l	0.946475	1.002217	1.056618	0.694581	0.463844
ENSMUSG00000022773	Ypel1	0.167243	0.086827	0.045715	0.054971	2.426998
ENSMUSG00000022768	Ccdc116	0	0.050568	0.018304	0.014674	0.411807
ENSMUSG00000022718	Dgcr8	0.502944	0.632694	0.461667	0.498325	1.986773
ENSMUSG00000051146	Camk2n2	1.03713	0.708478	0.902695	1.134824	6.112963
ENSMUSG00000022853	Ehhadh	4.763396	7.600825	5.370896	5.825278	5.374315
ENSMUSG00000004366	Sst	6.117617	4.703015	5.836623	4.625826	4.87694
ENSMUSG00000046598	Bdh1	0.435803	0.13479	0.230009	0.402304	0.504857
ENSMUSG00000022793	B4galt4	4.2998	7.083067	4.254165	9.874054	2.660369
ENSMUSG00000036208	Nepro	0.65057	0.414227	0.438279	0.515932	2.248038
ENSMUSG00000022651	Retnlg	0.23181	0.481392	0.56631	0.052383	10.39834
ENSMUSG00000109841	RP23-299I	0.035699	0.148271	0.107339	0.032269	1.251641
ENSMUSG00000022865	Cxadr	1.485583	1.936861	1.435754	0.949061	11.47912
ENSMUSG00000050520	Cldn8	3.24534	4.099596	2.877923	1.81145	2.313312
ENSMUSG00000050088	1600012H(	2.813247	2.121398	1.511642	2.44621	12.13794

ENSMUSG00000045551	Fpr1	0	0	0.060013	0.072166	5.334902
ENSMUSG00000052270	Fpr2	0.027188	0	0.06131	0	2.994225
ENSMUSG00000024132	Eci1	5.328868	9.087763	7.258736	6.509939	6.434288
ENSMUSG00000039628	Hs3st6	0.607004	1.050453	0.521461	0.731562	0.286145
ENSMUSG00000025731	Mettl26	8.546661	12.00832	11.4398	9.505901	11.75544
ENSMUSG00000025730	Rab40c	1.104656	1.407146	0.873161	1.04997	6.496509
ENSMUSG00000073433	Arhgdig	33.16565	21.62297	13.34882	19.69511	10.27017
ENSMUSG00000024186	Rgs11	14.73575	7.793834	4.458395	8.980743	2.529414
ENSMUSG00000038677	Scube3	0.016275	0.038022	0.015292	0.025744	0.005035
ENSMUSG00000024225	Clps	15117.85	16883.88	13416.93	15403.89	3011.264
ENSMUSG00000048905	4930539E(	0.015346	0	0.034607	0.013872	6.143301
ENSMUSG00000024027	Glp1r	3.999662	4.326022	2.594901	4.411527	3.594161
ENSMUSG00000024028	Tff2	758.4601	927.7379	740.4711	639.3266	373.0001
ENSMUSG00000024039	Cbs	16.75855	17.70739	17.19246	17.63465	11.42529
ENSMUSG00000067370	B3galt4	2.043592	1.515664	1.671194	2.171989	7.91993
ENSMUSG00000007041	Clic1	4.44601	5.612939	3.327299	4.124977	40.78987
ENSMUSG00000024401	Tnf	0.085992	0.11161	0.129278	0	1.197107
ENSMUSG00000003534	Ddr1	4.649796	2.528972	1.686575	3.996145	22.82355
ENSMUSG00000003541	Ier3	0.591925	0.808704	0.327854	1.464325	14.0326
ENSMUSG00000059714	Flot1	1.839095	2.592495	1.766947	1.959604	32.32796
ENSMUSG00000097971	Gm26917	13.58316	17.51403	13.41058	25.65518	14.39997
ENSMUSG00000023952	Gtpbp2	1.700319	1.765497	1.328039	1.542924	6.53403
ENSMUSG00000038545	Cul7	1.407503	1.214589	0.657679	1.392591	5.984146
ENSMUSG00000002769	Gnmt	51.71324	38.0116	39.48725	50.09759	25.05448
ENSMUSG00000023992	Trem2	0.056992	0.051779	0.05355	0.070833	0.035262
ENSMUSG00000019487	Trip10	1.72329	2.396449	1.492	1.766302	9.824572
ENSMUSG00000047407	Tgif1	0.967018	1.082074	0.691611	1.128678	5.204738
ENSMUSG00000039770	Ypel5	2.419421	2.295379	2.317169	2.120311	9.711129
ENSMUSG00000045394	Epcam	4.041675	4.358013	3.271786	3.94991	49.35068
ENSMUSG00000041225	Arhgap12	1.552447	1.430063	1.571081	1.212154	5.942573
ENSMUSG00000061808	Ttr	5.058364	6.512811	3.954415	4.31099	1.716868
ENSMUSG00000051439	Cd14	0.739669	1.23349	0.960366	1.681599	28.92885
ENSMUSG00000038524	Fchsd1	0.302586	0.193859	0.179057	0.232772	0.963961
ENSMUSG00000051375	Pcdh1	0.412552	0.548974	0.484737	0.543067	3.429817
ENSMUSG00000062210	Tnfaip8	0.474214	0.804122	0.674457	0.579747	3.415322
ENSMUSG00000024539	Ptpn2	0.94712	1.311235	0.839073	0.805145	7.380827
ENSMUSG00000024511	Rab27b	0.6302	1.226492	1.250013	0.331047	0.265389
ENSMUSG00000024553	Galr1	0.466365	0.089203	0.202957	0.377175	0.015187
ENSMUSG00000024907	Gal	47.80495	50.92207	45.12273	50.18913	9.981757
ENSMUSG00000034616	Ssh3	2.456171	2.136396	1.295295	1.58083	10.53433
ENSMUSG00000049303	Syt12	0.076335	0.118892	0.023909	0.05175	1.550723
ENSMUSG00000024927	Rela	4.397903	4.04114	2.867029	4.467783	30.77455
ENSMUSG00000024939	Fam89b	4.896195	4.841792	2.256449	3.793438	16.98611
ENSMUSG00000092274	Neat1	25.46453	30.27705	28.32862	40.98819	206.8594
ENSMUSG00000024773	Atg2a	2.629008	1.536099	0.998428	2.939405	14.28817
ENSMUSG00000024972	Lgals12	0.343075	0.686066	1.34674	0.252679	0.2673
ENSMUSG00000024665	Fads2	2.570201	3.663431	1.86174	1.499525	3.469599
ENSMUSG00000010663	Fads1	1.743404	1.76789	1.754997	1.290182	3.242342
ENSMUSG00000035735	Dagla	2.703791	2.489132	1.986326	1.269086	1.578053
ENSMUSG00000041488	Stx3	0.678666	0.40359	0.394204	0.507488	2.786635
ENSMUSG00000038843	Gcnt1	1.226999	1.109854	1.183807	1.715082	0.524374
ENSMUSG00000024747	Aldh1a7	8.885526	5.24172	5.063856	9.511558	3.081215
ENSMUSG00000038658	Ric1	4.105892	3.216221	2.333287	4.613915	2.507877
ENSMUSG00000024817	Uhrf2	0.947533	0.796454	0.678335	0.889846	2.801853
ENSMUSG00000052595	A1cf	0.782389	1.261579	0.899469	0.507522	0.489774
ENSMUSG00000039652	Cpeb3	0.489509	0.498404	0.371531	0.34796	0.399904
ENSMUSG00000025007	Aldh18a1	3.616965	8.553456	6.48488	3.253737	5.182472
ENSMUSG00000025189	Cnnm1	0.328647	0.087126	0.120892	0.353949	0.103833

ENSMUSG00000025192	Entpd7	1.218664	1.37559	0.904238	1.645222	1.318303
ENSMUSG00000025196	Cpn1	2.445822	3.561361	3.19583	1.554456	1.489632
ENSMUSG00000025203	Scd2	2.797874	3.032602	1.597993	2.423871	3.614118
ENSMUSG00000025221	Kcnip2	0.354784	0.557839	0.419081	0.339015	0.112892
ENSMUSG00000025036	Sfxn2	2.974678	2.826566	2.590832	2.698738	2.635094
ENSMUSG00000101088	1810018F1	23.74845	40.63622	40.47862	25.38278	9.622789
ENSMUSG00000042179	Pnliprp1	9023.412	10682.67	9079.098	9173.537	6741.1
ENSMUSG00000025091	Pnliprp2	2102.415	2059.497	1856.919	2058.849	1289.285
ENSMUSG00000031150	Ccdc120	0.270714	0.447989	0.158979	0.298227	2.083231
ENSMUSG00000031170	Slc38a5	14.65378	11.68917	12.82338	11.34117	1.439757
ENSMUSG00000040229	Gpr34	0.236025	0.280083	0.32949	0.076194	0.104309
ENSMUSG00000031099	Smarca1	4.048756	0.586042	0.67968	4.149021	0.228055
ENSMUSG00000001173	Ocrl	3.105267	0.879355	0.954899	2.484537	1.084747
ENSMUSG00000035967	Ddx26b	2.750825	1.820874	1.462207	1.882616	1.464926
ENSMUSG00000023092	Fhl1	1.28076	1.211646	1.169544	1.011898	1.24441
ENSMUSG00000078317	F8a	1.448789	1.34367	1.289932	1.322277	4.734352
ENSMUSG00000032806	Slc10a3	0.539935	0.831906	0.510603	0.960353	5.000248
ENSMUSG00000031196	F8	0.36996	0.73504	0.213919	0.231513	0.51943
ENSMUSG00000034457	Eda2r	0.173454	0.111788	0.06744	0.064877	2.738496
ENSMUSG00000059327	Eda	2.054737	1.120356	0.877042	1.72195	1.264917
ENSMUSG00000031231	Cox7b	13.06079	16.18334	19.0681	11.0711	13.86334
ENSMUSG00000025289	Prdx4	31.32011	33.47617	30.91278	27.17606	17.8049
ENSMUSG00000031364	Grpr	0.963624	0.95706	0.860818	0.845774	0.483883
ENSMUSG00000035299	Mid1	0.367032	0.440989	0.299543	0.265409	1.875115

8-wt-faee-2	11-cko-faee-1	12-cko-faee-2
2.331864	0.555271	0.491372
6.587449	0.925728	1.768766
2.684511	5.284397	3.443965
2.148072	4.469426	5.639267
1.718451	0.297242	0.169782
3.63837	5.277913	3.789547
4.577698	0.527412	0.572615
0.632367	1.267265	1.636999
5.135929	6.511992	7.046249
0.094321	0.419696	0.574346
3.112239	5.155697	5.692888
0.948979	0.123401	0.027534
3.860996	0.613121	0.387602
0.372678	0.718595	0.937338
14.7333	1.206292	1.181046
41.46151	7.720973	9.560018
1.439114	0.437955	0.265361
5.860682	0.464535	0.396028
0.207438	0.585648	0.409056
0.597989	1.362769	1.063079
131.2516	10.56763	17.0356
2.276066	0.232599	0.08073
8.195358	1.797796	2.071277
4.407433	1.213786	1.658368
4.818431	1.487467	0.829716
5.393093	0.480496	0.63026
0.096736	1.47945	0.819552
1.85446	0.385902	0.161215
0.193635	1.309654	1.148343
28.99975	6.476632	7.893282
0.453251	0.082741	0.073845
0.009838	0.116732	0.130228
2.987035	0.197554	0.134829
2.333561	5.907105	3.695118
1.741529	0.982747	5.088321
3.642771	4.108983	3.619589
10.56077	1.303454	2.061785
500.6419	22.7238	66.9818
814.2416	55.563	64.18695
0.247985	0.62402	0.534291
0.024567	0.165185	0.112737
0.398725	0.464192	1.410164
17.25077	29.03066	32.53298
2.77939	3.926848	5.612679
1.260467	0.373911	0.254256
32.7979	3.005808	4.790065
2.533939	6.31968	4.385468
7.731449	0.841523	1.3051
2.607688	0.25032	0.347522
5.115352	19.71905	17.26902
0.567519	0.181084	0.080808
2.546027	0.627013	0.220449
0.293962	1.017088	0.84664
2.770261	2.697458	3.314438

0.857024	1.140608	1.246921
1.48687	1.983728	2.530336
2.759306	6.610635	3.450889
19.49825	2.304504	3.210882
9.794099	1.321705	1.126469
18.36808	1.696925	3.300349
0.461553	0.854363	1.564119
15.16464	4.083975	2.329444
62.43939	5.7331	9.687485
3.572205	0.962411	0.438608
0.825622	0.048636	0.031005
0	0.391858	0.326413
5093.372	8063.259	8207.652
2.603813	0.564194	0.599447
0.351313	1.118244	1.065931
1.015438	4.917284	7.24654
0.239877	0.455413	0.533465
5.888457	0.477262	0.204456
0.339745	0.693392	0.388576
60.87786	340.6898	251.4631
3.428588	0	0.030901
2.769573	0.012038	0.064461
0.391124	0.60613	0.565958
15.2181	1.52459	1.784784
30.49232	4.594483	2.562823
56.50973	12.70264	7.434062
1.821976	14.42	2.681984
0.338993	0.561863	0.786373
5.995075	10.96462	9.181134
0.140148	0.453536	1.069281
0.534052	2.014244	2.242059
9.2391	11.97721	17.73881
12.37623	19.6297	27.13508
13925.75	19640.23	17780.96
13911.72	19614.6	17796.42
13929.04	19649.77	17776.43
40764.92	55064.12	54814.43
32.30859	38.01075	76.06883
4.797522	10.79004	12.99129
1.311127	0.412948	0.128564
0.680264	0.232132	0.12713
12.49517	1.155312	1.122081
1.121026	2.201394	2.044367
13.04118	2.219688	2.188581
28.96248	2.151143	2.865034
0.297066	0.632055	2.625239
0.032451	0.551913	0.114552
1.18808	1.54439	2.926339
1.049466	1.644877	1.617323
3.41807	6.259653	6.661018
0.481404	7.893274	3.406457
23.0926	58.19529	45.40509
0.794089	3.226489	1.605441
0.017207	0.020417	0.34622
10.53832	2.084093	2.678842
1.25107	1.36331	5.515875
1.166095	0.228077	0.176415
5.949656	0.233766	0

1.165622	0.925005	2.771506
30.69282	6.922794	3.646331
51.4516	7.636198	6.98559
4.206425	0.754346	0.561037
1.135137	0.253383	0.452282
6.600779	1.185832	1.332725
42.69263	3.284273	6.523679
12.71352	1.634703	2.343512
14.42611	1.376204	1.934906
6103.137	7918.198	8333.983
1.145188	2.558698	1.825594
1.007799	1.554584	2.401349
14861.31	22306.39	21488.48
22.21795	70.43762	63.24965
0.20726	1.762502	1.134031
63.86032	5.411536	6.398461
2.127204	0.424614	0.252642
9.349629	2.894107	1.434974
2.737067	4.241719	3.78568
21.44314	20.90998	45.83517
0.283859	0.051033	0
11.4255	15.76614	18.80967
9.520828	61.56394	88.40739
1.725216	2.14946	2.959765
1.174667	1.876317	1.605479
0.946706	1.003838	2.581081
4.986264	13.27276	6.706605
4.245253	0.408432	0.069432
3.588738	0.266145	0.203598
4.63608	0.110022	0.114558
3.984695	0.896662	0.888849
19.25116	5.671371	4.442983
1.001728	1.586836	2.06556
0.442146	0.870682	0.677448
23.37249	70.14058	72.4748
535.0065	736.1603	750.8533
7.186414	0.808537	0.604138
5.003912	0.895412	0.82242
17.77362	1.376723	2.546453
79.04516	11.79972	15.48842
177.9755	14.40916	20.26233
22.25265	4.789444	3.986414
1.832526	0.443132	0.344904
4.608897	6.729127	7.893727
11.24919	2.637084	3.245222
4.190193	0.967524	0.829922
4.351882	6.349949	4.415031
3.303585	21.78796	18.49218
0.339757	0.855916	0.65872
0.568006	3.341442	3.354969
879.811	1341.01	1500.342
8543.573	12265.73	11858.91
6.294677	1.764553	0.906073
0.501227	4.48347	5.471357
4.639304	27.48343	23.09869
4170.046	7087.202	7648.475
31601.12	41222.62	45270.97
16873.07	26151.9	32015.5

16.9136	40.13865	30.67668
8.679843	13.3188	12.40368
1.61258	0.292503	0.163159
6.847508	0.950506	0.689895
4.881656	0.410814	0.531637
8.224245	16.08381	13.38687
0.077503	6.805326	3.447216
0.834326	3.167979	2.827381
0.080359	2.955912	0.680804
0.570011	3.478441	2.500818
1.938814	13.70748	1.368811
3.958637	1.14179	0.735252
4.20345	8.001146	5.842571
3.002588	0.553855	0.523215
42.93733	3.650593	6.891135
0.054383	0.483977	0.863887
0.916397	0.051085	0.091185
3.713721	1.001196	0.844926
40.43616	7.160049	6.964376
4.926223	0.76668	0.767192
3.386887	4.25521	3.385342
18.71923	5.579963	3.58242
32.51146	48.8449	39.54287
1.61935	3.374713	3.386573
1.487421	1.922215	2.331592
3.220674	0.627424	0.712687
4.959704	0.959006	1.079179
2.527974	0.047613	0.212472
33.40797	4.306114	4.728747
25.68436	0.845189	1.357777
8.129468	1.519286	1.065384
0.918349	0.268053	0.208029
0.23363	0.338825	0.833883
0.046514	0.800289	0.353019
1.457625	2.014203	1.597369
8.038712	17.22064	18.85441
1.815975	3.762609	4.674685
8.833664	1.484072	1.591085
1.560082	0.068057	0.242961
2.345136	0.352735	0.157406
0.040661	0.203328	0.12918
12.1874	4.943058	3.898637
0	4.495882	0.136018
9.659788	0.168853	0.168428
3.224015	0.617415	0.756322
0.588001	0.075428	0.075733
128.508	0.987182	1.588367
46.03011	1.66592	1.327511
41.402	0.778026	0.198394
0.014316	0.509617	0.333539
1.159786	2.886617	2.231768
5.895651	1.330424	1.092619
5.952181	16.12337	11.7309
1.241196	2.632625	1.919126
6.054911	1.078921	0.970218
0.816611	0.048938	0.043677
4.724561	0.799188	0.220273
4.645821	1.047401	0.899649

2.166738	0.348611	0.252794
2.297473	0.431039	0.485933
0.879985	2.83	3.057015
0.411327	0.919829	0.938212
4.481392	0.486359	0.28938
1390.848	202.37	271.6072
5.97758	5.866647	19.82778
24.60162	6.143008	5.593991
1460.472	4645.877	5021.832
6.190497	1.597187	1.728467
5.685386	1.084972	1.19842
7.185815	0.534588	0.4891
5.061566	1.433039	1.216581
0.830382	1.947169	1.251023
0.118146	0.335086	0.314318
15.6688	3.422846	3.47913
0.860178	2.721785	1.239883
1.891375	10.94722	3.944798
0.04781	0.243941	0.091136
5.856157	9.007894	10.00536
0.420225	2.169963	1.516367
16.86176	3.279712	3.37527
0.521285	1.123149	1.61255
58.77167	14.37508	11.00936
0.231121	0.598859	0.449557
0.027011	0.272434	0.243144
38.45904	4.361909	4.089703
3.106122	2.79314	5.375368
0.6752	1.201767	1.107442
2.565102	4.129639	6.879881
2.107324	0.322392	0.463177
5216.77	7898.96	7848.888
7.378863	15.53483	8.705701
1.456277	3.536977	4.554332
47.54693	8.642552	7.996167
51.81708	124.1766	116.1352
155.7048	19.69619	3.564045
4.212226	0.926808	0.8764
4.055264	0.986128	0.843244
50321.69	70994.32	84249.37
0.671048	2.319515	1.374942
0.167628	0.020719	0.011095
43.63148	2.781993	6.32835
0.004635	0.044003	0.018409
8.114966	3.052766	1.247693
0.248999	0.778932	0.910934
21.38943	3.459505	4.486506
5.327903	1.073266	0.787296
8.015861	2.366769	1.940686
4.752734	5.287027	6.039814
1.0291	0.746233	6.518761
1.592174	2.029184	5.068776
0.247258	1.44495	3.574219
0.025461	0.38268	0.134817
1.412084	2.432769	2.128079
28.36324	3.330247	2.754816
3.844946	0.662274	0.446587
2.499126	3.623376	7.042625

0.031743	0.15066	1.176545
4.237176	1.085313	1.009474
4.822073	0.818282	0.735839
5.200046	1.610544	0.90848
5.172682	0.23721	0.740973
0.030486	0.373793	0.247513
18.5162	2.939773	2.334977
0.600622	0.899183	1.236459
0.735418	2.526725	0.895054
0.754691	1.751327	1.021791
0.978366	1.875315	2.231593
0.324148	0.71828	0.541796
0.494115	1.045971	1.26841
0.555354	0.440385	1.73855
27.45884	3.797387	2.509326
3.150908	12.16993	7.009589
4.202455	0.880776	0.520023
41.21461	5.446236	9.646612
0.111373	1.117288	0.053611
0.496525	4.892017	2.025715
1.302883	2.492883	1.241786
5.78539	21.91147	25.6237
0.901441	1.018697	1.733112
0.407529	1.031403	1.043431
0.054594	0.367088	0.30835
39.73883	1.373221	4.370168
6.774165	1.520879	1.468712
0.177974	0.828479	0.304462
0.702188	3.542857	0.98733
9.448224	2.06816	1.553402
12.60576	2.024522	2.34958
2.430432	0.408099	0.378792
0.993051	0.117833	0.052582
2.282812	0.158303	0.156981
21.08385	3.217885	4.631596
0.072914	0.484501	0.293422
0.379202	1.049892	1.617466
0.059113	0.136592	0.125201
2.698829	21.0777	16.9144
8.107151	2.256355	2.20041
5.535647	8.478343	6.881817
2.617398	4.425188	8.134363
0.59758	3.332655	1.328966
2.654328	0.483847	0.472566
6.257688	9.186359	6.457014
16.6206	33.08475	21.61883
2.192555	0.094605	0.042217
3.447649	0.166976	0.447071
70.86924	7.759016	9.36149
3.98161	0.142019	0.2366
4.147966	0.839392	0.919407
10.88706	1.211653	1.590271
8.225018	1.307208	1.631067
11.37274	2.081338	2.35668
0.354588	2.153236	2.352469
10.0729	0.818355	2.229556
1.123771	0	0.022886
1.879565	2.591491	2.551169

5.855192	0.60056	0.430895
0.236674	0.982913	0.552546
44.56052	4.813619	3.826208
9.814547	2.546782	1.405109
26.07142	3.217567	4.151315
6.235347	1.554356	1.072801
0.885842	2.047782	2.444927
1.048422	1.890542	1.975784
1.666687	1.856351	2.237459
16.91142	3.352204	5.010918
12.47876	1.067914	1.338005
16.82613	5.502117	1.890401
7.941847	1.698564	2.037432
0.620815	3.499067	1.446383
0.276138	3.986533	3.314236
17.00381	5.498323	2.895068
0.148415	0.275165	0.726921
0.54062	17.09257	3.472059
2.087037	0.546162	0.353897
33.90695	10.44637	7.835951
0.097787	0.358646	0.385987
10.34462	1.586108	1.643647
1.535769	0.653744	0.765789
12.40603	17.68464	32.51452
0.195567	4.309597	5.50311
0.57547	0.838424	1.797422
3.503437	0.140919	0.45591
30.26986	0.779901	1.034671
268.0952	24.64738	24.30063
2.058628	0.799702	0.32442
10.86907	1.399907	1.608269
26.50435	4.015063	6.222249
0.941717	3.077488	2.256151
3.234285	5.359001	3.363416
15.49707	19.54287	19.75683
1.911398	11.75001	7.535818
3.956722	12.3395	5.011329
0.704007	3.028182	1.957065
0.063617	1.887175	1.414795
0.147876	1.228263	0.783007
0.782982	3.613053	1.750499
1.574037	4.856071	8.33458
4.175906	9.910089	5.067235
0.236106	3.081737	2.417028
1.391969	7.845487	1.750499
1.177304	3.841652	2.727315
0.85297	5.465428	1.174291
0.260994	14.7619	6.909865
0.225789	1.615621	1.956376
2.452987	4.144852	4.804485
1.845812	3.14444	2.676598
0.051356	0.377816	0.217546
0.640648	1.162628	1.12144
13.44838	39.68867	46.43508
24.65831	1.458418	1.463479
1.715006	0.081946	0.109704
0.258417	1.839796	1.049052
2.603266	0.489089	0.381368

0.645816	0.68968	4.18333
0.209583	2.561481	0.599266
0.697198	1.081394	0.922292
6.389464	0.979291	0.789423
35.78278	7.641894	7.718575
0.067524	0.394448	0.126514
1.05	2.969701	2.711349
1.876565	0.413338	0.470049
0.633552	2.085359	1.815202
0.989565	0.048925	0.061131
0.638211	1.448245	1.312274
10.80769	1.108531	2.282618
30.24484	36.45755	54.99849
1.728471	0.027346	0.048812
4.786982	0.801632	1.08339
5.049687	1.158802	0.882867
2.310011	5.209013	3.099318
18.95193	23.15437	44.13233
2.533179	0.054651	0.146327
20.67759	3.864152	4.413086
1202.06	6728.834	7049.721
2.194226	0.192861	0.172126
2.972285	4.714844	7.380458
11.22228	0.999252	1.008144
0.628655	0	0.012329
2.380678	4.978471	7.40121
0.656443	2.364583	2.02967
2.367988	3.475543	2.638781
25.48039	21.66879	75.01759
2.183127	0.45618	0.319892
1.691606	2.53191	5.091124
24.65807	77.54025	50.60923
4.731542	0.66151	0.756909
14.87565	22.4591	25.72946
18.0305	2.34072	2.993672
0.438164	1.642715	0.878674
0.942687	0.066582	0.106962
5.177387	1.757738	1.312476
1.09095	0.244245	0.250683
8.479989	20.71917	17.82802
1.195781	0.288587	0.150244
0.23253	0.668744	0.943266
36.03794	8.845865	8.555784
0.373583	0.992574	0.980466
1.620698	0.078174	0.083723
0.696405	0.033387	0.059596
3.629488	0.561956	0.621376
10.28915	0.813926	1.1773
4.644191	8.704267	4.950376
4.537115	8.58964	8.583921
0.458045	0.600718	1.182896
1.943769	5.760116	4.116931
3.377558	0.517535	0.721004
12.50583	1.43028	0.95738
1.660367	0.12237	0.109214
14.50584	2.475313	1.455705
1.114653	4.936773	3.060871
10.16023	2.155986	2.748843

3.897762	0.082101	0.122123
3.722792	0.195707	0.124761
3.778554	8.967109	6.106015
0.150302	1.515933	0.769325
4.73602	15.16002	12.5387
9.879396	1.299918	2.090379
11.35252	16.61271	22.90123
0.776839	6.538617	9.026393
0.003526	0.092049	0.014937
2541.732	11600.34	11159.96
9.881743	0.071016	0.176057
1.114163	6.806071	2.993466
361.939	586.6891	504.6194
12.13049	16.6374	17.3444
8.602327	1.10849	2.122901
43.47054	11.41998	8.628305
0.633456	0.132643	0
21.81805	3.187721	5.242929
13.04071	3.459988	1.772738
19.49553	5.661134	4.012929
6.254849	26.35183	33.78948
8.225522	1.80315	1.7373
6.733649	1.168673	1.557993
30.43503	33.34948	57.75822
0.049392	0.293035	0.124227
11.69448	2.499971	2.513622
10.08113	0.849606	1.387276
28.0138	2.828987	3.719622
45.72963	8.330213	12.58652
10.73718	1.851133	1.635481
2.429879	4.518074	3.926209
24.43682	2.512392	2.674276
1.757526	0.251577	0.230438
4.776398	0.782587	0.628604
4.369934	0.84901	0.544814
10.63152	1.275428	1.107259
0.16299	1.041649	0.399723
0.021272	0.189309	0.777197
11.02224	39.43984	31.29354
9.62897	2.129064	2.773529
2.034284	0.183164	0.198501
47.85072	5.069525	6.262841
20.63538	4.765234	3.771449
115.1313	33.58016	10.75427
27.60296	1.540159	3.568843
0.077084	0.810131	0.303207
1.883208	5.238037	3.98866
2.111742	3.910646	3.246917
1.110508	2.206685	1.755613
4.480727	0.761347	0.804066
0.515242	1.034135	0.684959
4.38978	5.927885	8.796733
2.320043	2.320555	5.417329
3.09629	0.936425	0.602341
0.063817	1.287302	0.371703
0.461299	0.54248	0.963951
2.467202	8.284291	4.455234
0.06666	0.186957	0.410725

0.889209	1.188522	1.55864
1.622863	4.018332	2.779609
2.183965	6.664673	4.287639
0.140558	0.854767	0.390737
1.16057	3.487923	3.00211
0.451493	25.03566	8.571422
5887.04	7993.248	9115.569
1376.141	1891.023	2209.727
2.881338	0.347983	0.442563
1.75778	6.370416	6.421457
0.102275	0.762816	0.123782
0.064886	0.361272	2.864872
0.882031	0.78304	3.44995
1.132394	1.464909	1.794311
1.093519	1.999979	1.41055
6.546108	1.084843	1.252217
6.14349	0.913456	0.751307
0.345287	0.903558	0.47993
1.622452	0.09226	0.159193
1.064856	1.205138	1.866848
12.02336	19.78829	15.71624
19.25557	26.04839	22.649
0.26627	0.646262	1.038205
2.899132	0.382827	0.298358
